# Serendipitous Discovery of Dearomatized Dimers in Anthracene Derivative Oxidation

**DOI:** 10.1021/acs.orglett.4c04417

**Published:** 2025-01-14

**Authors:** Xinhao Fan, Huan Chen, Baotong Tian, Yuming Wen, Qiang Zhang

**Affiliations:** † Department of Chemistry, School of Pharmacy, North Sichuan Medical College, Nanchong, Sichuan 637000, China; ‡ Department of Chemistry, University at Albany, State University of New York, Albany, New York 12222, United States

## Abstract

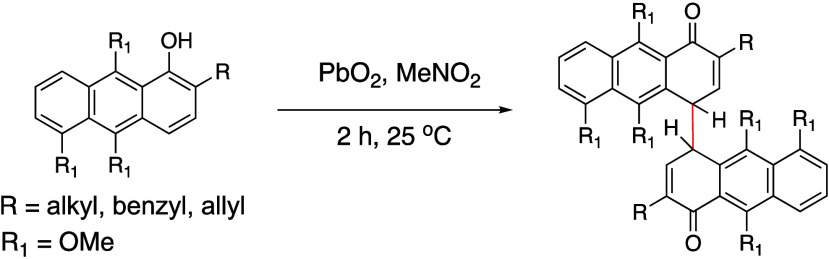

We present the serendipitous discovery of an unusual dimer formed from anthracene-derived polyarenes. Unlike the typical oxidative coupling of substituted aromatic scaffolds, the reaction yielded a dearomatized enone dimer as the sole product. This dearomatized motif, notably, does not undergo the commonly observed rearomatization, and no biaryl products were detected. The anthracene dimers were produced in excellent yields. Structural validation via single-crystal X-ray analysis revealed that the dimers feature an sp^3^–sp^3^ carbon–carbon bond connecting two α,β-unsaturated enones, existing as a pair of diastereomers. These unique dimers underscore the critical role of serendipity in advancing organic synthesis.

Natural products are highly valued for their remarkable scaffolds and significant biological activities. Many of these compounds are targeted in synthetic chemistry due to their unique structural frameworks and potent bioactivities.^1^ Many natural products containing poly-aromatic ring motifs have garnered a significant amount of interest due to their highly functionalized and intricate scaffolds.^2^ Our ongoing interest in aromatic substrates has motivated our exploration of natural product synthesis and their complex structural features.^3^ The specific focus of this study is the urdamycinone family of natural products. These compounds were predominantly isolated from plant sources and are characterized by a distinctive anthraquinone-based core structure. This core imparts unique chemical properties and serves as a fundamental scaffold for their biological activity, making them an intriguing subject for further synthetic and functional exploration.^4^ Urdamycinone and its derivatives exhibit activity against Gram-positive bacteria; they are produced by type II polyketide synthases (PKSs) and undergo enzymatic modifications that disrupt bacterial DNA and RNA. Structurally, their tetracyclic structure, with hydroxyl and methyl groups on aromatic rings (Figure 1a, **1** and **2**), forms the basis for the glycosylated urdamycin derivatives, which are active against Gram-positive bacteria. Research into their biosynthesis aims to engineer PKS systems for improved antibacterial and anticancer properties.^5^ Over the years, numerous research groups have developed sophisticated approaches for the total synthesis of these molecules.^6−9^ However, the continuous exploration of more efficient and convergent synthetic strategies remains essential to advancing chemical synthesis. A detailed examination of their tetracyclic scaffolds reveals that the carbohydrate moiety is generally linked to the aromatic A ring of the tetracyclic core through C-glycosylation. Additionally, the B ring features a quinone functionality, while a *cis*-diol group is situated between the C and D rings. Our proposed strategy for the synthesis of aquayamycin **2** is depicted in Figure 1b. We hypothesize that the tetracyclic core of **2** can be constructed via the pinacol condensation^10^ of the carbonyl groups in compound **3**. Compound **3** could be produced from dearomatization of *ortho*-alkylated anthracene.^11^

**Figure 1:**
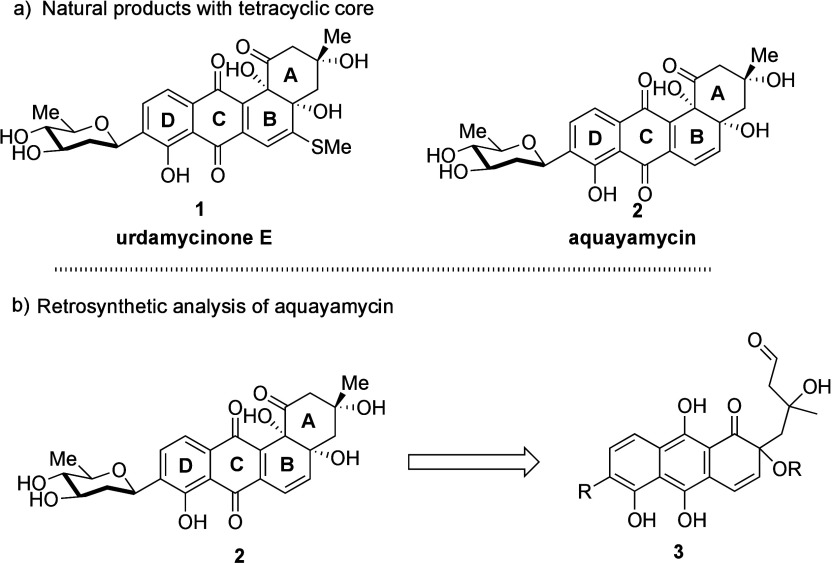
Examples of natural products.

The synthesis of aquayamycin **2** commenced with the transformation of commercially available 1-hydroxyanthraquinone **7** into O-allylated anthraquinone derivative **8** under basic conditions (Scheme 1). A two-step reduction of quinone **8** by sodium dithionite^12^ followed by methylation successfully furnished hydroquinone **9** in 78% overall yield. The subsequent Cope rearrangement of compound **9** efficiently yielded C-alkylated hydroquinone **10** in 83% yield. The proposed B ring dearomatization should generate *ortho*-oxidized ketone **11** under the PIDA/HOAc oxidation conditions. Although *ortho*-oxidative dearomatization is well-established for similar phenolic structures,^13^ the oxidation of compound **10** did not yield anticipated product **11**. Instead, unexpected enone dimer **12** was isolated. Dimer **12** consists of two dearomatized enone units linked in a *para*-to-*para* configuration. Under hypervalent iodine oxidation conditions, hydroquinone **10** underwent dearomatization. However, contrary to expectations, no acetate group was incorporated into the hydroquinone framework, despite the use of the reagent HOAc as the solvent. According to ^1^H nuclear magnetic resonance (NMR) analysis (Scheme 1b), the structure of anthracenol dimer **12** features chemically equivalent protons from the monomer that completely overlap. The coupling constant between methine protons H_A_ and H_B_ is small (*J*_AB_ = 0.4 Hz), suggesting a nearly 90° dihedral angle between H_A_ and H_B_. Alternative dimer structures, such as *ortho*–*ortho* dimer **12a**,^14^ were excluded, as was rearomatized biaryl product **12b**, based on ^1^H NMR analysis (Scheme 1, inset).

**Scheme 1:**
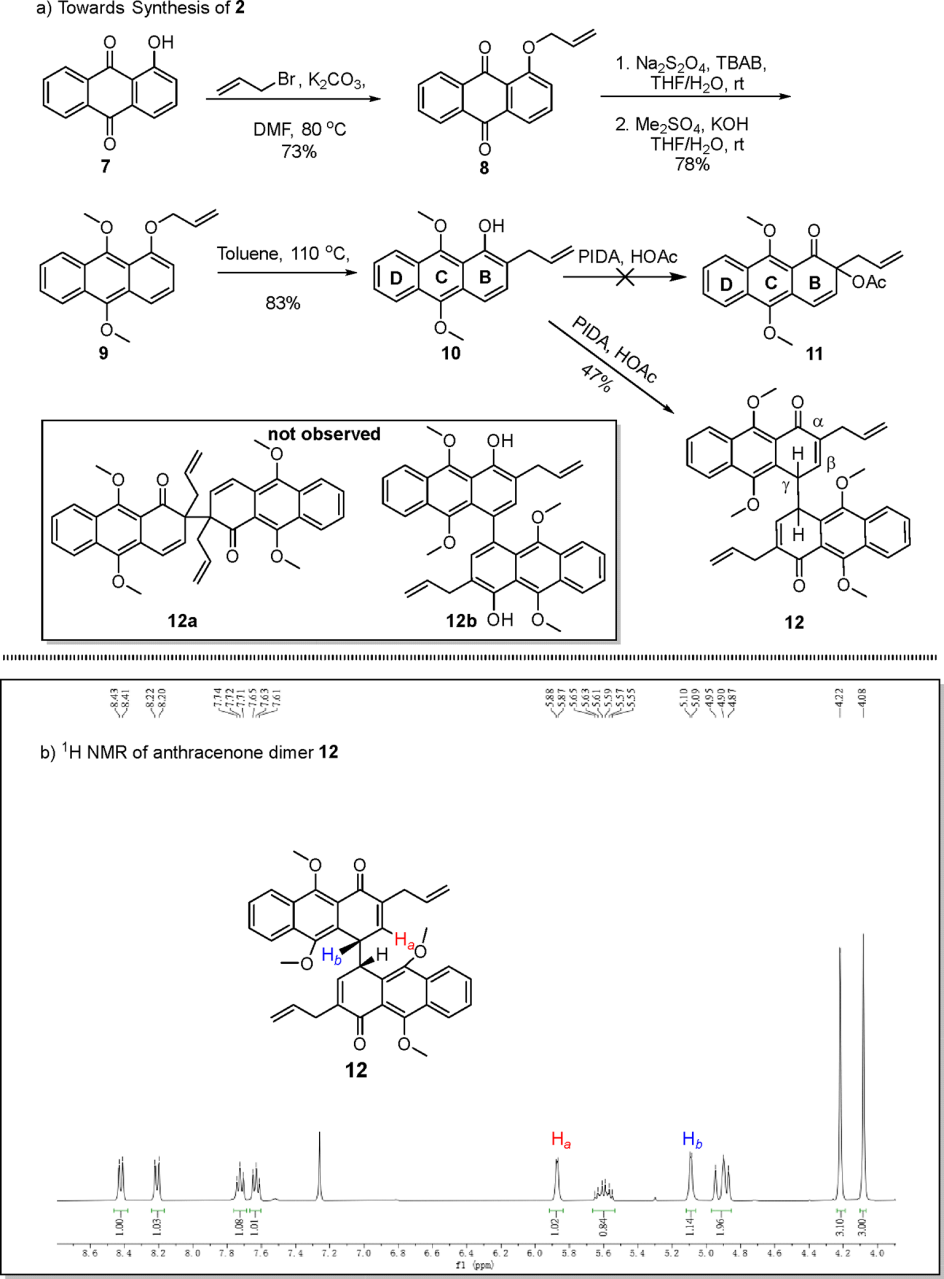
Unexpected Enone Dimer Formation via Oxidative Dearomatization

Consequently, we performed a comprehensive assessment and optimization of the reaction, testing a range of solvents and oxidants (Table 1). Other commonly used oxidants were also tested. When iron(III)-based reagent K_3_Fe(CN)_6_ in a MeOH/Et_3_N solution was combined with **13** and reacted for 8 h at room temperature, no product formation occurred. A stoichiometric amount of silver perchlorate was used, and after 30 min in toluene, compound **13** was completely consumed. However, none of the desired product **14** was observed, and the only product isolated was quinone byproduct **14a**(15) (43% yield). Hypervalent iodine-based oxidants, IBX and PIDA, were subsequently tested (entries 4 and 5, respectively). While IBX in DMSO failed to yield any product, the use of PIDA in toluene produced a mixture of the desired product **14** and byproduct **14a** in a 3:1 ratio. MnO_2_ in toluene successfully delivered the desired product **14** in moderate yield, whereas oxidation with Mn(AcO)_3_ resulted in only trace amounts of dimer **14** in toluene. We later discovered that PbO_2_ (1.2 equiv) in toluene exclusively produced **14** in 72% yield. When the same oxidant was used in nitromethane, the product was obtained in nearly quantitative yield (95%) within 2 h. The reaction crude was clean enough to allow efficient purification using silica gel filtration. Lastly, atmospheric air alone failed to yield any product even after 8 h (entry 8), highlighting the necessity of an oxidant beyond oxygen.

**Table 1:**
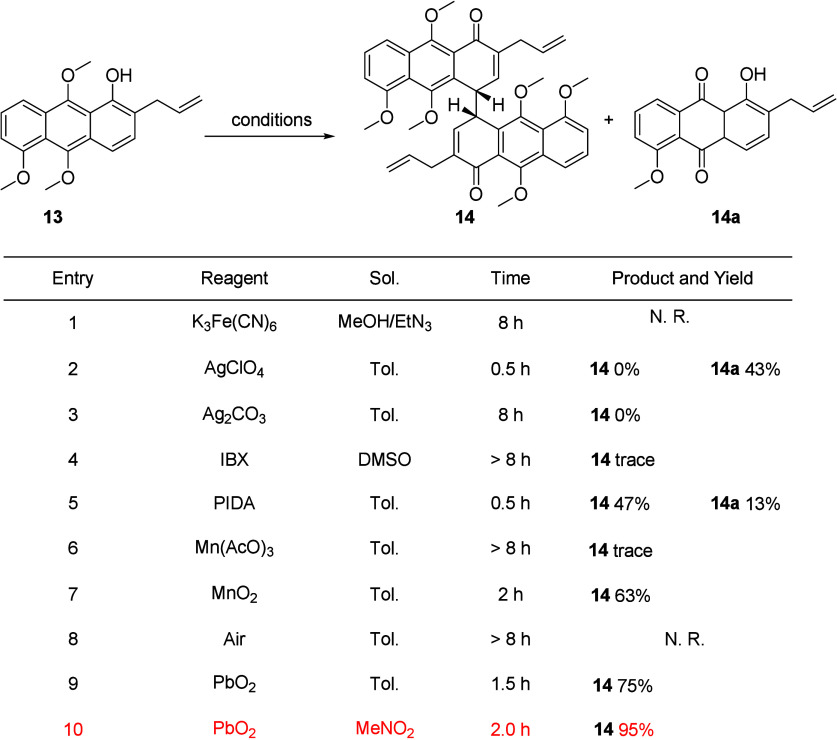
Dearomative Dimerization Substrate Scope

With the optimized conditions established, we synthesized seven more anthracene monomers to assess their reactivity in the dimerization reactions. Different monomers **13a** were prepared according to a similar procedure for making compound **13**,^16^ and the results are summarized in Figure 2. Substituting the anthracene A ring with an additional methoxy group did not affect the reactivity, affording dimer **15** in 96% yield. With the 1,4-dimethoxy functionality on the B ring retained, various α-substituents on the C ring of the monomer led to a diverse array of dimeric products. Introducing aromatic side chains at the α-position of the C ring was beneficial, affording enone dimer **17** in excellent yields (93%). Interestingly, electron-donating groups on the benzyl ring slightly enhanced the yield (**20**), whereas electron-withdrawing groups on the benzyl ring significantly reduced the dimer yield (**18** and **21**). Lastly, the aromaticity of the A ring is not essential, as compound **19** could be synthesized in 95% yield by replacing the aromatic A ring with cyclohexene. All of the dimers were obtained in good to excellent yields (89–96%), and they are stable at room temperature for days. The single crystals of two compounds (**15** and **19**) were obtained for X-ray analysis. The X-ray analysis definitively confirmed the structure of the enone dimers. Both compounds exhibited two stereocenters in the *RR*/*SS* configuration, with no diastereomers containing the mesocompound stereocenters observed (Figure 2, **19**). The ^1^H NMR data are consistent with the X-ray analysis of the anthracenol dimer scaffold, further confirming the exclusive formation of the *R,R* and *S,S* enantiomer pair. Methine protons H_A_ and H_B_ create a nearly 90° dihedral angle. Examining the X-ray structures of the dimers reveals that the monomer possesses an α,β-unsaturated enone system, with the γ position of the enone containing a tertiary carbon center at the H_A_ methine. While dearomatized enones commonly exhibit a quaternary carbon at the α-position, enones such as **15**–**21**, which incorporate γ-tertiary carbons, are rarely isolated due to their propensity to rearomatize into biaryl compounds, driven by the inherent stability of aromaticity.^17−19^ This approach represents a unique method for generating tertiary carbon centers. In this study, anthracene monomers lacking functional groups at the C ring α-position predominantly undergo oxidative dimerization, yielding fully aromatic *para–para* biaryl adducts.^20^ Experimental data and X-ray crystal structure analysis suggest that steric effects play a critical role in determining the formation of the observed dimers. The methoxy group on the B ring effectively hinders access to at least one of the methine hydrogens, implying that the observed dimerization outcomes are governed by a kinetic trapping mechanism.^21^

**Figure 2:**
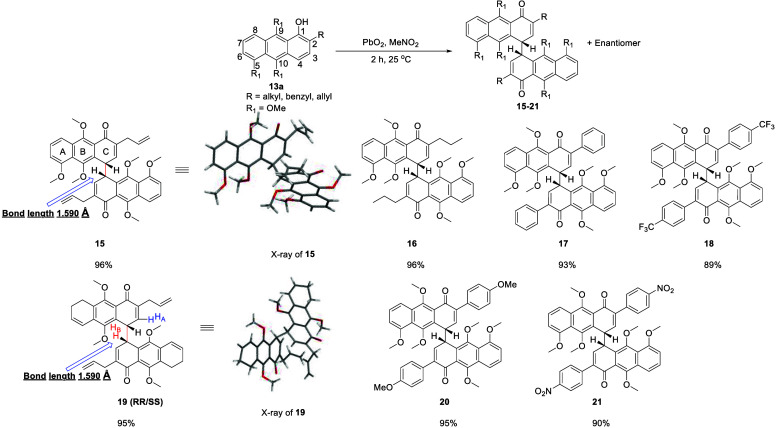
Dimer scope.

We identified a novel class of homodimers derived from anthracenols through stoichiometric metal-mediated oxidation. These reactions proceeded with remarkably high yields, exclusively producing a single pair of diastereomers. The resulting dimers exhibit unique *para–para* linkages incorporating tertiary C–C bonds and two chiral centers. Comprehensive NMR and X-ray analyses confirmed their connectivity and revealed intriguing structural features. This study presents a straightforward approach for synthesizing structurally distinct dimers,^22^ paving the way for further characterization and exploration of their properties. This report underscores the significance of serendipitous discoveries in organic chemistry, and ongoing investigations into the total synthesis of angucycline natural products will be detailed in future publications.

## Data Availability

The data underlying this study are available in the published article and its Supporting Information.

## Supporting Information Available

Complete experimental procedures and characterization data for all new compounds (PDF)
