# Supplementary material for: Serendipitous Discovery of Dearomatized Dimers in Anthracene Derivative Oxidation
Source: Org Lett. 2025 Jan 14;27(3):767–71. doi: 10.1021/acs.orglett.4c04417 (PMC11773568; doi:10.1021/acs.orglett.4c04417)
Supplement: Supplementary file 1 — ol4c04417_si_001.pdf [file ol4c04417_si_001.pdf]

Serendipitous Discovery of Dearomatized Dimers in Anthracene Derivatives Oxidation

Xinhao Fan, [1,2†] Huan Chen, [2†] Baotong Tian,[2] Yuming Wen,[2] Qiang Zhang[2*]

[1]Department of Chemistry, School of pharmacy, North Sichuan Medical College, Nanchong Sichuan 637000, China.

[2]Department of Chemistry, State University of New York, University at Albany, Albany New York 12222, United States

[†] These authors contributed equally.

Contents

1. General Information 3
2. Unexpected Enone Dimer Formation via Oxidative Dearomatization. 4
3. Oxidation conditions screening 7
4. Preparation of anthracenol monomers 7
5. Procedures for Dearomatized Enone Dimers 15
6. X-Ray Crystal Structure and Data 19
7. Mechanism 23
8. Reference 24
9. Spectrum data 24

1. General Information

General considerations, Analytical thin layer chromatography was performed using 0.25 mm silica gel 60-F254 plates. Flash chromatography was performed using 200-400 mesh silica gel. (Scientific Absorbents, Inc.) Yields refer to spectroscopically pure materials unless otherwise stated. All reagents were purchased from Sigma-Aldrich, Tokyo Chemical Industry, Acros, Alfa Aesar, Chemimpex, and Oakwood Chemicals which were used without further purification. All reactions were carried out in oven-dried glassware under an atmosphere of nitrogen gas with anhydrous solvents unless otherwise noted.

NMR (Nuclear Magnetic Resonance) experiment, all ^{1}H NMR spectra were recorded at a Bruker 400 or 500 MHz at ambient temperature unless otherwise stated. ^{13}C NMR spectra were recorded at 101 or 151 MHz at ambient temperature unless otherwise stated. All ^{13}C NMR spectra were recorded with complete proton decoupling. ^{1}H-NMR chemical shifts were recorded relative to the solvent residual peak ($CDCl_3$ 7.26 ppm, C_6D_6 7.16 ppm, DMSO-d_6 2.50 ppm, acetone-d_6 2.05 ppm, CD_2Cl_2 5.32 ppm, MeOD-d_4 3.31 ppm). ^{13}C NMR chemical shifts are reported relative to the solvent residual peak ($CDCl_3$ 77.1 ppm, acetone-d_6 29.9 ppm, CD_2Cl_2 54.0 ppm, MeOD-d_4 49.0 ppm). Multiplicities were reported as: s (singlet), d (doublet), t (triplet), q (quartet), h (sextet), m (multiplet or unsolved), br s (broad singlet), dd (doublet of doublets), doublet of triplets (dt), triplet of doublets (td) or doublet of doublet of doublets (ddd). The number of protons (*n*) corresponding to a resonance signal was indicated by *n*H and spin-spin coupling constants (*J* value) recorded in Hz.

High-Resolution Mass Spectrometry (HR-MS) were recorded on Agilent 6530B Q-TOF mass spectrometer (Agilent Technologies, Inc., United States) at the University at Albany-SUNY Core Facility Center.

Single Crystal Data collection of crystal samples was performed on a Bruker D8 VENTURE X-ray diffractometer with a PHOTON 100 CMOS shutterless detector and a Mo-target X-ray tube (λ = 0.71073 Å) at 100(2) K. Data reduction and integration were performed with the Bruker software package SAINT. (version 8.37A).[1)] Data were corrected for absorption effects using the empirical methods as implemented in SADABS (version 2016/2).[2)] The structure was solved by SHELXT (version 2018/2)[3)] and refined by full-matrix least-squares procedures using the Bruker SHELXTL (version 2018/3)[4)] software package through the OLEX2[5)] graphical interface. All non-hydrogen atoms (including those in disorder parts) were refined anisotropically. The H-atoms on carbons were included at calculated positions and refined as riders, with $U_{iso}(H) = 1.2\ U_{eq}(C)$ and $U_{iso}(H) = 1.5\ U_{eq}(C)$ for methyl groups. All ellipsoid contour % probability levels were set at 50% for compound **15** and **19**.

2. Unexpected Enone Dimer Formation via Oxidative Dearomatization.

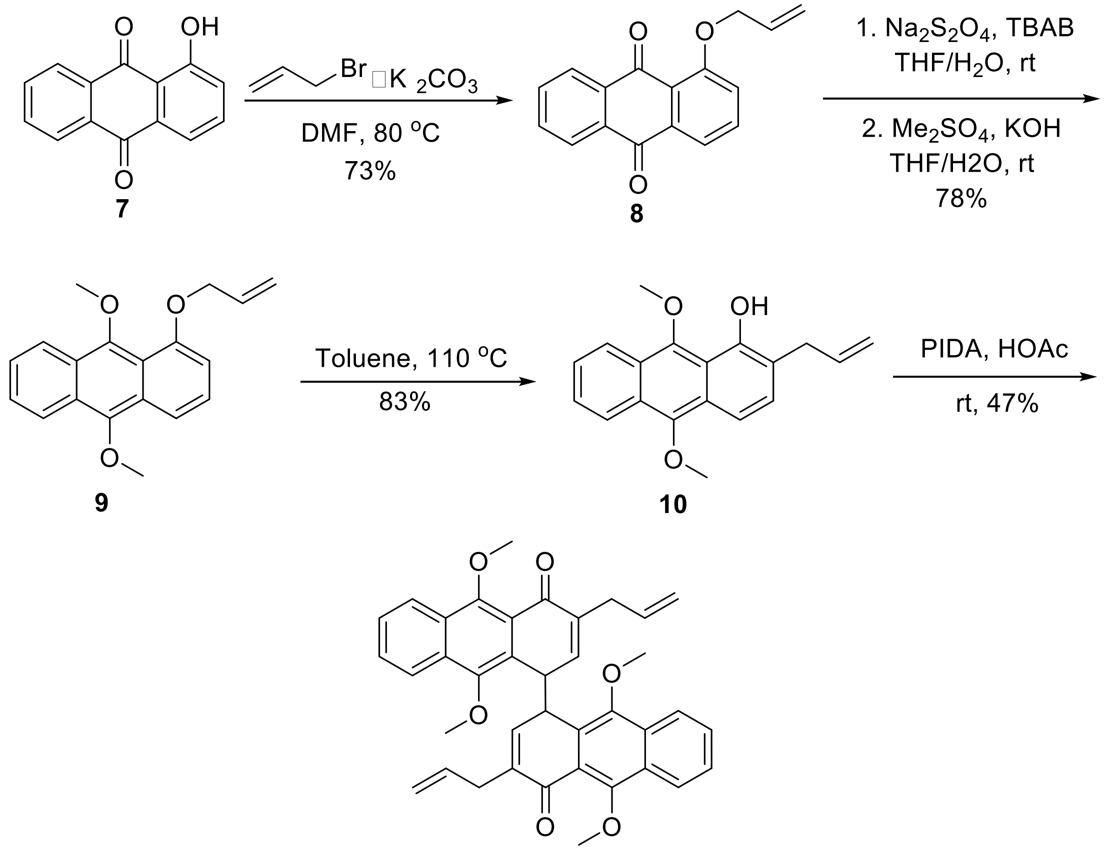

7 8 9 10 12

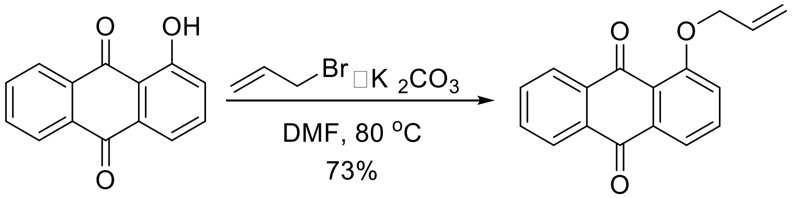

7 8

K_2CO_3 (247 mg, 1.78 mmol, 2.0 equiv.) was added into a DMF (5 mL) solution of 1-hydroxyanthracene-9,10-dione (commercially available) (200 mg, 0.89 mmol, 1.0 equiv.) and allyl bromide (155 μL, 1.78 mmol, 2.0 equiv.). The mixture was heated up to 80 °C by oil bath for 8h and then cool down to room temperature. The crude mixture was filtered through a pad of Celite. Then water (15 mL) and ethyl acetate (15 mL) were added. The organic layer was separated, and the aqueous layer was extracted with ethyl acetate (3 × 8 mL). The combined organic layers were washed with saturated aqueous NaCl (2 × 5 mL), dried ($MgSO_4$), and concentrated under reduced pressure. The crude residue was purified by flash chromatography to afford **8**.1-(allyloxy)anthracene-9,10-dione (**8**) (172 mg, white amorphous solid, yield = 73%). Hexane/ethyl acetate = 8/1, Rf = 0.2.

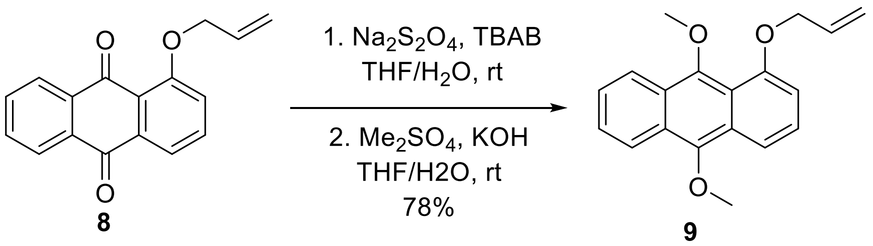

A solution of **8** (266 mg, 1.0 mmol, 1.0 equiv.) and TBAB (97 mg, 0.3 mmol, 0.3 equiv.) in a mixture of THF (5.5 mL) and water (5.5 mL) was added $Na_2S_2O_4$ (1044 mg, 6.0 mmol, 6.0 equiv.). After stirring at room temperature for 30 min, the mixture was added KOH (563 mg, 10.0 mmol, 10 equiv.) followed by addition of dimethyl sulfate (472 µL, 5.0 mmol, 5.0 equiv.). Then the mixture was allowed stirring at room temperature for additional 3h before quenched by saturated Sodium thiosulfate solution. Then water (15 mL) and ethyl acetate (15 mL) were added. The organic layer was separated, and the aqueous layer was extracted with ethyl acetate (3 × 8 mL). The combined organic layers were washed with saturated aqueous NaCl (2 × 5 mL), dried ($MgSO_4$), and concentrated under reduced pressure. The crude residue was purified by flash chromatography (Hexane /ethyl acetate = 10/1 to 4:1) to afford **9**.

1-(allyloxy)-9,10-dimethoxyanthracene (**9**) (232 mg, white amorphous solid, yield = 78%). hexane/ethyl acetate = 8/1, rf = 0.2. ^1H NMR (500 MHz, $CDCl_3$-*d*) δ 8.47 (dd, *J* = 7.4, 2.1 Hz, 1H), 8.36 – 8.28 (m, 1H), 7.97 (dd, *J* = 9.0, 4.1 Hz, 1H), 7.61 – 7.48 (m, 2H), 7.37 (dd, *J* = 8.8, 7.4 Hz, 1H), 6.79 (d, *J* = 7.4 Hz, 1H), 6.30 (ddd, *J* = 17.4, 10.7, 5.3 Hz, 1H), 5.65 (dt, *J* = 17.2, 1.8 Hz, 1H), 5.42 (dt, *J* = 10.5, 1.5 Hz, 1H), 4.76 (dd, *J* = 5.3, 1.7 Hz, 2H), 4.12 (s, 3H), 4.07 (s, 3H). ^{13}C NMR (126 MHz, $CDCl_3$-*d*) δ 155.5, 149.3, 148.0, 133.4, 127.3, 126.3, 126.0, 125.3, 125.3, 125.2, 123.5, 122.2, 118.5, 117.9, 115.3, 105.3, 70.1, 63.8, 62.9. HRMS (ESI+): calculated for $C_{19}H_{19}O_3$ $[M+H]^+$ 295.1329, found 295.1343.

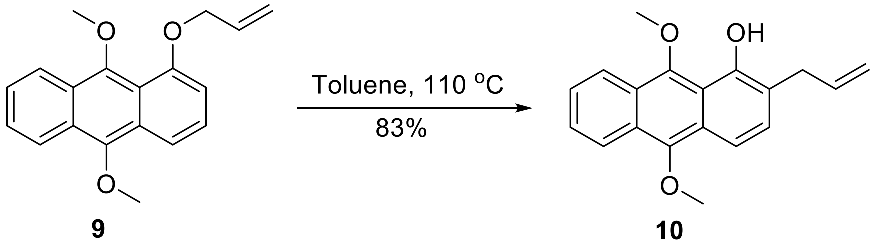

A solution of **9** (294 mg, 1.0 mmol, 1.0 equiv.) in Toluene (9.0 mL) was heated to 110 °C by heating mantle. After stirring at this temperature for 12h the mixture was cooled down to room temperature. The mixture was concentrated under reduced pressure. The crude residue was purified by flash chromatography (Hexane /ethyl acetate = 10/1 to 4:1), to afford **10**.

2-allyl-9,10-dimethoxyanthracen-1-ol (**10**) (244 mg, white amorphous solid, yield = 83%). hexane/ethyl acetate = 8/1, rf = 0.2. ^{1}H NMR (500 MHz, $CDCl_3$-*d*) δ 10.02 (s, 1H), 7.90 (d, *J* = 8.9 Hz, 1H), 7.78 (dd, *J* = 8.7, 0.9 Hz, 1H), 7.38 (dd, *J* = 8.8, 7.5 Hz, 1H), 7.32 (d, *J* = 8.9 Hz, 1H), 6.83 – 6.72 (m, 1H), 6.14 (dd, *J* = 17.0, 10.1 Hz, 1H), 5.19 (dd, *J* = 17.1, 1.8 Hz, 1H), 5.13 (dd, *J* = 10.1, 1.7 Hz, 1H), 4.10 (s, 3H), 4.09 (s, 3H), 4.02 (s, 3H), 3.63 (dt, *J* = 6.6, 1.5 Hz, 2H). ^{13}C NMR (126 MHz, $CDCl_3$-*d*) δ 156.5, 149.7, 149.4, 146.8, 136.9, 128.8, 127.0, 126.0, 125.5, 119.4, 118.0, 116.6, 115.4, 114.0, 114.0., 103.6, 63.9, 63.3, 56.1, 33.8. HRMS (ESI+): calculated for $C_{19}H_{19}O_3$ $[M+H]^+$ 295.1329, found 295.1341.

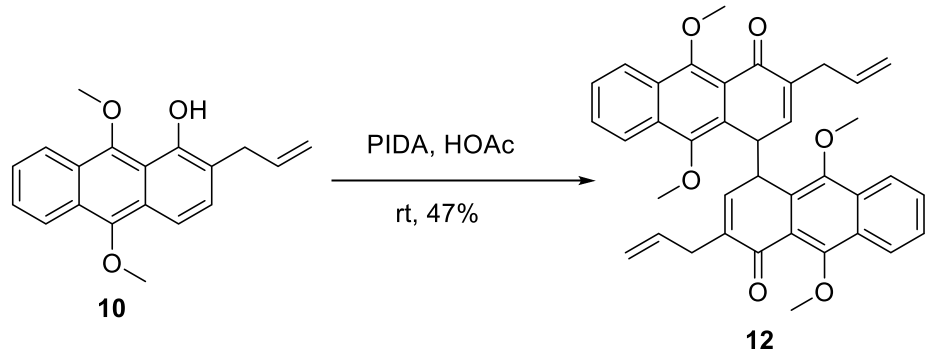

10 **12**

A Schlenk tube was charged with **10** (1.0 mmol, 1.0 equiv.) and PIDA (1.2 mmol, 1.2 equiv.) HOAc(0.6 mmol, 0.6 equiv.) in 20 mL toluene. After stirring at room temperature for 0.5 hours, the mixture was filtered through a pad of Celite, and the filtrate was concentrated under reduced pressure. The crude residue was purified by silica gel column chromatography (Hexane /ethyl acetate = 6/1) to yield enone dimers **12**.

3,3'-diallyl-8,8',9,9',10,10'-hexamethoxy-[1,1'-bianthracene]-4,4'(1H,1'H)-dione (**12**) (got 141 mg from 300 mg **8**, white amorphous solid, yield = 47%) ^{1}H NMR (500 MHz, Acetone-d_6) δ 8.39 (d, *J* = 8.4 Hz, 1H), 8.28 (d, *J* = 8.4 Hz, 1H), 7.82 – 7.74 (m, 1H), 7.72 – 7.64 (m, 1H), 5.94 – 5.85 (m, 1H), 5.61 (ddt, *J* = 17.0, 10.0, 6.9 Hz, 1H), 5.16 (m, 1H), 4.92 (d, *J* = 18.5 Hz, 1H), 4.85 (d, *J* = 11.0 Hz, 1H), 4.27 (s, 3H), 4.06 (s, 3H), 2.96 (d, *J* = 6.9 Hz, 2H). ^{13}C NMR (126 MHz, Acetone-d_6) δ 184.2, 155.4, 150.2, 143.4, 137.5, 136.1, 131.6, 130.5, 130.3, 129.8, 127.7, 125.5, 123.4, 123.2, 117.4, 63.7, 63.3, 42.5, 34.6. HRMS (ESI+): calculated for $C_{38}H_{35}O_6$ $[M+H]^+$ 587.2428, found 587.2390

3. Oxidation conditions screening

Table 1. Dearomative Dimerization Substrate Scope.

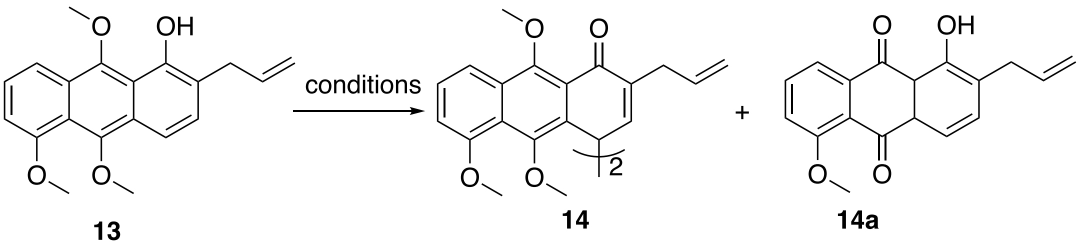

13 **14** **14a**

Entry	Reagent	Sol.	Time	Product and Yeild	
1	$K_3Fe(CN)_6$	MeOH/EtN_3	8 h	N. R.	
2	$AgClO_4$	Tol.	0.5 h	**14** 0%	**14a** 43%
3	Ag_2CO_3	Tol.	8 h	**14** 72%	
4	IBX	DMSO	> 8 h	**14** trace	
5	PIDA	Tol.	0.5 h	**14** 47 %	+ **14a** 13%
6	$Mn(AcO)_3$	Tol.	> 8 h	**14** trace	
7	MnO_2	Tol.	2 h	**14** 63%	
8	Air	Tol.	> 8 h	N. R.	
9	PbO_2	Tol.	1.5 h	**14** 75%	
10	PbO_2	nitromethane	2.0 h	**14** 95%	

4. Preparation of anthracenol monomers

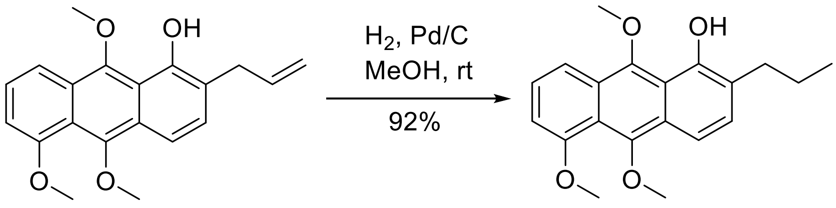

S1 **S2**

Substrate **S1** was known compounds and prepared according to the procedures reported in literature.[6)]

A Schlenk tube was charged with **S1** (324 mg, 1.0 mmol, 1.0 equiv.) and Pd/C (107 mg, 0.1 equiv., 10% on carbon) under argon atmosphere. The tube was subsequently filled with hydrogen. After stirring at 25 °C for 2 h, the mixture was filtered through a pad of Celite, and the filtrate was concentrated under reduced pressure. The crude residue was purified by silica gel column chromatography (Hexane /ethyl acetate = 20/1) to yield **S2**.

5,9,10-trimethoxy-2-propylanthracen-1-ol (**S2**) (300 mg, white amorphous solid, yield = 92%). Hexane/ethyl acetate = 20/1, rf = 0.25.^{1}H NMR (500 MHz, $CDCl_3$-*d*) δ 9.96 (s, 1H), 7.87 (d, *J* = 8.8 Hz, 1H), 7.77 (d, *J* = 8.8 Hz, 1H), 7.41 – 7.35 (m, 1H), 7.32 (d, *J* = 8.9 Hz, 1H), 6.77 (d, *J* = 7.5 Hz, 1H), 4.09 (s, 3H), 4.09 (s, 3H), 4.02 (s, 3H), 2.87 – 2.78 (m, 2H), 1.77 (h, *J* = 7.4 Hz, 2H), 1.06 (t, *J* = 7.3 Hz, 3H). ^{13}C NMR (126 MHz, $CDCl_3$-*d*) δ 156.5, 149.7, 149.4, 146.7, 129.2, 126.8, 125.9, 125.4, 122.2, 117.8, 116.6, 113.9, 113.7, 103.5, 63.8, 63.2, 56.0, 31.8, 23.2, 14.3. HRMS (ESI+): calculated for $C_{20}H_{23}O_4$ $[M+H]^+$ 327.1591, found 327.1616.

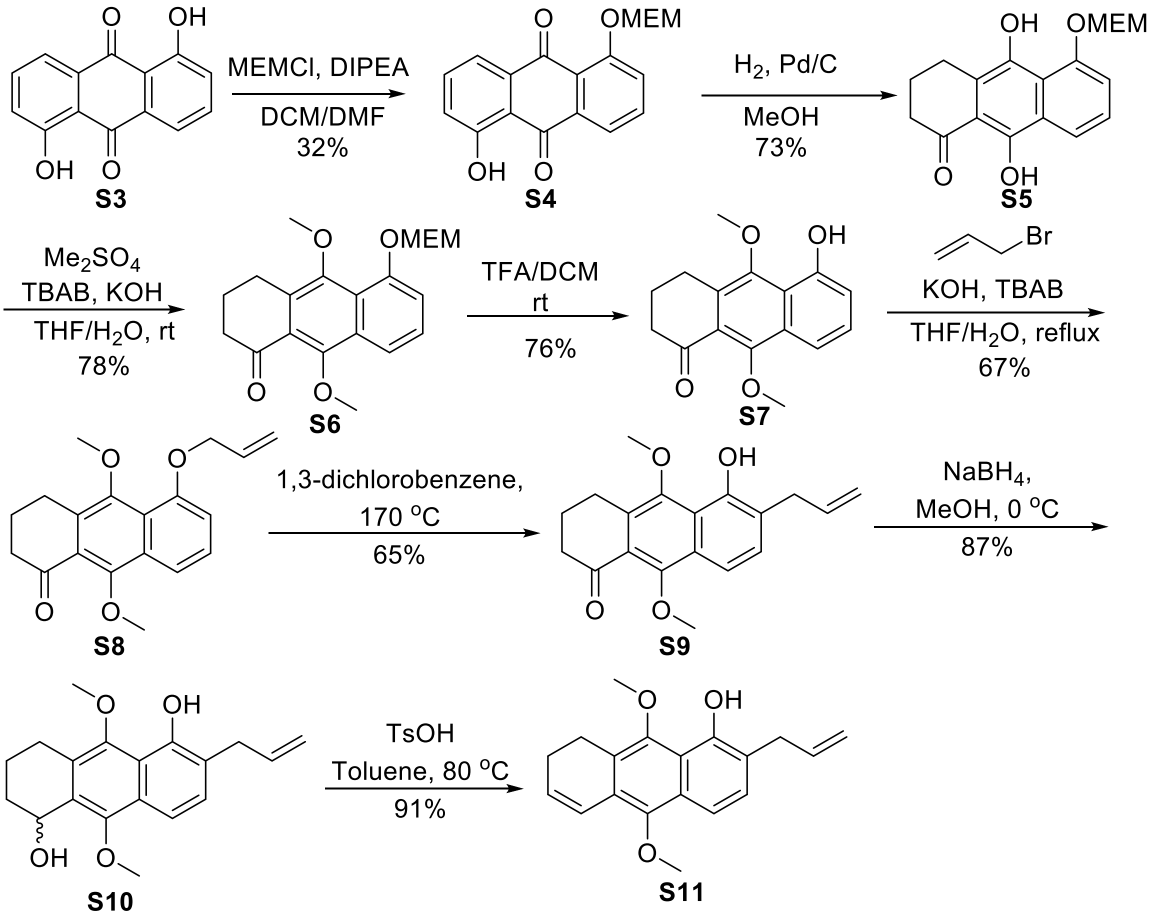

Scheme S1 Preparation of anthracenol derivative monomer **S11**

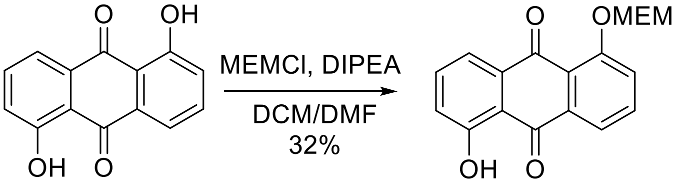

A solution of 1,5-Dihydroxyanthraquinone (commercially available) (1.2g, 0.5 mmol, 1.0 equiv.) in CH_2Cl_2 (25 mL) and DMF (5 mL) mixture was added MEMCl (628 μL, 0.55

mmol, 1.1 equiv.) followed by addition of DIPEA (872 μL, 0.6 mmol, 1.2 equiv.). After stirring for 12h, Then water (30 mL) was added. The organic layer was separated, and the aqueous layer was extracted with CH_2Cl_2 (4 × 10 mL). The combined organic layers were washed with saturated aqueous NaCl (2 × 5 mL), dried ($MgSO_4$), and concentrated under reduced pressure. The crude residue was purified by flash chromatography (Hexane /ethyl acetate = 5/1) to afford **S4**.

1-hydroxy-5-((2-methoxyethoxy)methoxy)anthracene-9,10-dione (**S4**) (525 mg, white amorphous solid, yield = 32%). hexane/ethyl acetate = 5/1, rf = 0.25. ^{1}H NMR (500 MHz, Chloroform-*d*) δ 12.51 (s, 1H), 8.08 (dd, *J* = 7.6, 1.2 Hz, 1H), 7.79 (dd, *J* = 7.6, 1.2 Hz, 1H), 7.76 – 7.70 (m, 1H), 7.68 (dt, *J* = 8.4, 1.2 Hz, 1H), 7.29 (d, *J* = 1.1 Hz, 1H), 5.52 (s, 3H), 4.06 – 3.84 (m, 3H), 3.68 – 3.50 (m, 3H), 3.39 (s, 3H). ^{13}C NMR (126 MHz, Chloroform-*d*) δ 188.4, 181.7, 162.0, 158.0, 136.9, 135.2, 135.0, 134.9, 123.1, 123.1, 122.52, 120.9, 119.3, 115.6, 94.1, 71.4, 68.3, 59.0. HRMS (ESI+): calculated for $C_{18}H_{17}O_6$ $[M+H]^+$ 329.1020, found 329.1033.

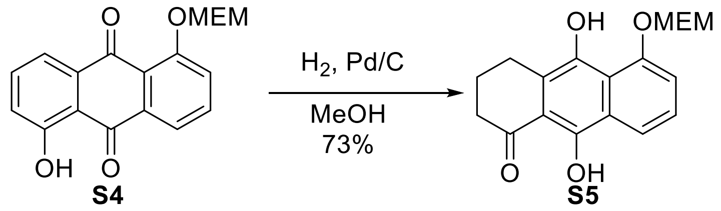

A Schlenk tube was charged with **S4** (142 mg, 0.43 mmol, 1.0 equiv.) and Pd/C (45 mg, 0.1 equiv., 10% on carbon) under argon atmosphere. The tube was subsequently filled with hydrogen. After stirring at 25 ^{o}C for 2 h, the mixture was filtered through a pad of Celite, and the filtrate was concentrated under reduced pressure. The crude residue was purified by silica gel column chromatography (Hexane /ethyl acetate = 5/1) to yield **S5**.

9,10-dihydroxy-5-((2-methoxyethoxy)methoxy)-3,4-dihydroanthracen-1(2*H*)-one (**S5**) (105 mg, white amorphous solid, yield = 73%). ^{1}H NMR (500 MHz, Chloroform-*d*) δ 13.51 (s, 1H), 9.02 (s, 1H), 8.11 (dd, *J* = 8.2, 1.3 Hz, 1H), 7.40 – 7.22 (m, 2H), 5.54 (s, 2H), 4.00 – 3.82 (m, 2H), 3.66 – 3.55 (m, 2H), 3.40 (s, 3H), 3.01 (dd, *J* = 6.8, 5.7 Hz, 2H), 2.74 (dd, *J* = 7.2, 5.8 Hz, 2H), 2.12 (tt, *J* = 7.1, 5.7 Hz, 2H). ^{13}C NMR (126 MHz, Chloroform-*d*) δ 205.5, 154.9, 152.7, 141.0, 125.6, 124.9, 119.4, 119.4, 119.1, 112.7, 111.8, 94.9, 71.5, 68.7, 59.0, 38.9, 22.7, 22.2. HRMS (ESI+): calculated for $C_{18}H_{21}O_6$ $[M+H]^+$ 333.1333, found 333.1357.

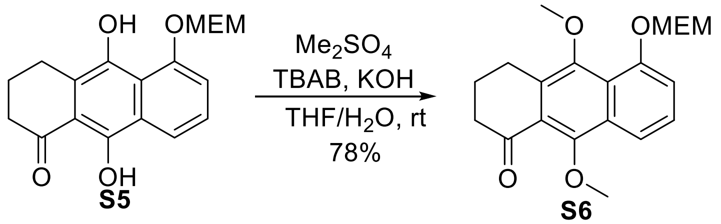

S5 S6

KOH (195 mg, 3.47 mmol, 10.0 equiv.) was added into a THF (5 mL) and water (2.5 mL) solution of **S5** (105 mg, 0.34 mmol, 1.0 equiv.) and dimethyl sulfate (132 µL, 1.39 mmol, 4.0 equiv.). The mixture was stirred at room temperature for 2h. Then water (5 mL) and ethyl acetate (10 mL) were added. The organic layer was separated, and the aqueous layer was extracted with ethyl acetate (3 × 8 mL). The combined organic layers were washed with saturated aqueous NaCl (2 × 5 mL), dried ($MgSO_4$), and concentrated under reduced pressure. The crude residue was purified by flash chromatography (Hexane /ethyl acetate = 3/1) to afford **S6**.

9,10-dimethoxy-5-((2-methoxyethoxy)methoxy)-3,4-dihydroanthracen-1(2*H*)-one (**S6**) (95 mg, white amorphous solid, yield = 78%). hexane/ethyl acetate = 3/1, rf = 0.2. ^{1}H NMR (500 MHz, $CDCl_3$-*d*) δ 8.02 (dt, *J* = 8.4, 1.0 Hz, 1H), 7.42 – 7.37 (m, 1H), 7.31 (dt, *J* = 7.7, 1.0 Hz, 1H), 5.47 – 5.37 (m, 2H), 4.01 – 3.93 (m, 5H), 3.81 (d, *J* = 0.8 Hz, 3H), 3.64 – 3.60 (m, 2H), 3.40 (d, *J* = 0.8 Hz, 3H), 3.12 (t, *J* = 6.1 Hz, 2H), 2.70 (t, *J* = 6.6 Hz, 2H), 2.10 (p, *J* = 6.4 Hz, 2H). ^{13}C NMR (126 MHz, $CDCl_3$-*d*) δ 197.8, 154.5, 152.8, 148.3, 132.1, 131.0, 126.1, 123.9, 122.5, 118.7, 114.8, 95.3, 71.6, 68.1, 62.9, 61.5, 59.1, 40.9, 23.8, 22.3. HRMS (ESI+): calculated for $C_{20}H_{25}O_6$ $[M+H]^+$ 361.1646, found 361.1679.

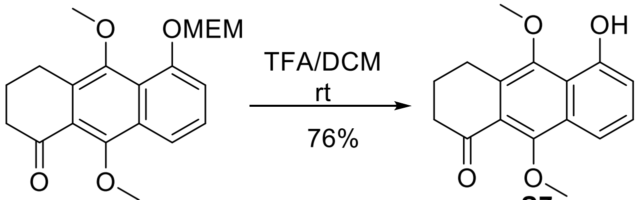

S6 S7

A solution of **S6** (92 mg, 0.25 mmol, 1.0 equiv.) in CH_2Cl_2 (2.5 mL) was added TFA (98 µL, 1.27 mmol, 5.0 equiv.). After stirring at room temperature for 1h the solvent was blown out by air flow. The crude residue was purified by flash chromatography (Hexane /ethyl acetate = 2.5/1) to afford **S7**.

5-hydroxy-9,10-dimethoxy-3,4-dihydroanthracen-1(2*H*)-one (**S7**) (54 mg, white amorphous solid, yield = 76%). Hexane/ethyl acetate = 2.5/1, rf = 0.4. ^{1}H NMR (500 MHz, $CDCl_3$-*d*) δ 9.53 (s, 1H), 7.82 (dd, *J* = 8.5, 1.1 Hz, 1H), 7.40 (dd, *J* = 8.4, 7.6 Hz, 1H), 7.03 (dd, *J* = 7.6, 1.1 Hz, 1H), 3.97 (s, 3H), 3.92 (s, 3H), 3.09 – 3.05 (m, 2H), 2.75 – 2.70 (m, 2H), 2.14 – 2.04 (m, 2H). ^{13}C NMR (126 MHz, $CDCl_3$-*d*) δ 197.2, 155.7, 153.2, 147.9, 130.3, 129.4, 127.5, 122.2, 120.0, 115.8, 113.6, 62.9, 62.3, 40.8, 23.8, 22.2. HRMS (ESI+): calculated for $C_{16}H_{17}O_4$ $[M+H]^+$ 273.1121, found 273.1127.

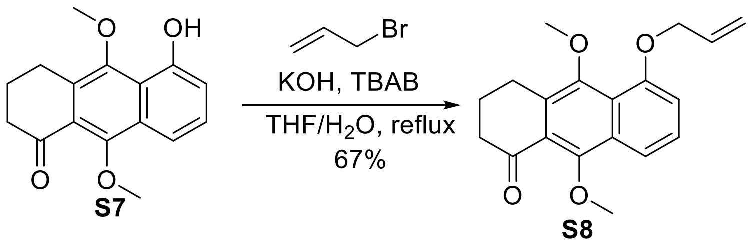

KOH (34 mg, 0.6 mmol, 3.0 equiv.) was added into a THF (5 mL) and water (0.5 mL) mixture of **S7** (52 mg, 0.2 mmol, 1.0 equiv.), TBAB (20 mg, 0.06 mmol, 0.3 equiv.) and allyl bromide (35 μL, 0.4 mmol, 2.0 equiv.). After stirring at room temperature for 3h. Saturated NH_4Cl (10 mL) and ethyl acetate (8 mL) were added. The organic layer was separated, and the aqueous layer was extracted with ethyl acetate (3 × 8 mL). The combined organic layers were washed with saturated aqueous NaCl (2 × 5 mL), dried ($MgSO_4$), and concentrated under reduced pressure. The crude residue was purified by flash chromatography (Hexane /ethyl acetate = 2/1) to afford **S8**.

5-(allyloxy)-9,10-dimethoxy-3,4-dihydroanthracen-1(2*H*)-one (**S8**) (42 mg, white amorphous solid, yield = 67%). Hexane/ethyl acetate = 2/1, rf = 0.3. ^{1}H NMR (500 MHz, $CDCl_3$-*d*) δ 7.94 (dd, *J* = 8.5, 1.0 Hz, 1H), 7.39 (dd, *J* = 8.5, 7.7 Hz, 1H), 6.99 (dd, *J* = 7.7, 1.0 Hz, 1H), 6.21 (ddt, *J* = 17.3, 10.6, 5.3 Hz, 1H), 5.55 (dq, *J* = 17.2, 1.6 Hz, 1H), 5.36 (dq, *J* = 10.5, 1.4 Hz, 1H), 4.69 (dt, *J* = 5.3, 1.5 Hz, 2H), 3.97 (s, 3H), 3.79 (s, 3H), 3.18 – 3.09 (m, 2H), 2.70 (dd, *J* = 7.2, 6.0 Hz, 2H), 2.15 – 2.04 (m, 2H). ^{13}C NMR (126 MHz, $CDCl_3$-*d*) δ 197.9, 154.5, 154.4, 148.7, 133.1, 132.0, 131.0, 126.0, 123.7, 122.7, 118.0, 117.3, 110.5, 70.3, 62.8, 61.6, 41.0, 23.8, 22.3. HRMS (ESI+): calculated for $C_{19}H_{21}O_4$ $[M+H]^+$ 313.1434, found 313.1413.

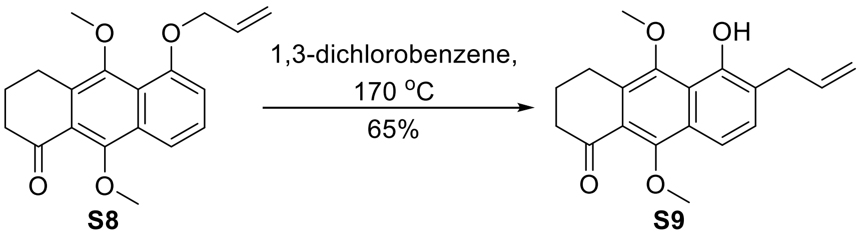

A solution of **S8** (35 mg, 0.11 mmol, 1.0 equiv.) in 1,3-dichlorobenzene (1.0 mL) was heated to 170 °C by heating mantle. After stirring at this temperature for 2h the mixture was cooled down to room temperature. The mixture was directly loaded on the silica gel column and purified by flash chromatography (Hexane /ethyl acetate = 2/1), to afford **S9**.

6-allyl-5-hydroxy-9,10-dimethoxy-3,4-dihydroanthracen-1(2*H*)-one (**S9**) (23 mg, white amorphous solid, yield = 65%). Hexane/ethyl acetate = 2/1, rf = 0.25. ^{1}H NMR (500 MHz, Chloroform-*d*) δ 9.77 (s, 1H), 7.78 (d, *J* = 8.5 Hz, 1H), 7.32 (d, *J* = 8.6 Hz, 1H), 6.06 (ddt, *J* = 16.6, 10.1, 6.5 Hz, 1H), 5.18 – 5.04 (m, 2H), 3.96 (s, 3H), 3.91 (s, 3H), 3.56 (dt, *J* = 6.5, 1.5 Hz, 2H), 3.06 (dd, *J* = 6.8, 5.3 Hz, 2H), 2.79 – 2.64 (m, 2H), 2.18 – 1.98 (m, 2H).

^{13}C NMR (126 MHz, Chloroform-*d*) δ 197.2, 155.8, 150.0, 147.7, 136.3, 129.4, 129.3, 129.0, 124.7, 121.6, 119.8, 115.7, 115.5, 62.8, 62.3, 40.8, 34.0, 23.8, 22.3. HRMS (ESI+): calculated for $C_{19}H_{21}O_4$ $[M+H]^+$ 313.1434, found 313.1410.

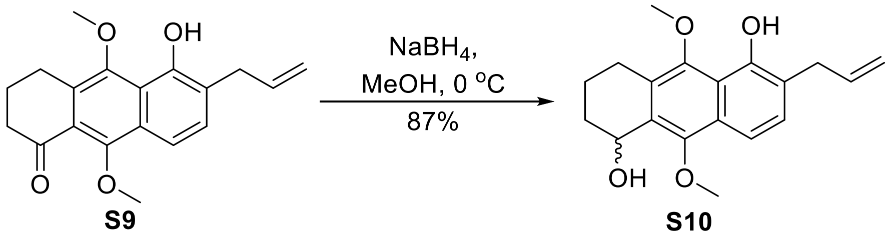

A solution of **S9** (80 mg, 0.26 mmol, 1.0 equiv.) in MeOH (1.3 mL) at 0 °C was added $NaBH_4$ (10 mg, 0.26 mmol, 1.0 equiv.). After stirring at 0 °C for 1h, the reaction was quenched by adding saturated NH_4Cl (2 mL). Then water (4 mL) and ethyl acetate (5 mL) were added. The organic layer was separated, and the aqueous layer was extracted with ethyl acetate (3 × 5 mL). The combined organic layers were washed with saturated aqueous NaCl (2 × 5 mL), dried ($MgSO_4$), and concentrated under reduced pressure. The crude residue was purified by flash chromatography (Hexane /ethyl acetate = 3/1) to afford **S10**.

6-allyl-9,10-dimethoxy-1,2,3,4-tetrahydroanthracene-1,5-diol (**S10**) (70 mg, white amorphous solid, yield = 87%). Hexane/ethyl acetate = 3/1, rf = 0.3. ^{1}H NMR (500 MHz, Chloroform-*d*) δ 9.84 (s, 1H), 7.47 (d, *J* = 8.5 Hz, 1H), 7.29 – 7.23 (m, 1H), 6.08 (ddt, *J* = 16.6, 10.1, 6.5 Hz, 1H), 5.22 (t, *J* = 4.9 Hz, 1H), 5.14 – 5.04 (m, 2H), 3.98 (s, 3H), 3.89 (s, 3H), 3.55 (ddt, *J* = 7.0, 5.6, 1.6 Hz, 2H), 3.30 (s, 1H), 3.06 – 2.97 (m, 1H), 2.77 (ddd, *J* = 16.9, 9.0, 5.3 Hz, 1H), 2.09 – 1.92 (m, 3H), 1.83 – 1.74 (m, 1H). ^{13}C NMR (126 MHz, Chloroform-*d*) δ 151.6, 150.3, 149.0, 136.9, 129.1, 128.8, 127.6, 126.1, 121.2, 117.4, 115.3, 112.7, 64.0, 62.0, 61.8, 33.8, 30.9, 23.9, 18.3. HRMS (ESI+): calculated for $C_{19}H_{23}O_4$ $[M+H]^+$ 315.1591, found 315.1613.

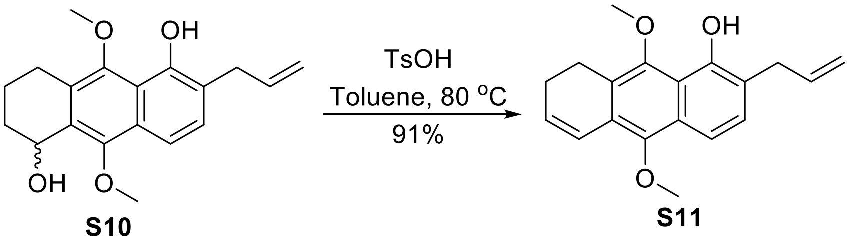

A solution of **S10** (68 mg, 0.22 mmol, 1.0 equiv.) in toluene (2.2 mL) was added TsOH (75 mg, 0.43 mmol, 2.0 equiv.). After stirring at 80 °C for 1h the mixture was cooled down to room temperature. Saturated NH_4Cl (5 mL) and ethyl acetate (4 mL) were added. The organic layer was separated, and the aqueous layer was extracted with ethyl acetate (3 × 3 mL). The combined organic layers were washed with saturated aqueous NaCl (2 × 5 mL), dried ($MgSO_4$), and concentrated under reduced pressure. The crude residue was purified by flash chromatography (Hexane /ethyl acetate = 20/1) to afford **S11**.

2-allyl-9,10-dimethoxy-7,8-dihydroanthracen-1-ol (**S11**) (59 mg, white amorphous solid, yield = 91%). Hexane/ethyl acetate = 20/1, rf = 0.25. ^{1}H NMR (500 MHz, $CDCl_3$-*d*) δ 9.84

(d, J = 0.5 Hz, 1H), 7.54 (d, J = 8.5 Hz, 1H), 7.31 – 7.22 (m, 1H), 6.95 (dt, J = 9.8, 1.8 Hz, 1H), 6.20 (dt, J = 9.8, 4.5 Hz, 1H), 6.10 (ddt, J = 17.1, 10.1, 6.5 Hz, 1H), 5.18 – 5.07 (m, 2H), 3.88 (s, 3H), 3.87 (s, 3H), 3.56 (dt, J = 6.5, 1.6 Hz, 2H), 2.95 (t, J = 7.6 Hz, 2H), 2.46 – 2.25 (m, 2H). ^{13}C NMR (126 MHz, Chloroform-*d*) δ 150.5, 148.0, 148.0, 137.0, 129.7, 128.8, 128.3, 124.1, 123.3, 122.1, 121.3, 116.8, 115.2, 113.2, 62.3, 62.2, 33.9, 22.8, 21.0.

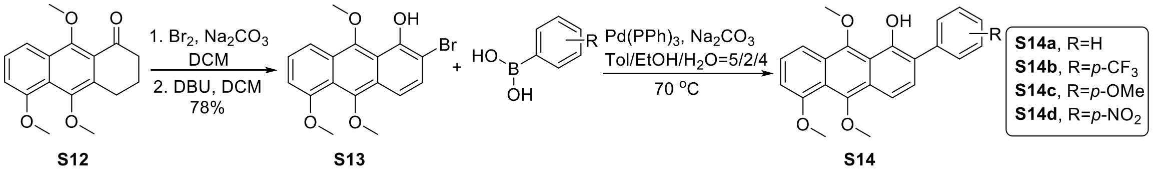

Scheme S2. Preparation of α-aryl substituted anthracenol monomer **S14a-S14d**.

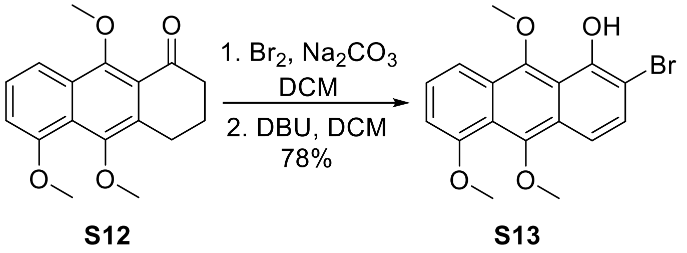

HRMS (ESI+): calculated for $C_{19}H_{21}O_3$ $[M+H]^+$ 297.1485, found 297.1556.

Substrate **S12** was known compounds and prepared according to the procedures reported in literature.[7)]

A solution of **S12** (300 mg, 1.05 mmol, 1.0 equiv.) and Na_2CO_3 (222 mg, 2.62 mmol, 2.5 equiv.) in CH_2Cl_2 (4 mL) was added Br_2 (135 μL, 2.62 mmol, 2.5 equiv.) in one portion. After stirring at room temperature for 30 min, DBU (176 μL, 1.16 mmol, 1.1 equiv.) was added. Then the resulting mixture was allowed stirring at room temperature for additional 45 min. The reaction was quenched by saturated sodium thiosulfate (4 mL). Then water (5 mL) and CH_2Cl_2 (4 mL) were added. The organic layer was separated, and the aqueous layer was extracted with CH_2Cl_2 (4 × 5 mL). The combined organic layers were washed with saturated aqueous NaCl (2 × 5 mL), dried ($MgSO_4$), and concentrated under reduced pressure. The crude residue was purified by flash chromatography (Hexane /ethyl acetate = 7/1) to afford **S13**.

2-bromo-5,9,10-trimethoxyanthracen-1-ol (**S13**) (297 mg, white amorphous solid, yield = 78%). Hexane/ethyl acetate = 7/1, rf = 0.3. ^{1}H NMR (500 MHz, Chloroform-*d*) δ 10.47 (s, 1H), 7.77 (d, J = 9.3 Hz, 1H), 7.74 (dd, J = 8.8, 0.9 Hz, 1H), 7.49 (d, J = 9.3 Hz, 1H), 7.40 (dd, J = 8.8, 7.5 Hz, 1H), 6.80 (d, J = 7.5 Hz, 1H), 4.09 (s, 3H), 4.07 (s, 3H), 3.98 (s, 3H). ^{13}C NMR (126 MHz, Chloroform-*d*) δ 156.5, 150.1, 149.2, 146.3, 130.0, 126.6, 126.4, 126.3, 118.7, 116.7, 115.5, 113.9, 104.2, 103.4, 64.2, 63.5, 56.2. HRMS (ESI+): calculated for $C_{17}H_{16}BrO_4$ $[M+H]^+$ 363.0226, found 363.0273.

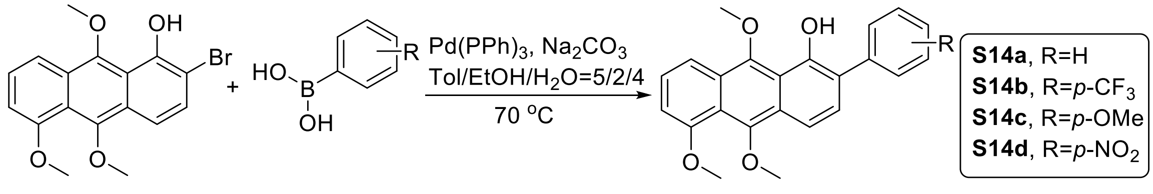

Under argon atmosphere, a solution of **S13** (0.41 mmol, 1.0 equiv.), boronic acid (0.83 mmol, 2.0 equiv.), Na_2CO_3 (1.66 mmol, 4.0 equiv.) and Tetrakis(triphenylphosphine) palladium ($Pd(PPh)_3$) (0.041 mmol, 0.1 equiv.) in degassed mixture (Tol/EtOH/H_2O = 5/2/4, 6.5 mL) was heated to 70 °C by an oil bath. After stirring for 8h, the mixture was cooled down to room temperature, then water (5 mL) and ethyl acetate (5 mL) were added. The resulting mixture was filtered through a pad of Celite. The organic layer was separated, and the aqueous layer was extracted with ethyl acetate (3 × 8 mL). The combined organic layers were washed with saturated aqueous NaCl (2 × 5 mL), dried ($MgSO_4$), and concentrated under reduced pressure. The crude residue was purified by flash chromatography (Hexane /ethyl acetate = 7/1) to afford **S14a**-**S14d**.

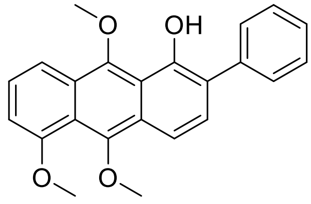

S14a

5,9,10-trimethoxy-2-phenylanthracen-1-ol (**S14a**) (124 mg, white amorphous solid, yield = 83%). Hexane/ethyl acetate = 7/1, rf = 0.25. ^{1}H NMR (500 MHz, $CDCl_3$-*d*) δ 10.33 (s, 1H), 7.99 (d, *J* = 9.0 Hz, 1H), 7.79 (t, *J* = 7.4 Hz, 3H), 7.52 (t, *J* = 8.6 Hz, 3H), 7.45 – 7.35 (m, 2H), 6.82 (d, *J* = 7.5 Hz, 1H), 4.12 (s, 3H), 4.11 (s, 3H), 4.05 (s, 3H). ^{13}C NMR (126 MHz, $CDCl_3$-*d*) δ 156.5, 149.7, 149.3, 147.4, 138.5, 129.7, 128.8, 128.2, 127.1, 126.8, 126.3, 125.7, 121.1, 118.5, 116.8, 114.3, 114.0, 103.9, 64.0, 63.4, 56.1. HRMS (ESI+): calculated for $C_{23}H_{21}O_4$ $[M+H]^+$ 361.1434, found 361.1449.

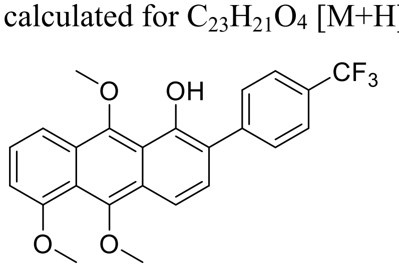

S14b

5,9,10-trimethoxy-2-(4-(trifluoromethyl)phenyl)anthracen-1-ol (**S14b**) (154 mg, white amorphous solid, yield = 88%). Hexane/ethyl acetate = 7/1, rf = 0.25. ^{1}H NMR (500 MHz, Chloroform-*d*) δ 10.46 (s, 1H), 8.01 (d, *J* = 9.0 Hz, 1H), 7.91 (d, *J* = 8.0 Hz, 2H), 7.81 (d, *J* = 8.8 Hz, 1H), 7.76 (d, *J* = 8.1 Hz, 2H), 7.49 (d, *J* = 8.9 Hz, 1H), 7.44 (dd,

J = 8.8, 7.5 Hz, 1H), 6.85 (d, *J* = 7.5 Hz, 1H), 4.13 (s, 3H), 4.12 (s, 3H), 4.05 (s, 3H). ^{13}C NMR (126 MHz, $CDCl_3$-*d*) δ 156.6, 149.8, 147.6, 142.2, 129.9, 128.8, 128.5, 128.1, 127.2, 126.4, 125.9, 125.5, 125.1, 125.1, 125.0, 123.3, 119.5, 118.8, 116.6, 114.6, 113.9, 104.1, 64.1, 63.4, 56.1. ^{19}F NMR (Chloroform-*d*) δ 63.4(s). HRMS (ESI+): calculated for $C_{24}H_{20}F_3O_4$ $[M+H]^+$ 429.1308, found 429.1398.

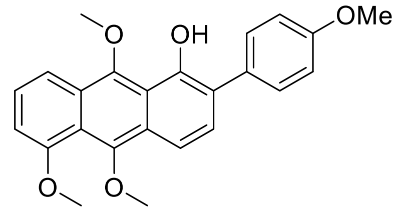

S14c

5,9,10-trimethoxy-2-(4-methoxyphenyl)anthracen-1-ol (**S14c**) (144 mg, white amorphous solid, yield = 89%). Hexane/ethyl acetate = 7/1, rf = 0.25. ^{1}H NMR (400 MHz, $CDCl_3$-*d*) δ 10.30 (s, 1H), 7.97 (d, *J* = 9.0 Hz, 1H), 7.78 (d, *J* = 8.8 Hz, 1H), 7.73 (d, *J* = 8.1 Hz, 2H), 7.50 (d, *J* = 9.0 Hz, 1H), 7.39 (t, *J* = 8.1 Hz, 1H), 7.05 (d, *J* = 8.2 Hz, 2H), 6.80 (d, *J* = 7.5 Hz, 1H), 4.10 (s, 3H), 4.09 (s, 3H), 4.04 (s, 3H), 3.89 (s, 3H). ^{13}C NMR (101 MHz, $CDCl_3$-*d*) δ 158.4, 156.4, 149.6, 149.0, 147.2, 130.8, 130.7, 128.8, 128.1, 126.9, 126.2, 125.6, 120.7, 118.3, 116.8, 116.7, 114.2, 113.9, 113.6, 103.7, 64.0, 63.3, 56.1, 55.3. HRMS (ESI+): calculated for $C_{24}H_{23}O_5$ $[M+H]^+$ 391.1540, found 391.1590.

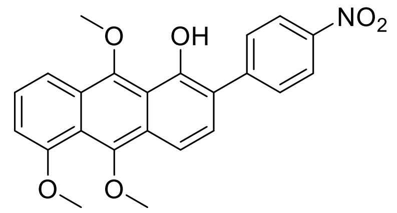

S14d

5,9,10-trimethoxy-2-(4-nitrophenyl)anthracen-1-ol (**S14d**) (131 mg, white amorphous solid, yield = 78%). Hexane/ethyl acetate = 8/1, rf = 0.25. ^{1}H NMR (400 MHz, $CDCl_3$-*d*) δ 8.09 (d, *J* = 8.3 Hz, 2H), 8.02 (d, *J* = 8.5 Hz, 1H), 7.56 (t, *J* = 8.1 Hz, 1H), 7.31 (d, *J* = 8.3 Hz, 2H), 7.12 (d, *J* = 7.8 Hz, 1H), 6.43 – 6.42 (m, 1H), 5.63 – 5.62 (m, 1H), 4.20 (s, 3H), 4.14 (s, 3H), 4.00 (s, 3H). ^{13}C NMR (101 MHz, $CDCl_3$-*d*) δ 182.7, 155.6, 154.6, 149.8, 147.2, 142.4, 142.3, 142.0, 131.8, 129.4, 128.4, 127.3, 123.1, 123.1, 122.8, 117.0, 109.0, 63.3, 63.3, 56.2, 41.4. HRMS (ESI+): calculated for $C_{23}H_{20}NO_6$ $[M+H]^+$ 406.1285, not found.

5. Procedures for Dearomatized Enone Dimers

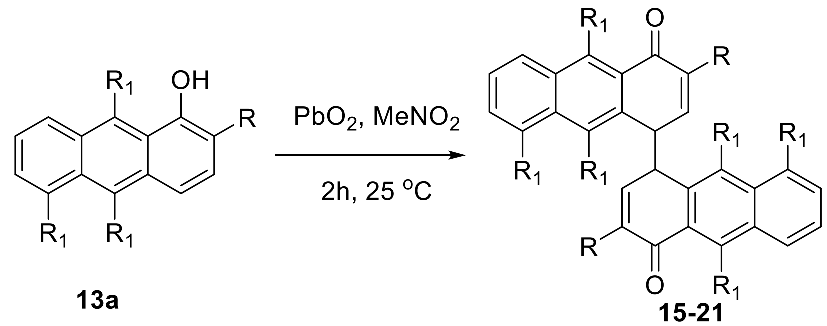

A Schlenk tube was charged with **13a** (0.1 mmol, 1.0 equiv.) and PbO_2 (0.12 mmol, 1.2 equiv) in 2 mL nitromethane. After stirring at 25 °C for 2 h under the room temperature and pressure, the mixture was filtered through a pad of Celite, and the filtrate was concentrated under reduced pressure. The crude residue was purified by silica gel chromatography (Hexane /ethyl acetate = 10/1 to 4/1) to yield enone dimers **15-21**.

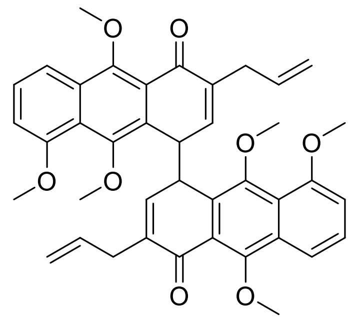

15

A Schlenk tube was charged with **S1** (1 mmol, 1.0 equiv.) and PbO_2 (1.2 mmol, 1.2 equiv) in 20 mL nitromethane. After stirring at 25 °C for 2 h under the room temperature and pressure, the mixture was filtered through a pad of Celite, and the filtrate was concentrated under reduced pressure. The crude residue was purified by silica gel chromatography (Hexane /ethyl acetate = 10/1 to 4/1) to yield enone dimers **15**

3,3'-diallyl-8,8',9,9',10,10'-hexamethoxy-[1,1'-bianthracene]-4,4'(1H,1'H)-dione (**15**) (got 311 mg from 324 mg **S1**, white amorphous solid, yield = 96%. Solvent for crystal growth: Hexane/DCM=1:1) ^{1}H NMR (400 MHz, Chloroform-*d*) δ 8.02 (d, *J* = 8.5 Hz, 1H), 7.50 (t, *J* = 8.1 Hz, 1H), 7.06 (d, *J* = 7.7 Hz, 1H), 5.86 (s, 1H), 5.57 (td, *J* = 17.0, 6.9 Hz, 1H), 5.29 (s, 1H), 4.89 (d, *J* = 17.2 Hz, 1H), 4.85 (d, *J* = 10.7 Hz, 1H), 4.13 (s, 3H), 4.11 (s, 3H), 4.03 (s, 3H), 3.02 (dd, *J* = 16.2, 6.7 Hz, 1H), 2.93 (dd, *J* = 16.3, 6.8 Hz, 1H). ^{13}C NMR (101 MHz, Chloroform-*d*) δ 184.2, 155.6, 153.9, 149.7, 142.3, 138.1, 134.9, 131.7, 130.5,

126.7, 123.1, 122.9, 117.0, 117.0, 108.5, 63.2, 63.2, 56.3, 41.2, 33.8. HRMS (ESI+): calculated for $C_{40}H_{38}NaO_8$ $[M+Na]^+$ 669.2459, found 669.2548.

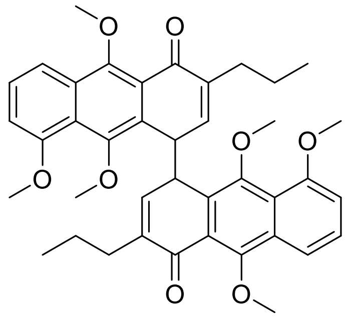

16

8,8',9,9',10,10'-hexamethoxy-3,3'-dipropyl-[1,1'-bianthracene]-4,4'(1H,1'H)-dione (**16**) (got 29.3 mg from 33 mg **S2**, white amorphous solid, yield = 89%) ^{1}H NMR (500 MHz, Acetone-d_6) δ 7.96 (d, J = 8.5 Hz, 1H), 7.56 (t, J = 8.1 Hz, 1H), 7.22 (d, J = 7.7 Hz, 1H), 5.90 – 5.86 (m, 1H),5.23 – 5.22 (m, 1H), 4.12 (s, 3H), 4.12 (s, 3H), 3.99 (s, 3H), 2.28 – 2.22 (m, 2H), 1.27 – 1.22 (m, 2H), 0.78 (t, J = 7.3 Hz, 3H). ^{13}C NMR (126 MHz, Acetone) δ 184.7, 156.9, 154.2, 150.7, 144.7, 137.8, 132.5, 131.3, 127.8, 124.1, 123.7, 117.2, 109.7, 63.3, 63.3, 56.7, 42.0, 32.8, 21.9, 14.3. $C_{40}H_{42}NaO_8$ $[M+Na]^+$ 673.2772, found 673.2784.

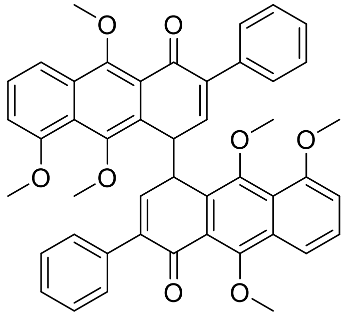

17

8,8',9,9',10,10'-hexamethoxy-3,3'-diphenyl-[1,1'-bianthracene]-4,4'(1*H*,1'*H*)-dione (**17**) (got 33.4 mg from 36 mg **S14a**, white amorphous solid, yield = 93%) ^{1}H NMR (500 MHz, Chloroform-d) δ 8.03 (d, J = 8.5 Hz, 1H), 7.52 (t, J = 8.1 Hz, 1H), 7.21 – 7.18 (m, 3H), 7.13 (dd, J = 7.5, 2.0 Hz, 2H), 7.08 (d, J = 7.7 Hz, 1H), 6.28 – 6.23 (m, 1H), 5.61 – 5.55 (m, 1H), 4.21 (s, 3H), 4.12 (s, 3H), 4.01 (s, 3H). ^{13}C NMR (126 MHz, Chloroform-d) δ 183.4, 155.6, 154.6, 149.8, 144.2, 140.4, 136.4, 132.0, 129.9, 128.6, 128.0, 127.7, 126.9,

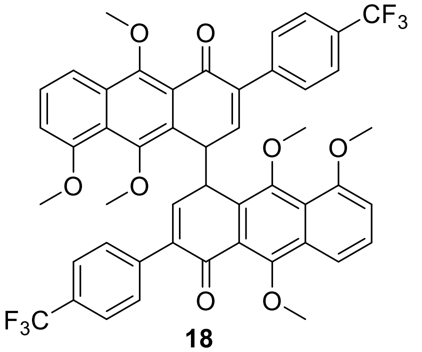

18

123.5, 123.0, 117.1, 108.7, 63.4, 63.4, 56.4, 41.3. HRMS (ESI+): calculated for $C_{46}H_{38}NaO_8$ $[M+Na]^+$ 741.2459, found 741.2604.

8,8',9,9',10,10'-hexamethoxy-3,3'-bis(4-(trifluoromethyl)phenyl)-[1,1'-bianthracene]-4,4'(1*H*,1'*H*)-dione (**18**) (got 38.2 mg from 43 mg **S14b**, yield = 89%) ^{1}H NMR (500 MHz, Chloroform-*d*) δ 8.03 (dd, *J* = 8.5, 1.0 Hz, 1H), 7.54 (t, *J* = 8.1 Hz, 1H), 7.48 (d, *J* = 8.2 Hz, 2H), 7.24 (s, 2H), 7.10 (d, *J* = 7.7 Hz, 1H), 6.32 (dd, *J* = 3.4, 1.3 Hz, 1H), 5.64 – 5.58 (m, 1H). ^{13}C NMR (126 MHz, $CDCl_3$) δ 183.1, 155.7, 154.7, 149.9, 143.1, 141.5, 139.7, 132.0, 130.0, 129.7, 129.2, 128.9, 127.2, 125.0 (q, *J* = 3.8 Hz), 123.1, 123.1, 117.2, 109.0, 63.4, 63.4, 56.4, 41.4. ^{19}F NMR (Chloroform-*d*) δ 63.4(s). HRMS (ESI+): calculated for $C_{48}H_{36}F_6NaO_8$ $[M+Na]^+$ 877.2207, found 877.2374.

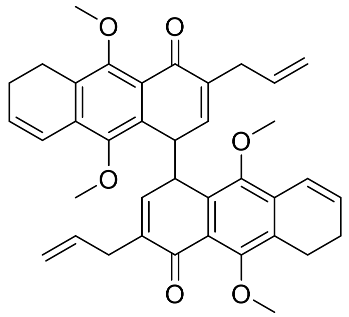

19

3,3'-diallyl-9,9',10,10'-tetramethoxy-5,5',6,6'-tetrahydro-[1,1'-bianthracene]-4,4'(1*H*,1'*H*)-dione (**19**) (got 28.4 mg from 30 mg **S11**, white amorphous solid, yield = 95%. Solvent for crystal growth: Hexane/DCM=1:1) ^{1}H NMR (500 MHz, Chloroform-*d*) δ 6.84 (ddd, *J* = 9.8, 2.2, 1.5 Hz, 1H), 6.37 – 6.32 (m, 1H), 5.85 (dd, *J* = 3.2, 1.3 Hz, 1H), 5.60 (ddt, *J* = 17.0, 10.1, 6.9 Hz, 1H), 4.95 – 4.88 (m, 2H), 4.79 – 4.76 (m, 1H), 3.93 (s, 3H), 3.81 (s, 3H), 3.05 (dt, *J* = 16.1, 7.1 Hz, 1H), 2.95 (d, *J* = 6.3 Hz, 2H), 2.79 (ddd, *J* = 16.2, 11.4, 7.4 Hz, 1H), 2.41 (dtdd, *J* = 17.5, 6.9, 5.1, 1.5 Hz, 1H), 2.37 – 2.26 (m, 1H). ^{13}C NMR (126 MHz, Chloroform-*d*) δ 183.4, 153.6, 149.3, 142.4, 136.9, 135.4, 135.3, 133.0, 132.4, 130.3, 125.8, 121.7, 116.8, 62.5, 61.4, 41.2, 33.8, 22.6, 19.9. HRMS (ESI+): calculated for $C_{38}H_{39}O_6$ $[M+H]^+$ 591.2741, found 591.2885.

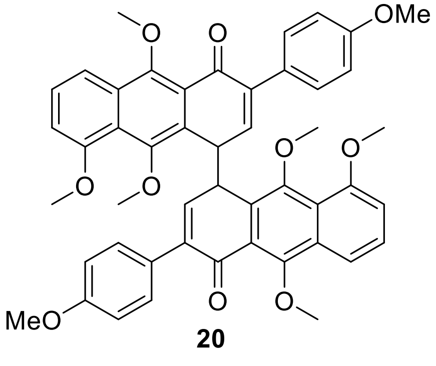

20

8,8',9,9',10,10'-hexamethoxy-3,3'-bis(4-methoxyphenyl)-[1,1'-bianthracene]-4,4'(1H,1'H)-dione (**20**) (got 37.9 mg from 40 mg **S14c**, white amorphous solid, yield =

95%) ^{1}H NMR (500 MHz, Chloroform-*d*) δ 8.04 (d, *J* = 8.5 Hz, 1H), 7.52 (t, *J* = 8.1 Hz, 1H), 7.10 – 7.04 (m, 3H), 6.75 (d, *J* = 8.7 Hz, 2H), 6.25 – 6.18 (m, 1H), 5.55 (dd, *J* = 3.3, 1.3 Hz, 1H), 4.20 (s, 3H), 4.12 (s, 3H), 4.02 (s, 3H), 3.73 (s, 3H). ^{13}C NMR (126 MHz, $CDCl_3$) δ 183.7, 159.2, 155.6, 154.4, 149.7, 143.6, 139.4, 131.9, 130.1, 129.8, 128.8, 126.8, 123.6, 123.0, 117.1, 113.5, 108.7, 63.4, 56.4, 55.3, 41.4. HRMS (ESI+): calculated for $C_{48}H_{42}NaO_{10}$ $[M+Na]^+$ 801.2670, found 801.2778.

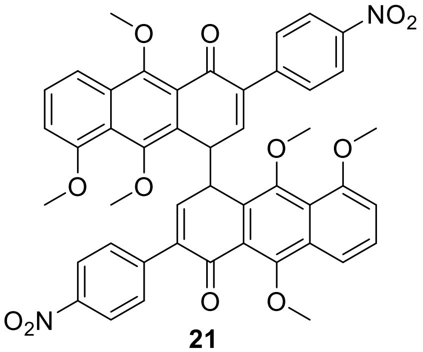

21

8,8',9,9',10,10'-hexamethoxy-3,3'-bis(4-nitrophenyl)-[1,1'-bianthracene]-4,4'(1*H*,1'*H*)-dione (**21**) (got 36.8 mg from 41 mg **S14d**, white amorphous solid, yield = 90%) ^{1}H NMR (400 MHz, Chloroform-*d*) δ 8.09 (d, *J* = 8.3 Hz, 2H), 8.02 (d, *J* = 8.5 Hz, 1H), 7.56 (t, *J* = 8.1 Hz, 1H), 7.31 (d, *J* = 8.3 Hz, 2H), 7.12 (d, *J* = 7.8 Hz, 1H), 6.42 – 6.41 (m, 1H), 5.65 – 5.59 (m, 1H), 4.20 (s, 3H), 4.13 (s, 3H), 3.99 (s, 3H). ^{13}C NMR (101 MHz, Chloroform-*d*) δ 182.9, 155.7, 154.7, 149.9, 147.3, 142.5, 142.4, 142.1, 132.0, 129.5, 128.5, 127.5, 123.3, 123.2, 122.9, 117.1, 109.1, 63.5, 63.4, 56.4, 41.5. HRMS (ESI+): calculated for $C_{46}H_{36}N_2NaO_{12}$ $[M+Na]^+$ 831.2160, found 831.2263.

6. X-Ray Crystal Structure and Data

Compound 15

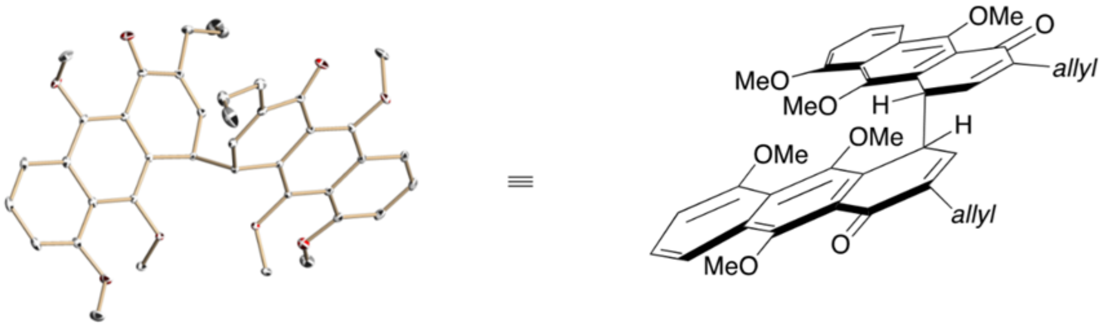

Figure S1. Crystal structure of **15**

Table S1. Crystal data and structure refinement parameters of **15**.

Compound	**15**
Empirical formula	$C_{40}H_{38}O_8$
Formula weight	646.70
Temperature (K)	100(2)
Wavelength (Å)	0.71073
Crystal system	Orthorhombic
Space group	*Pbcn*
a (Å)	17.9510(8)
b (Å)	9.3659(4)
c (Å)	19.3161(8)
α (°)	90.00
β (°)	90.00
γ (°)	90.00
V (Å^3)	3247.6(2)
Z	4
ρ_{calcd} (g·cm^{-3})	1.323
μ (mm^{-1})	0.092
F(000)	1368
Crystal size (mm)	0.07×0.11×0.23
θ range for data collection (°)	3.01-29.58
Reflections collected	34080
Independent reflections	4526
	[R_{int} = 0.0583]
Transmission factors (min/max)	0.6784/0.7459
Data/restraints/params.	4526/0/220

$R1,^a$ $wR2^b$ $(I > 2\sigma(I))$	0.0570, 0.1145
$R1,^a$ $wR2^b$ (all data)	0.0892, 0.1274
Quality-of-fit[c]	1.059

$R_{int} = \Sigma|F_o^2 - <F_o^2>|/\Sigma|F_o^2|$
$^aR1 = \Sigma||F_o|-|F_c||/\Sigma|F_o|$. $^bwR2 = [\Sigma[w(F_o^2-F_c^2)^2]/\Sigma[w(F_o^2)^2]]$.
^{c}Quality-of-fit = $[\Sigma[w(F_o^2-F_c^2)^2]/(N_{obs}-N_{params})]^{1/2}$, based on all data.

Compound 19

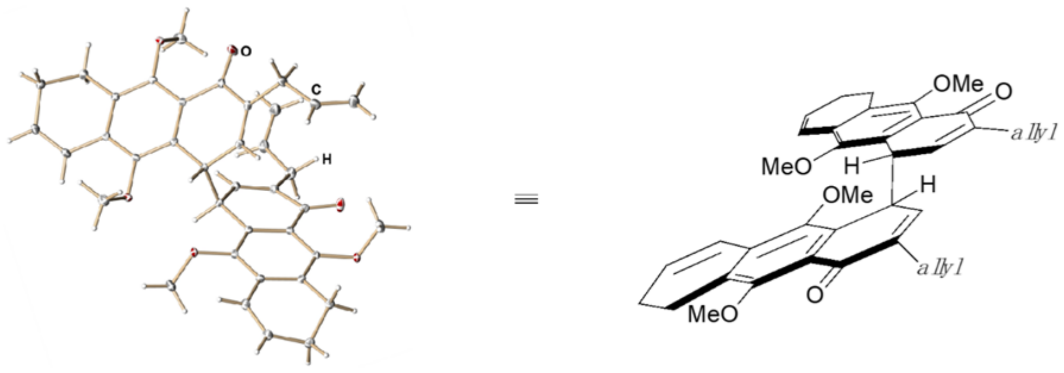

Figure S2. Crystal structure of **19**

Table S2. Crystal data and structure refinement parameters of **19**.

Compound	**19**
Empirical formula	$C_{38}H_{38}O_6$
Formula weight	590.68
Temperature (K)	100(2)
Wavelength (Å)	0.71073
Crystal system	orthorhombic
Space group	*Fdd*2
a (Å)	14.4527(6)
b (Å)	44.129(2)
c (Å)	9.4918(6)
α (°)	90

β (°)	90
γ (°)	90
V (Å^3)	6053.7(5)
Z	8
$\rho_{\chi\alpha\lambda\chi\delta}$ (γ•χμ^{-3})	1.296
μ (μμ^{-1})	0.087
F(000)	2512
Crystal size (mm)	0.08×0.16×0.45
θ range for data collection (°)	3.37-29.16
Reflections collected	50148
Independent reflections	4067 [R_{int} = 0.0312]
Transmission factors (min/max)	0.7122/0.7458
Data/restraints/params.	4067/1/201
$R1$,[a] $wR2$[b] ($I > 2s(I)$)	0.0344, 0.0835
$R1$,[a] $wR2$[b] (all data)	0.0375, 0.0854
Quality-of-fit[c]	1.058

$R_{int} = \Sigma|F_o^2 - <F_o^2>|/\Sigma|F_o^2|$

[a]$R1 = \Sigma||F_o|-|F_c||/\Sigma|F_o|$. [b]$wR2 = [\Sigma[w(F_o^2-F_c^2)^2]/\Sigma[w(F_o^2)^2]]$.

[c]Quality-of-fit = $[\Sigma[w(F_o^2-F_c^2)^2]/(N_{obs}-N_{params})]^{½}$, based on all data.

7. Mechanism

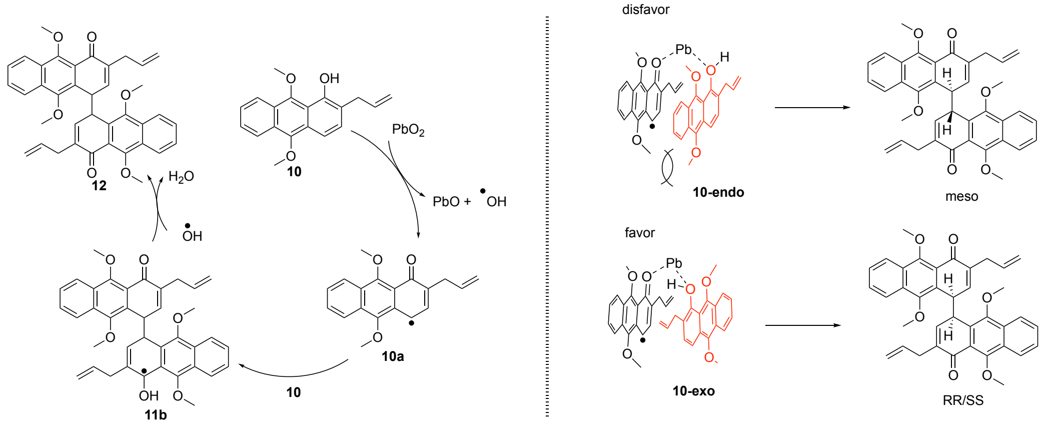

Dimerization

A proposed mechanism is presented in figure above. We believe that dimerization is initiated by the PbO_2-mediated oxidation of compound **10**, generating the anthracenyl radical **10a**. This radical can combine with another molecule of **10** to form intermediate **11b**, which is subsequently oxidized by a hydroxyl radical to yield product **12**.

Selectivity for generating enone dimer.

We propose that the re-aromatization was prevented due to kinetic trapping. The bulky substituents on the B and C rings of **12** create steric hindrance, stabilizing the enone adduct and imposing a significant energy barrier to forming the thermodynamic biaryl product by restricting the necessary conformational changes or reactions for re-aromatization. In our experiments, heating enone dimer **12** at 110°C in toluene yielded the corresponding biaryl adduct. Conversely, the absence of substituents on either the B or C ring resulted exclusively in the biaryl product. These observations confirm that steric bulkiness causes enone dimers to remain as kinetically trapped stable products over biaryl isomers.

Selectivity of RR/SS configuration.

We proposed a tentative working model to explain the observed selectivity (refer to the figure above). Upon the formation of the anthracenyl radical, Pb might chelate between the enone radical and anthracenol **10**. This interaction could result in two possible configurations, labelled as **10-endo** and **10-exo**. While the **10-endo** pathway might benefit from π-π stacking interactions, the B-ring methoxy groups could introduce significant steric hindrance, making the **10-endo** configuration unfavorable. Conversely, the **10-exo** arrangement minimizes steric interference, thereby favoring the formation of the RR/SS diastereomers as the preferred products.

8. Reference

1) SAINT; part of Bruker APEX3 software package (version 2018.7-2): Bruker AXS, 2018.
2) SADABS; part of Bruker APEX3 software package (version 2018.7-2): Bruker AXS, 2018.
3) G. M. Sheldrick, Acta Crystallogr. 2015, A71, 3-8.
4) G. M. Sheldrick, Acta Crystallogr. 2015, C71, 3-8.
5) O. V. Dolomanov, L. J. Bourhis, R. J. Gildea, J. A. K. Howard, H. Puschmann, J. Appl. Crystallogr. 2009, 42, 339-341.
6) Chen, Q.; Zhong, Y.; O'Doherty, G. A. J. C. C., Convergent De Novo Synthesis of Vineomycinone B 2 Methyl Ester. Chem. Commun. 2013, 49, 6806-6808.
7) Baldwin, J. E.; Rajeckas, A. J., Approaches to the Regiospecific Synthesis of Anthracycline Antibiotics. Tetrahedron 1982, 38, 3079-3084.

9. Spectrum data

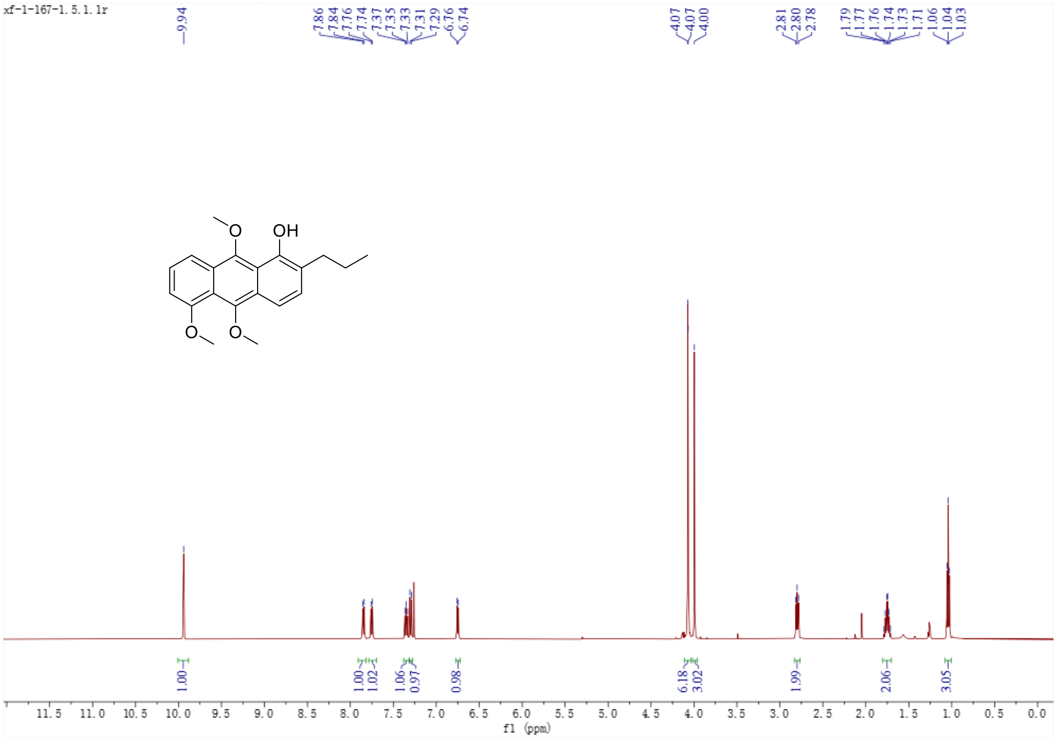

500 MHz, ^{1}H-NMR of substrate **S2** in $CDCl_3$

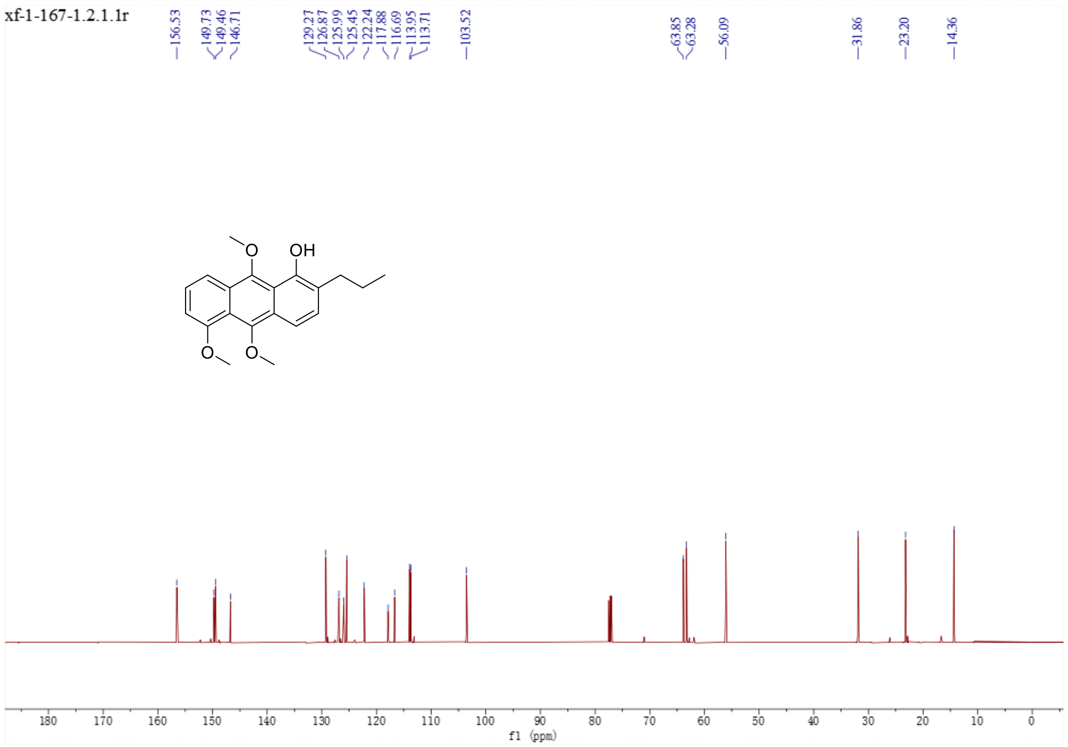

126 MHz, ^{13}C-NMR of substrate **S2** in $CDCl_3$

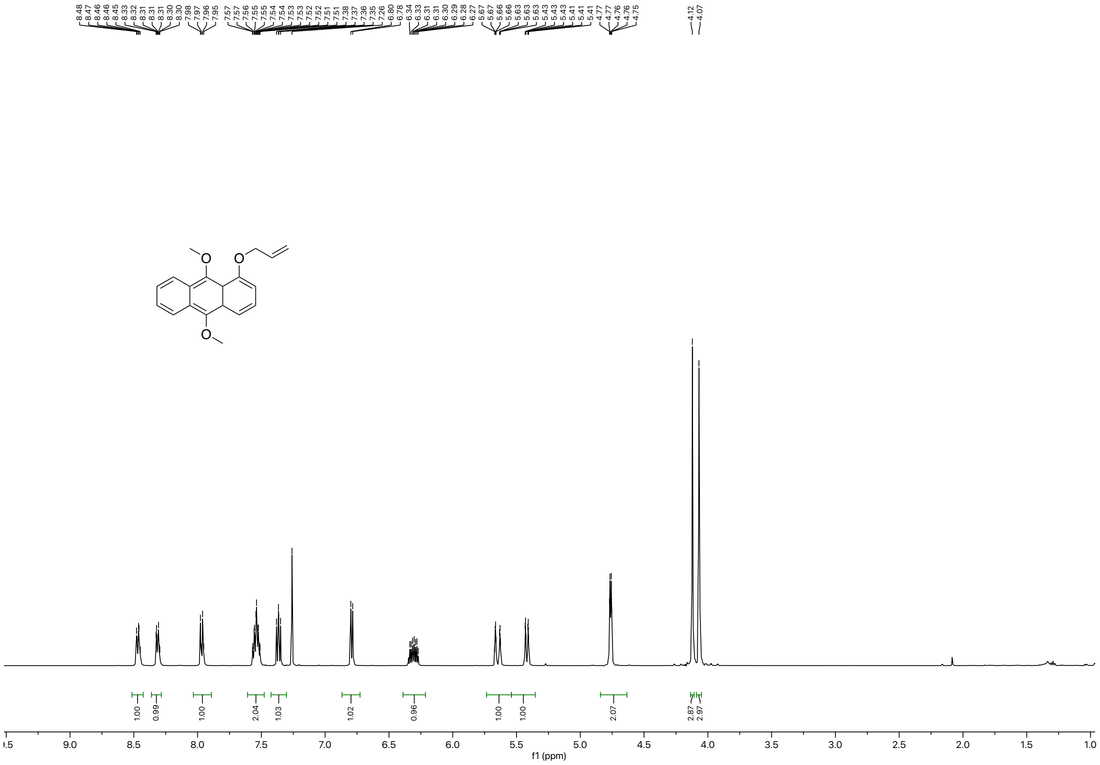

500 MHz, ^{1}H-NMR of substrate **9** in $CDCl_3$

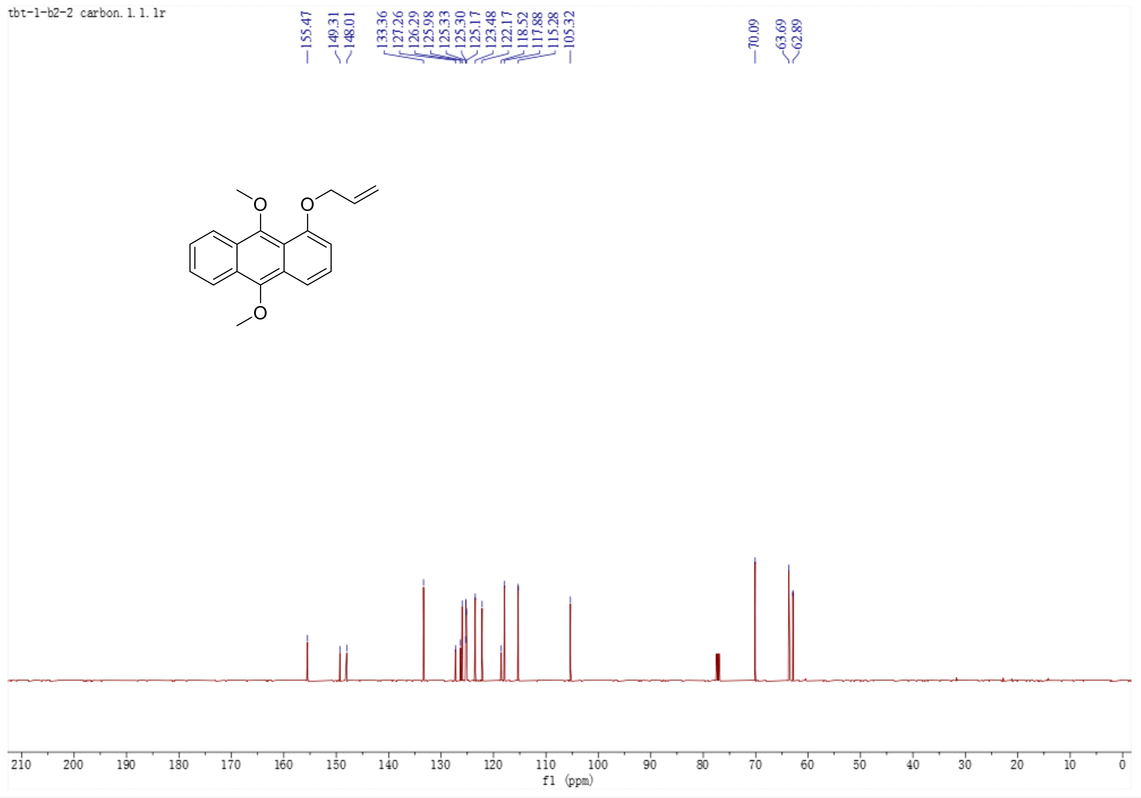

126 MHz, ^{13}C-NMR of substrate **9** in $CDCl_3$

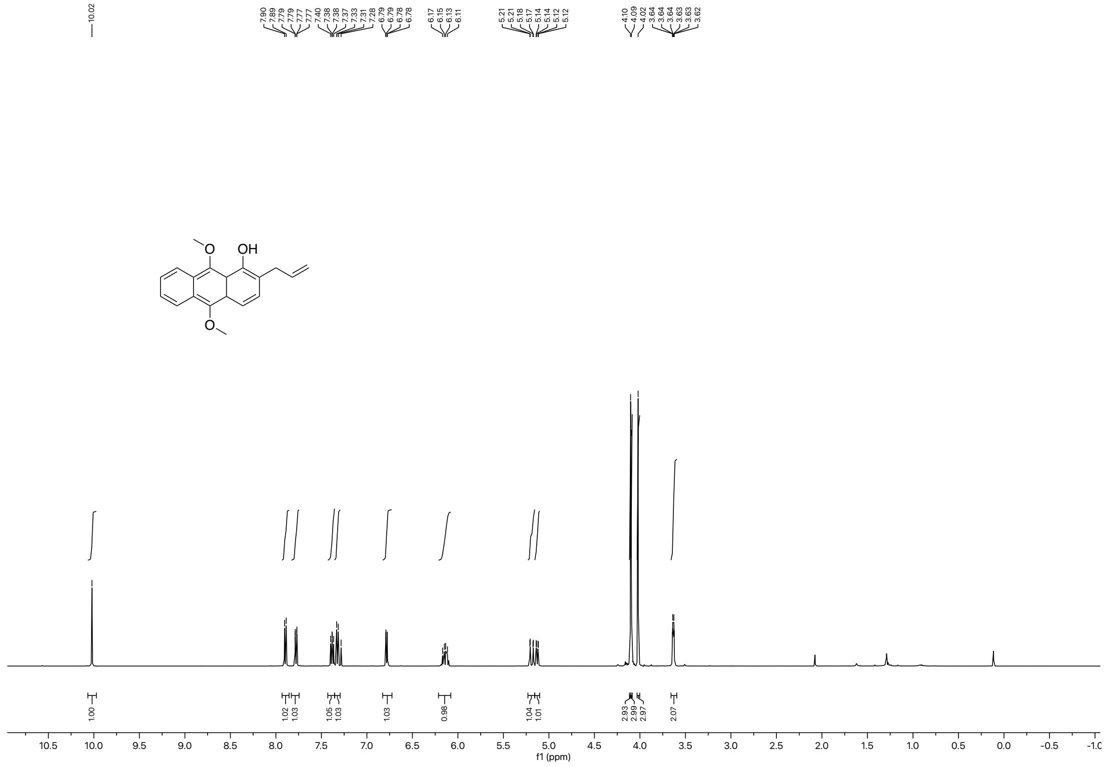

500 MHz, ^{1}H-NMR of substrate **10** in $CDCl_3$

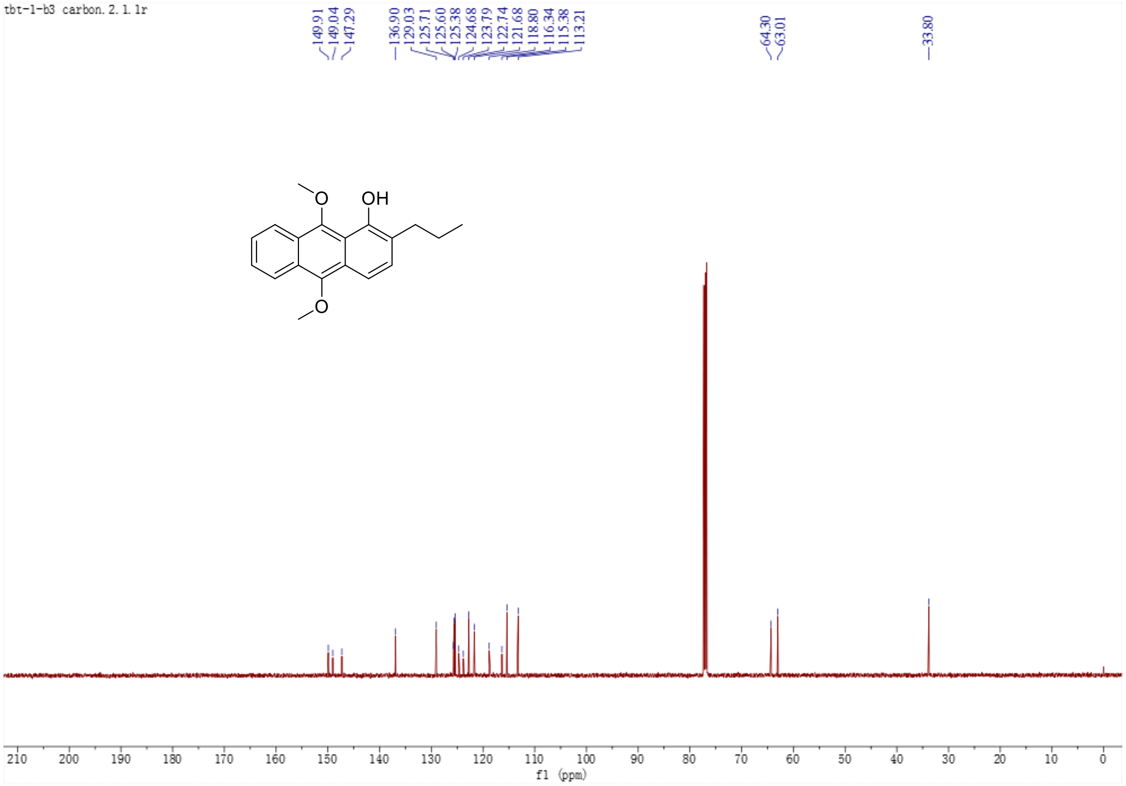

126 MHz, ^{13}C-NMR of substrate **10** in $CDCl_3$

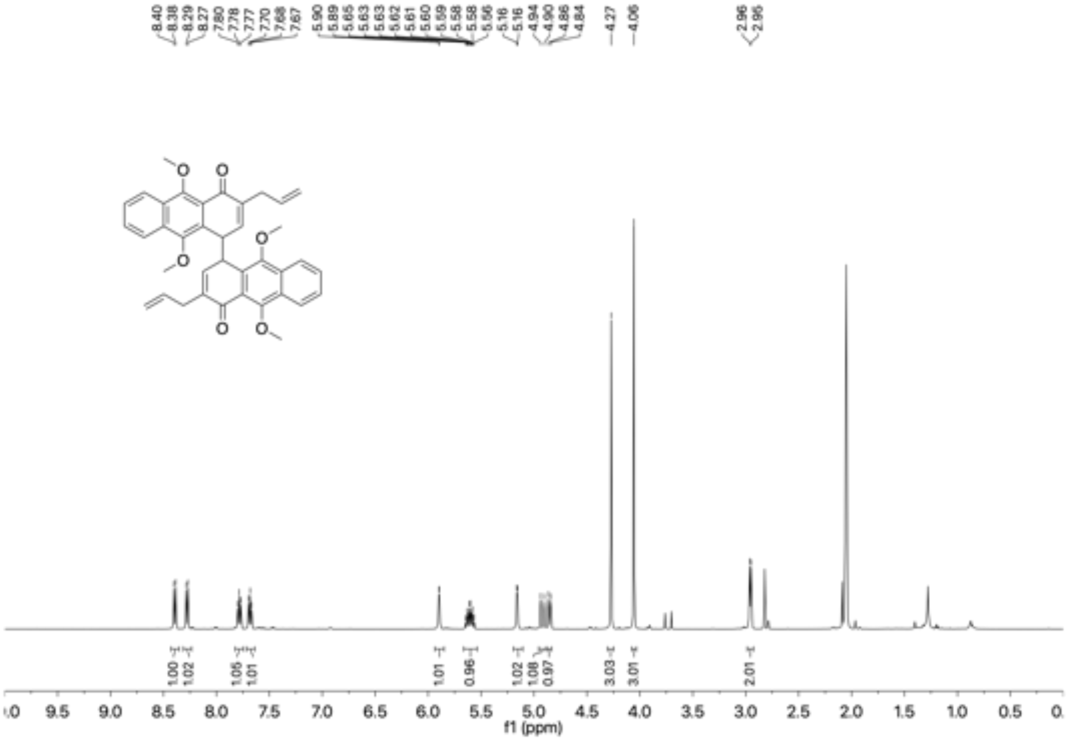

500 MHz, ^{1}H-NMR of substrate **12** in Acetone-d_6

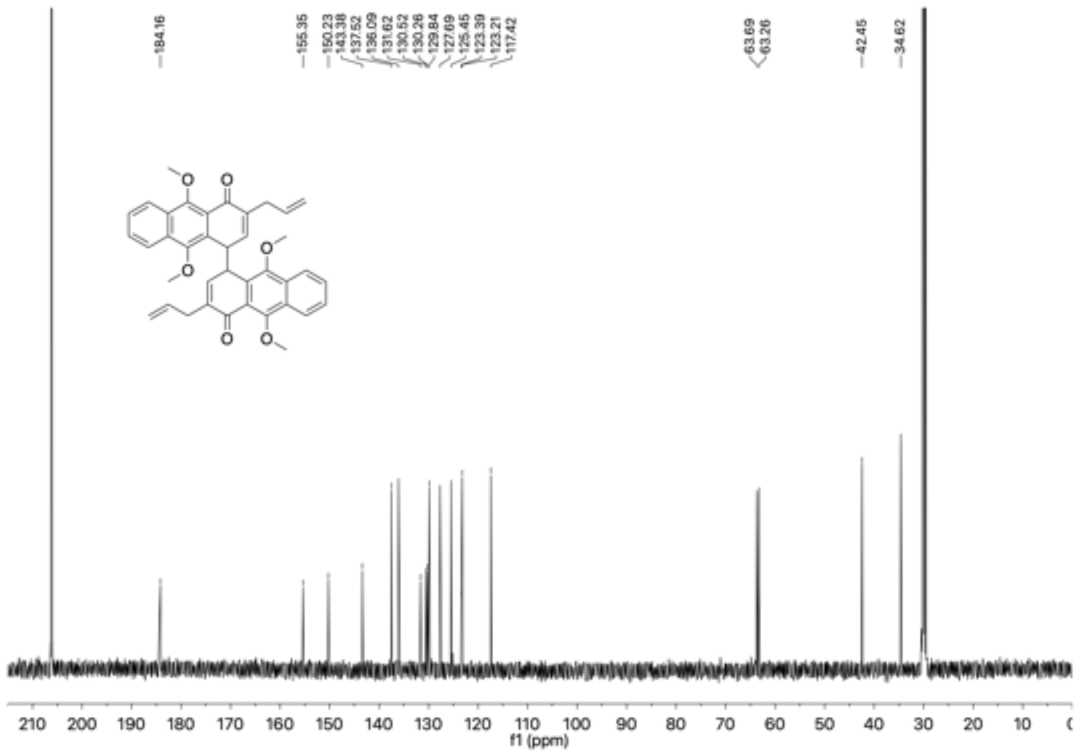

126 MHz, ^{13}C-NMR of substrate **12** in Acetone-d_6

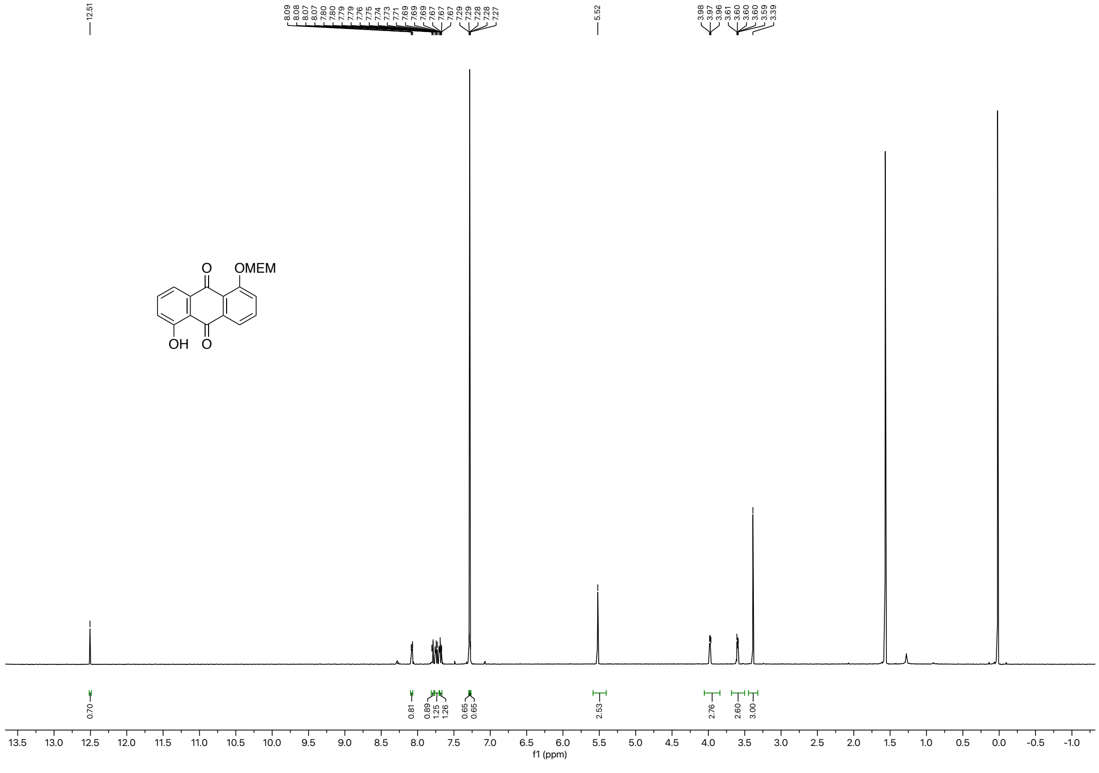

500 MHz, ^{1}H-NMR of substrate **S4** in $CDCl_3$

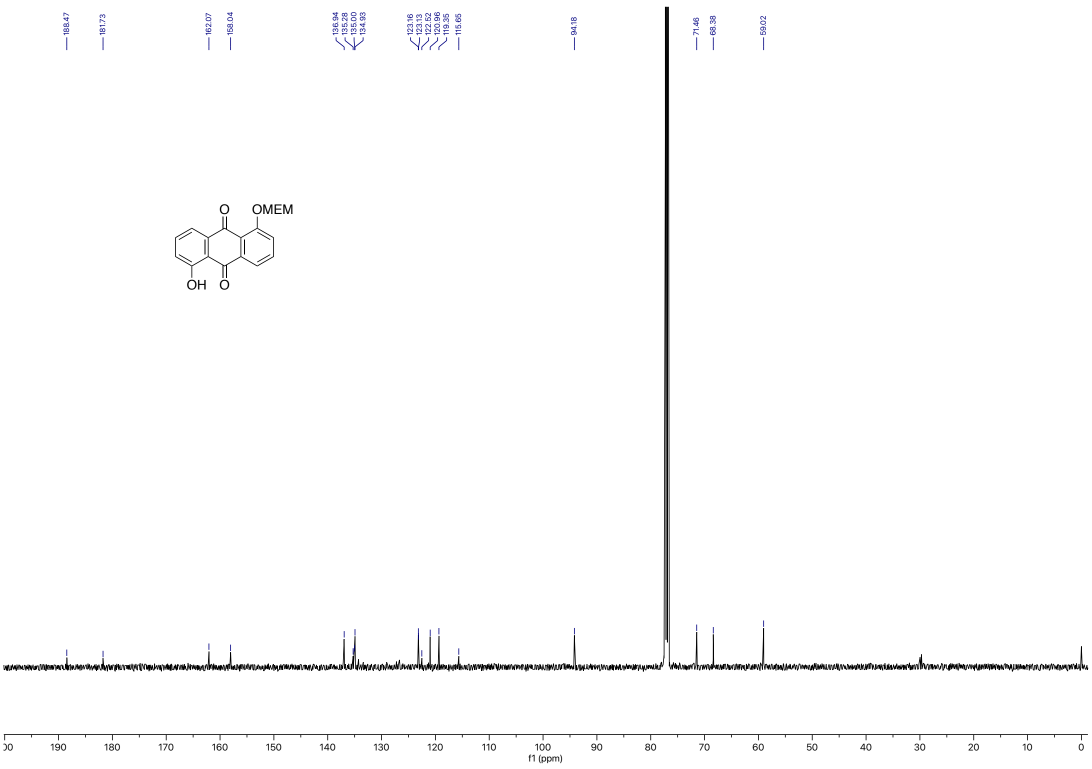

126 MHz, ^{13}C-NMR of substrate **S4** in $CDCl_3$

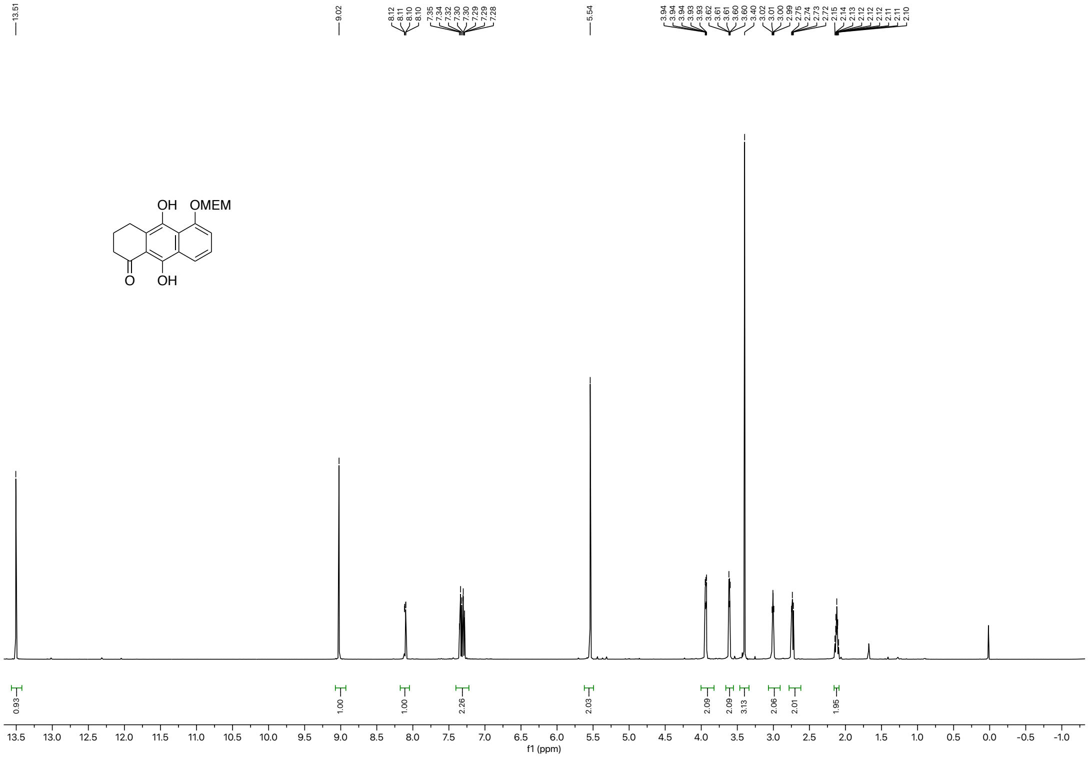

500 MHz, ^{1}H-NMR of substrate **S5** in $CDCl_3$

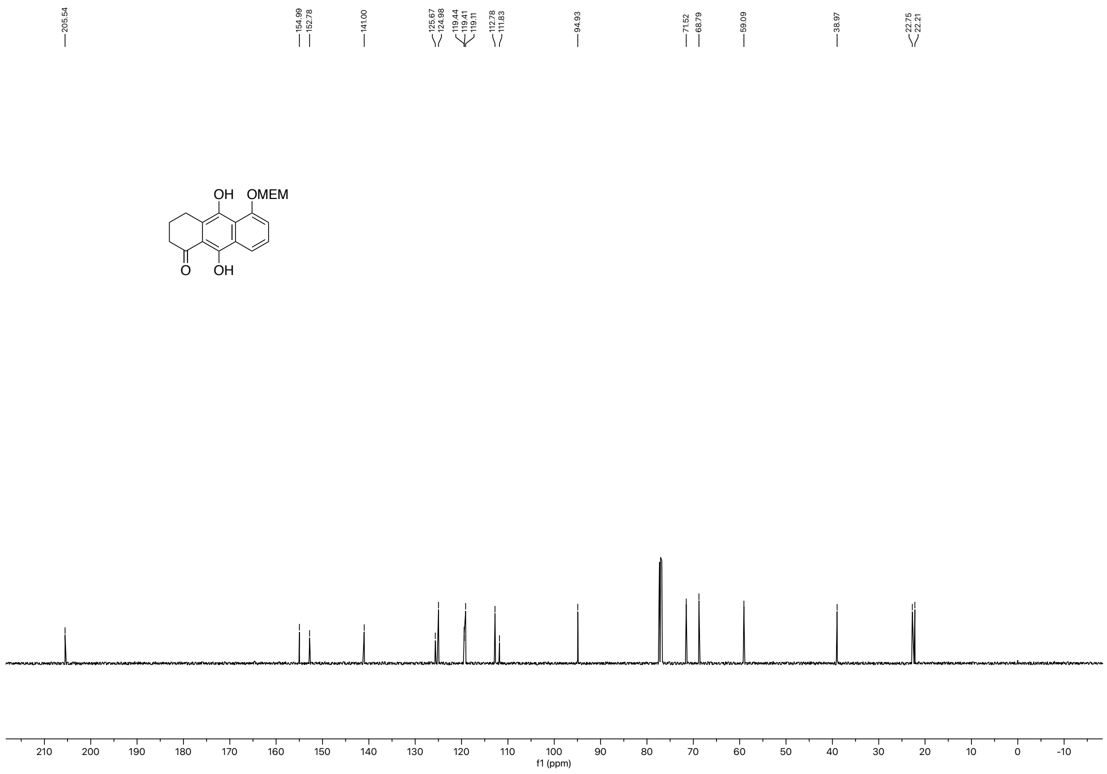

126 MHz, ^{13}C-NMR of substrate **S5** in $CDCl_3$

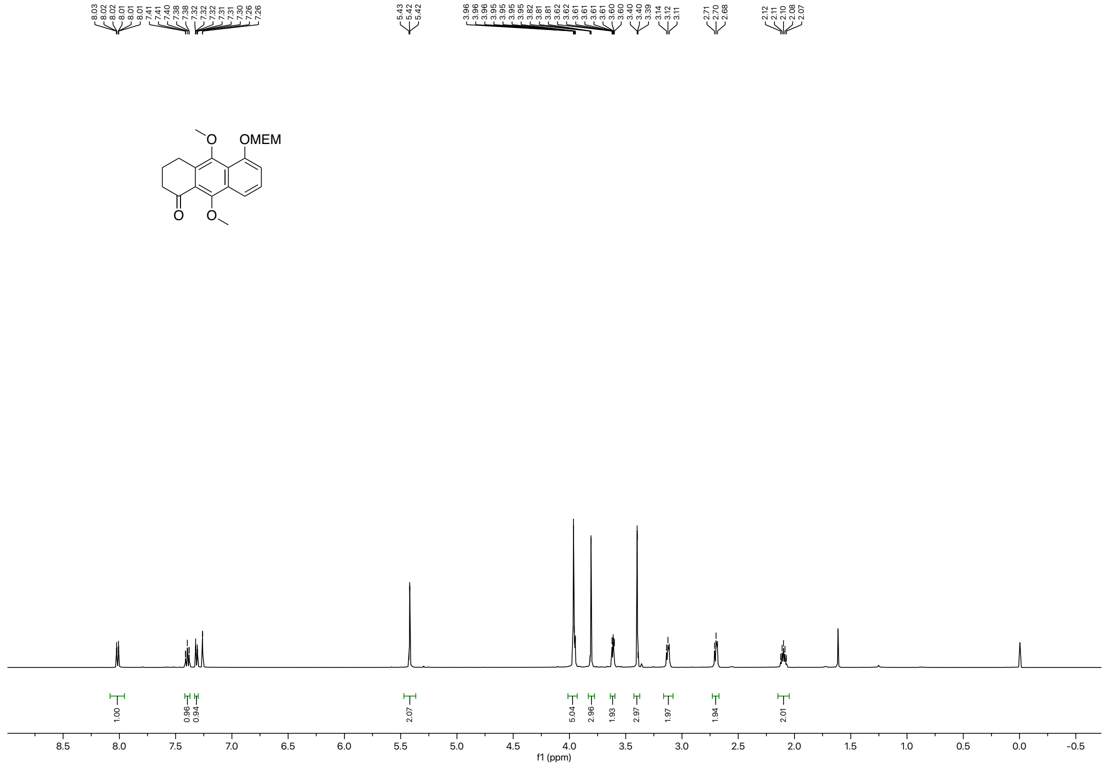

500 MHz, ^{1}H-NMR of substrate **S6** in $CDCl_3$

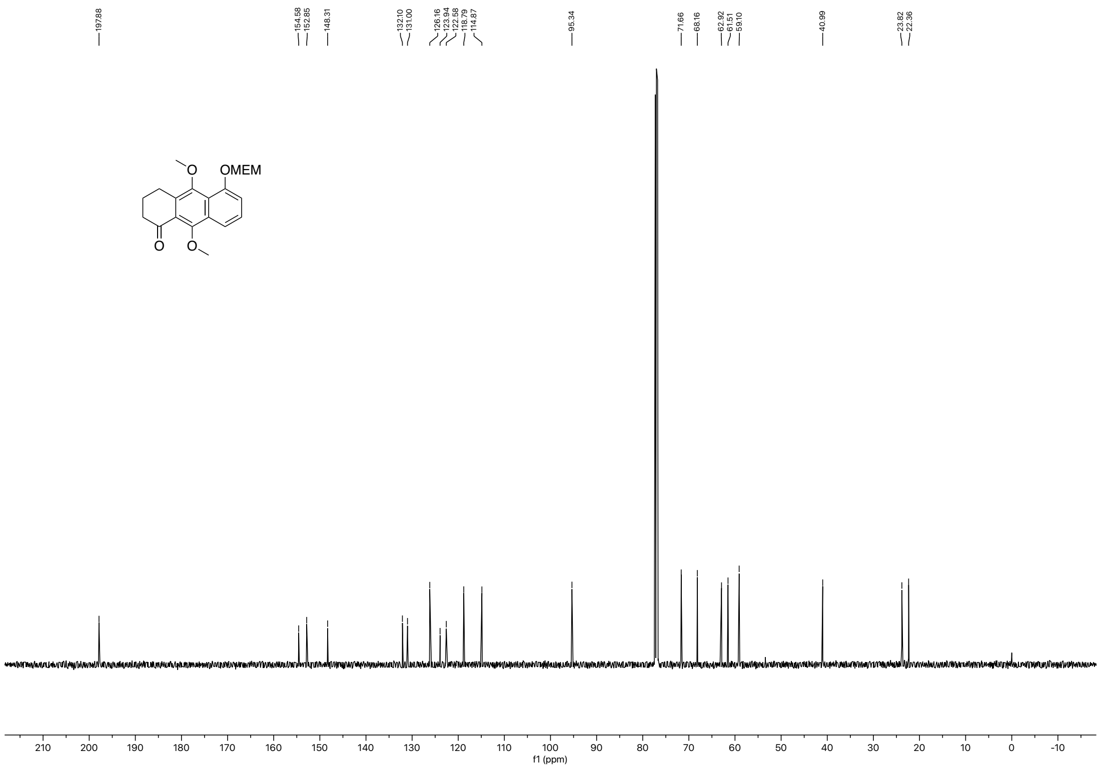

126 MHz, ^{13}C-NMR of substrate **S6** in $CDCl_3$

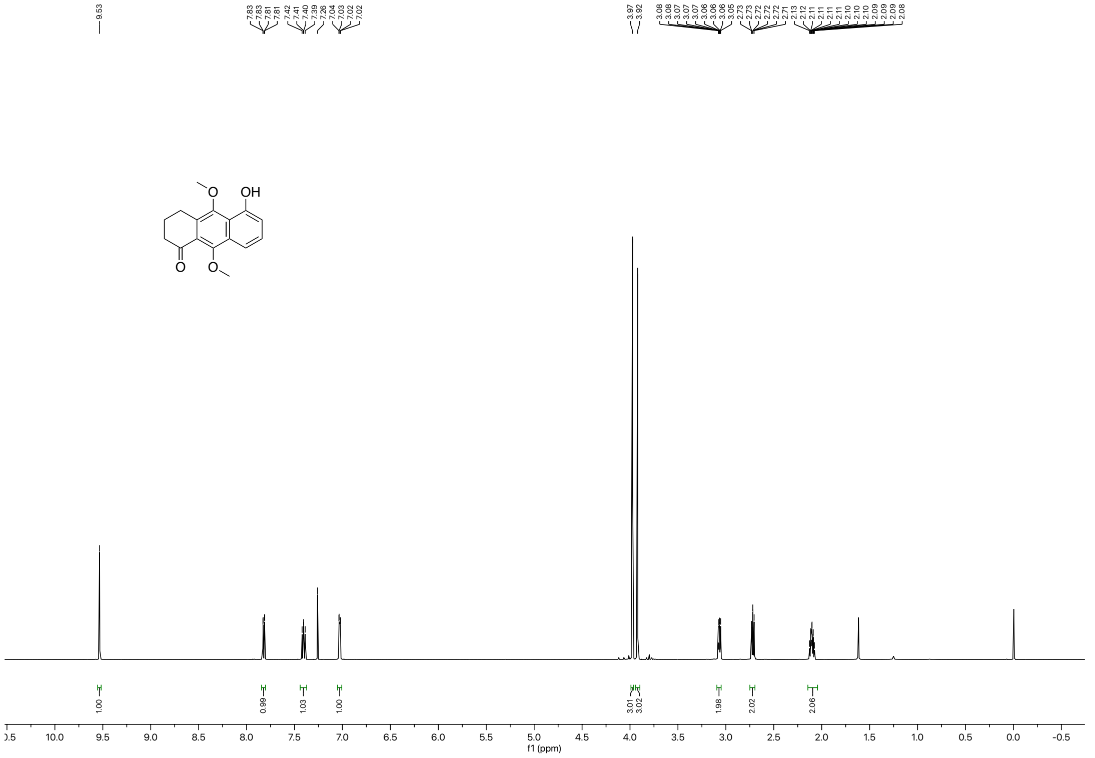

500 MHz, ^{1}H-NMR of substrate **S7** in $CDCl_3$

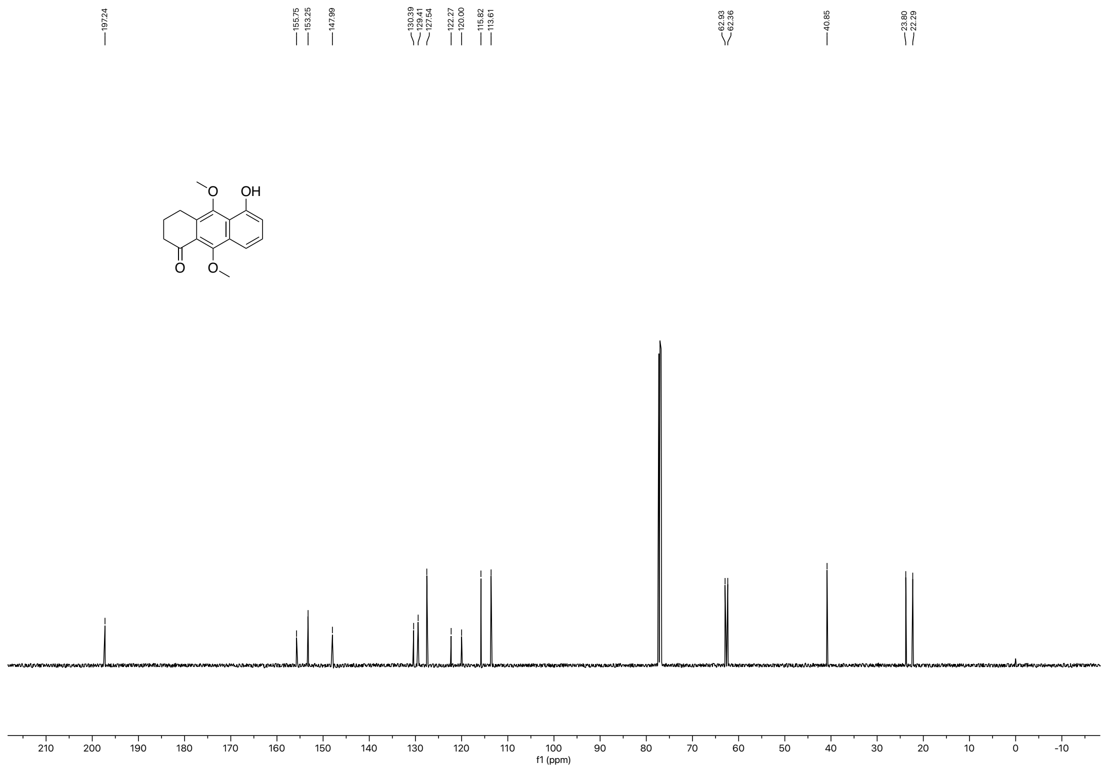

126 MHz, ^{13}C-NMR of substrate **S7** in $CDCl_3$

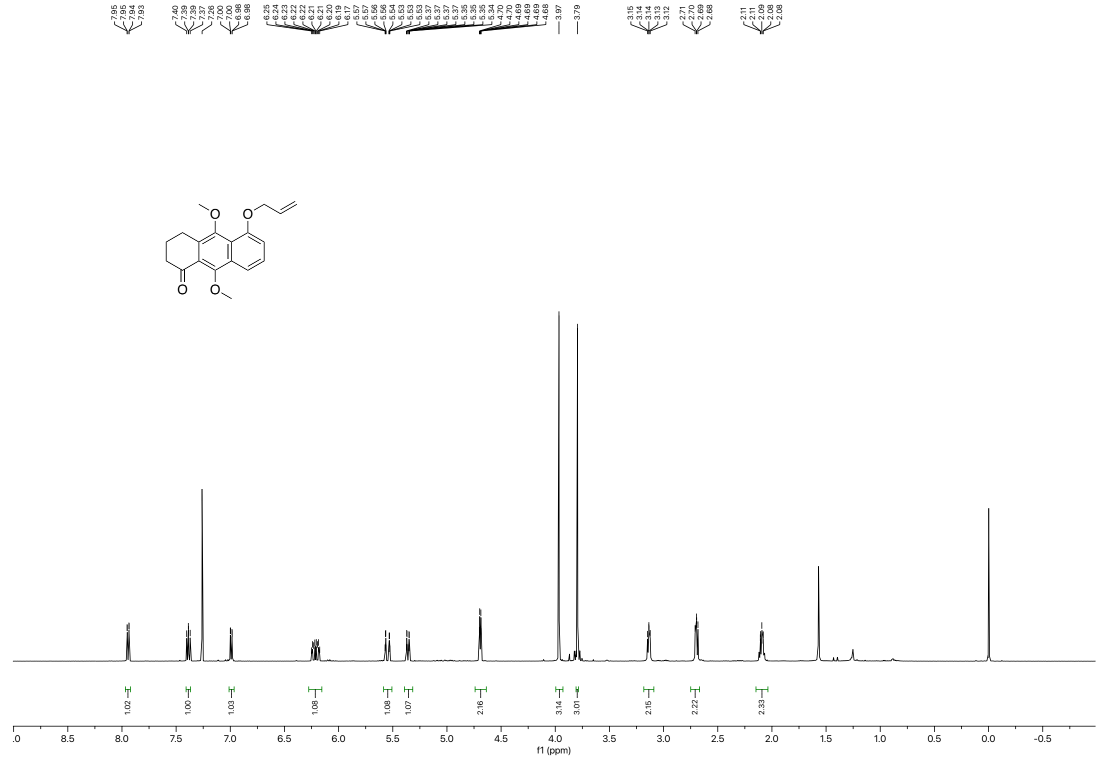

500 MHz, ^{1}H-NMR of substrate **S8** in $CDCl_3$

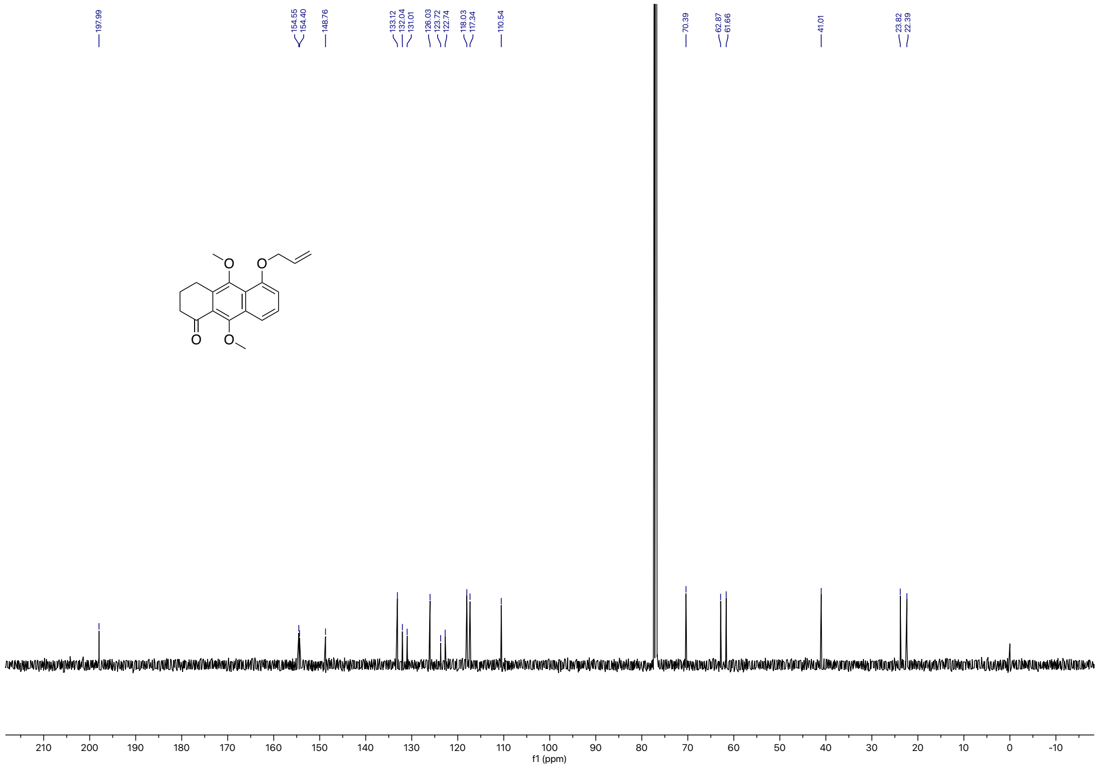

126 MHz, ^{13}C-NMR of substrate **S8** in $CDCl_3$

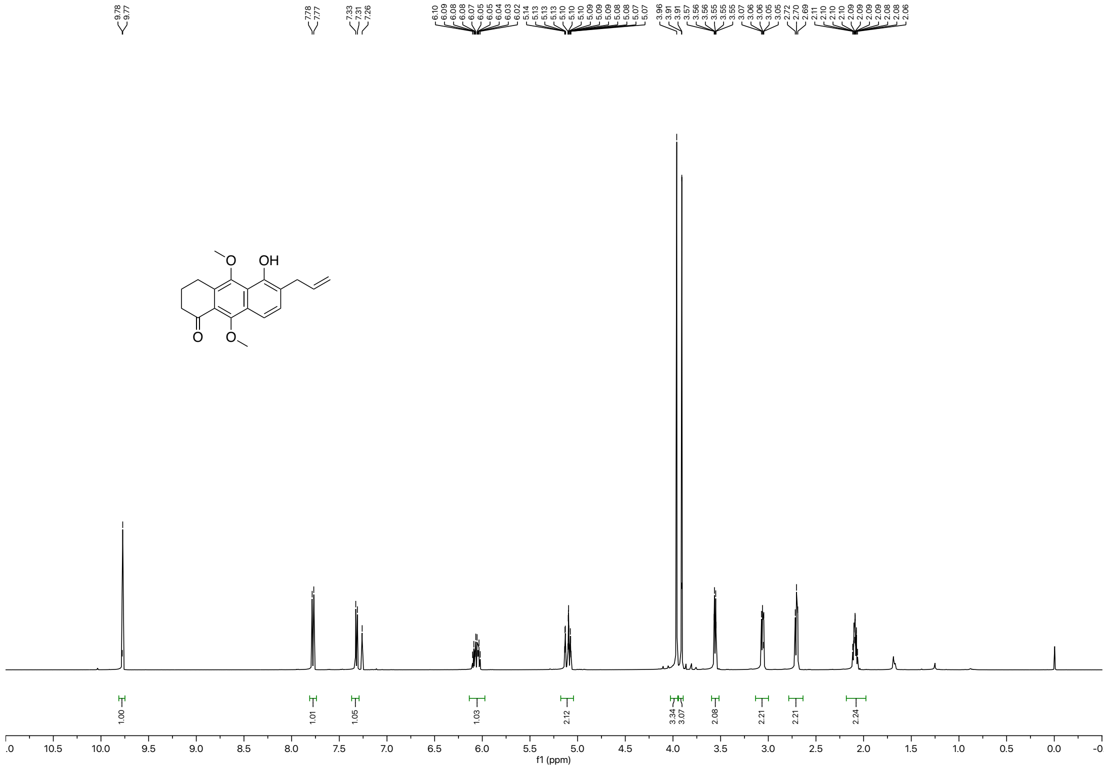

500 MHz, ^{1}H-NMR of substrate **S9** in $CDCl_3$

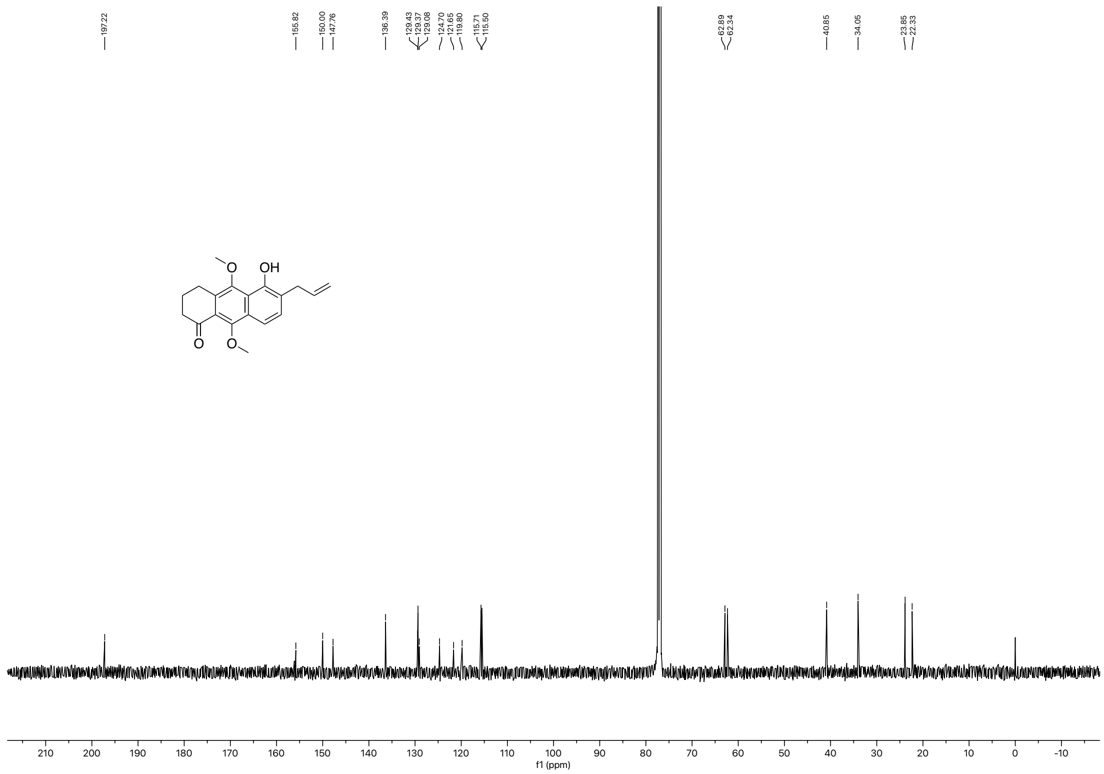

126 MHz, ^{13}C-NMR of substrate **S9** in $CDCl_3$

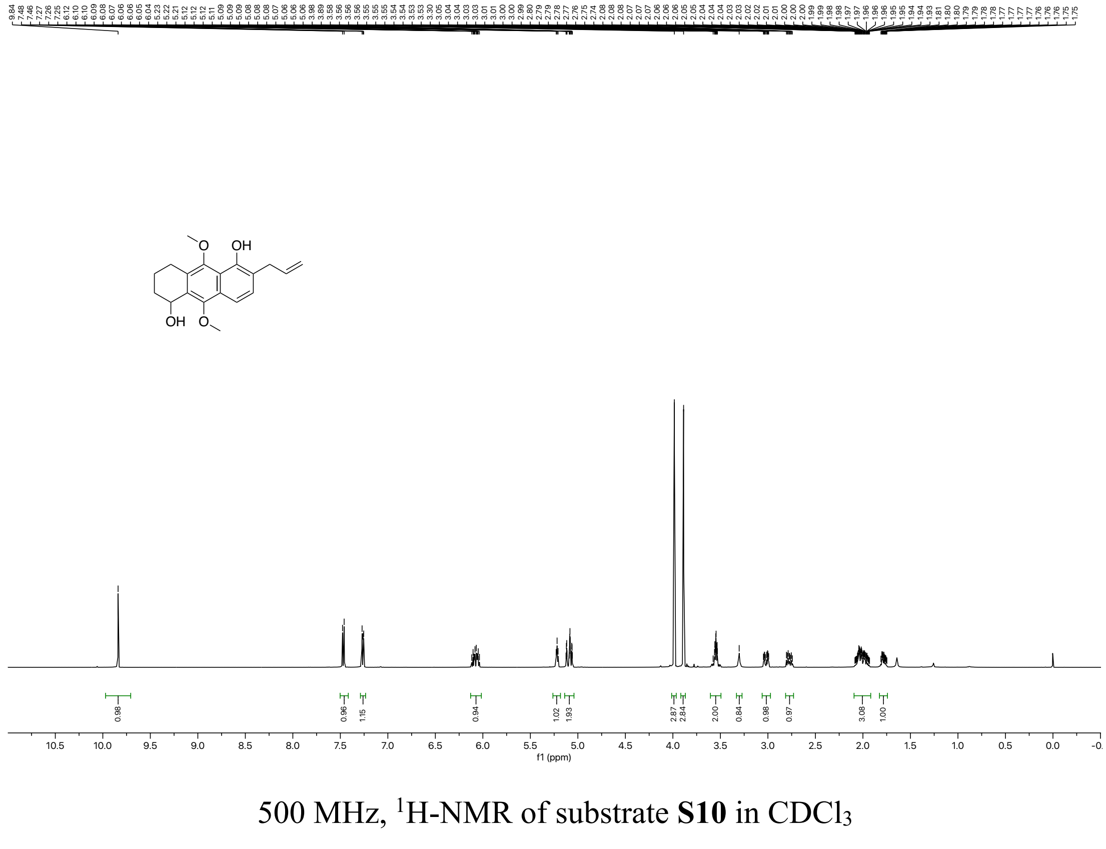

500 MHz, ^{1}H-NMR of substrate **S10** in $CDCl_3$

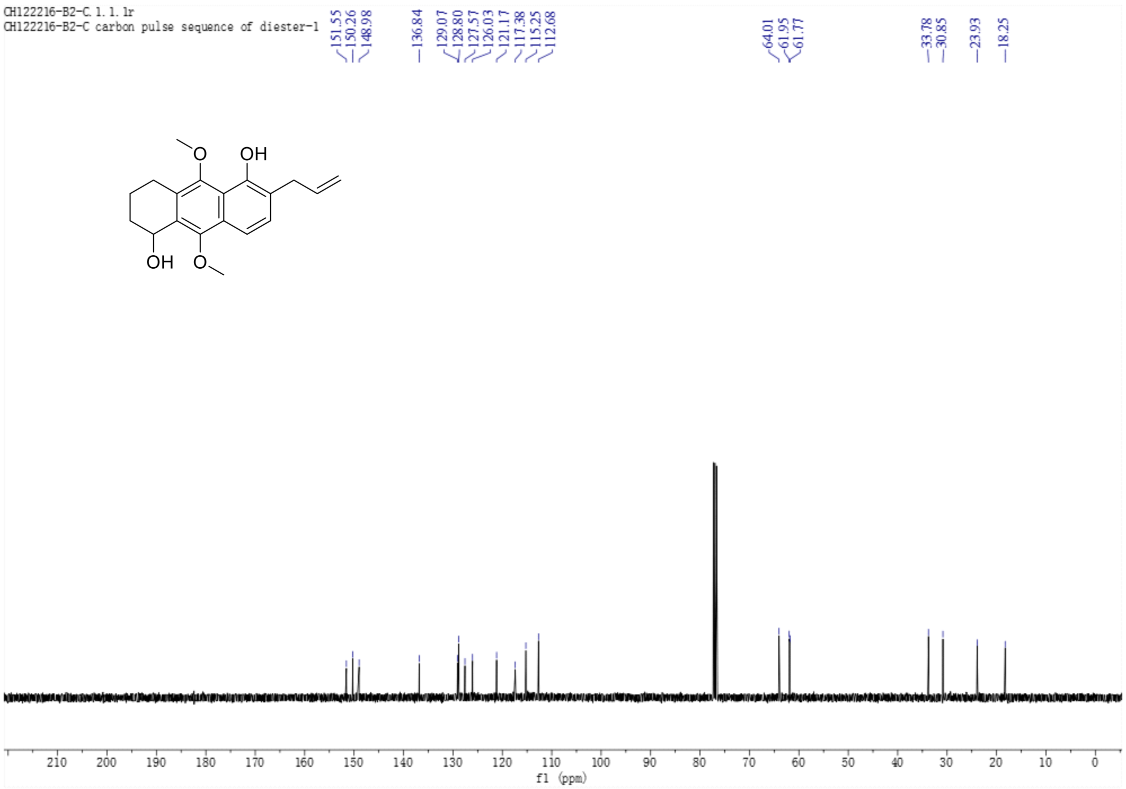

126 MHz, ^{13}C-NMR of substrate **S10** in $CDCl_3$

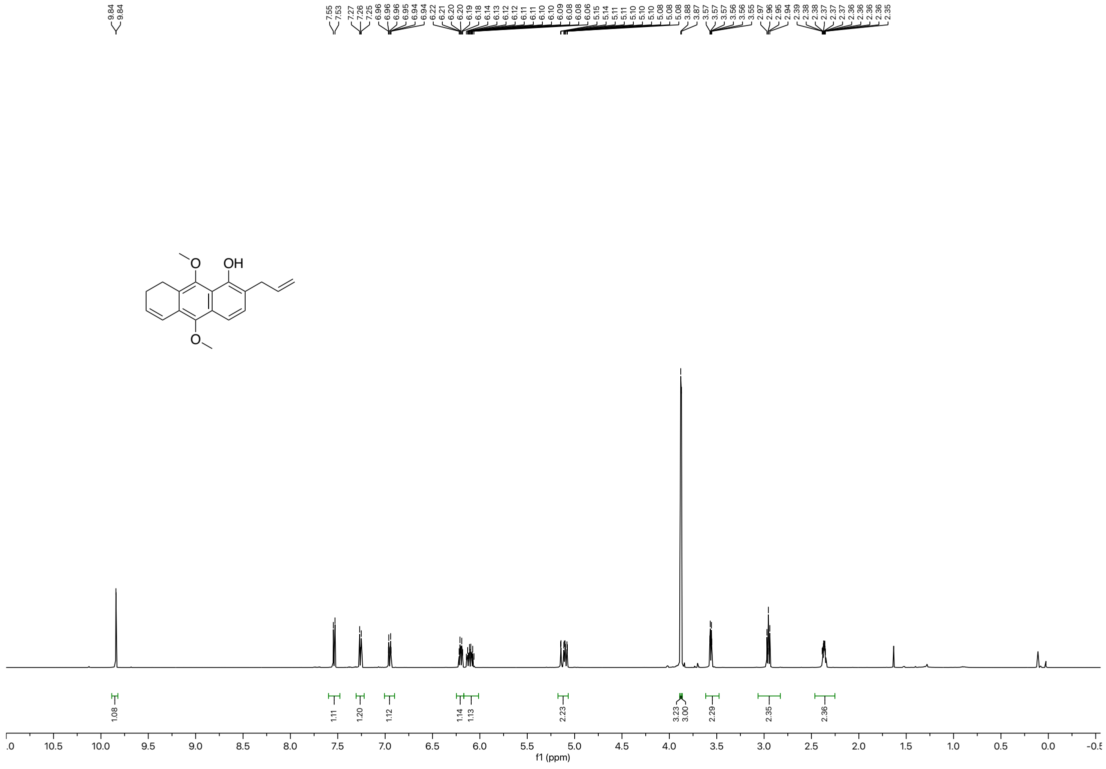

500 MHz, ^{1}H-NMR of substrate **S11** in $CDCl_3$

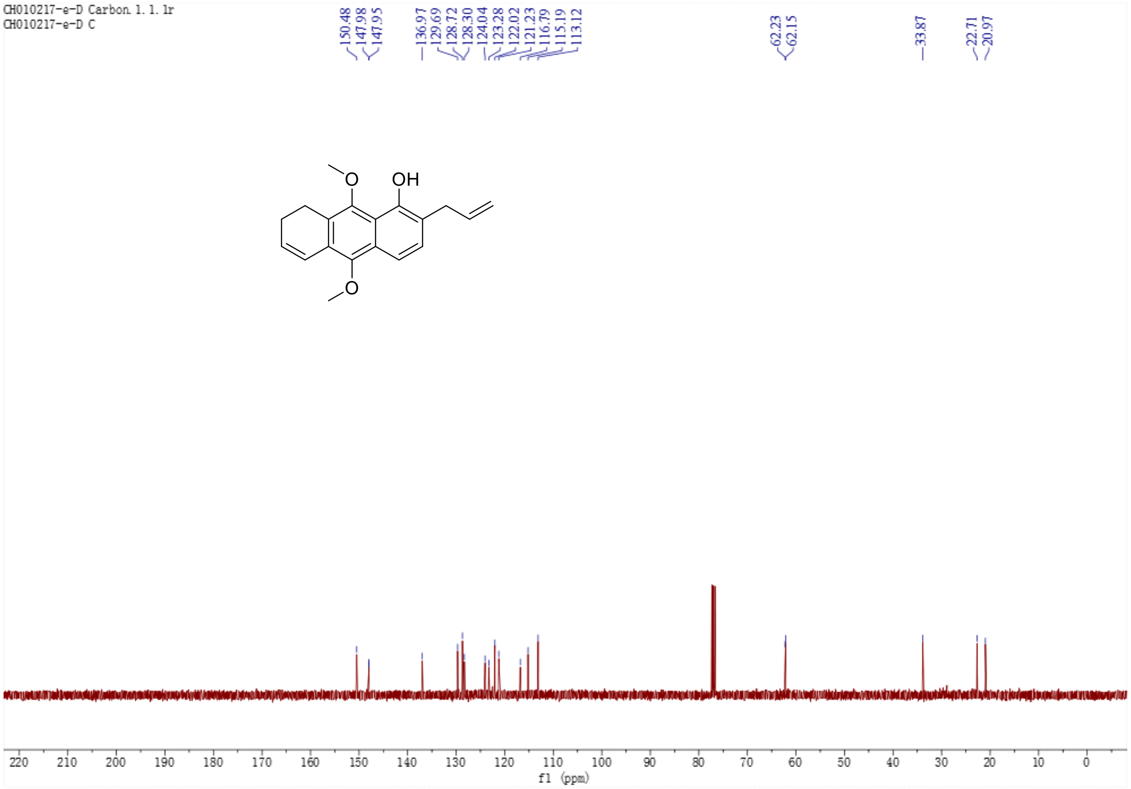

126 MHz, ^{13}C-NMR of substrate **S11** in $CDCl_3$

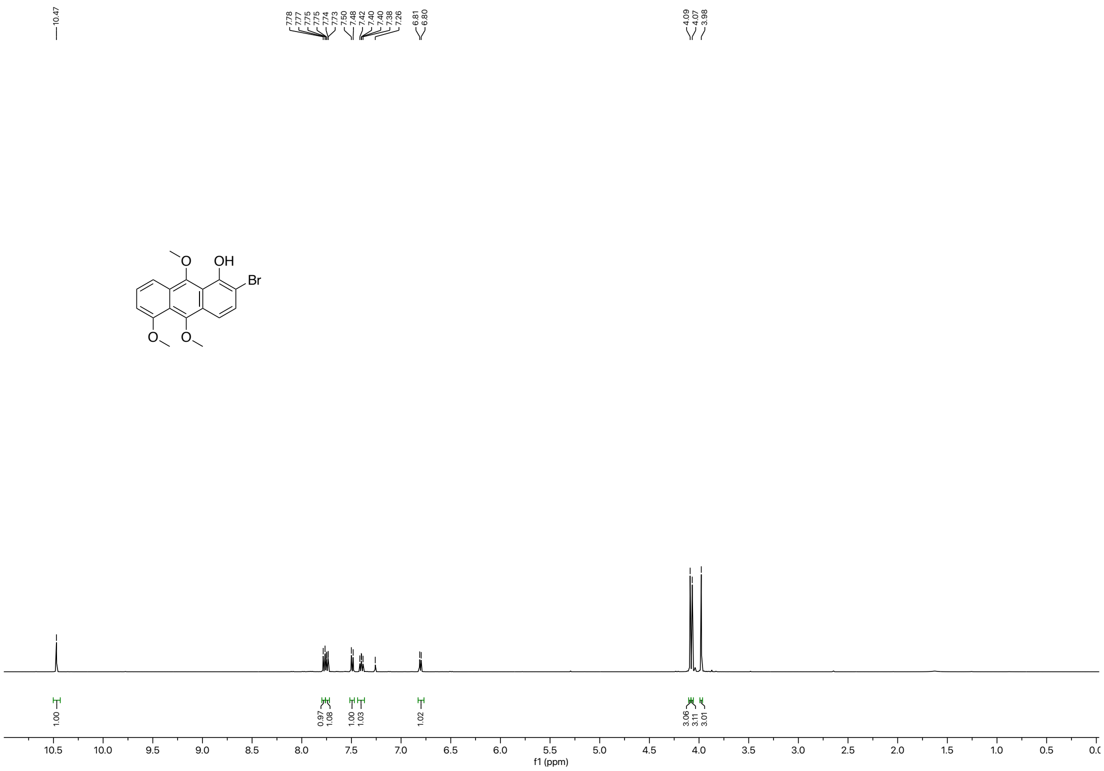

500 MHz, ^{1}H-NMR of substrate **S13** in $CDCl_3$

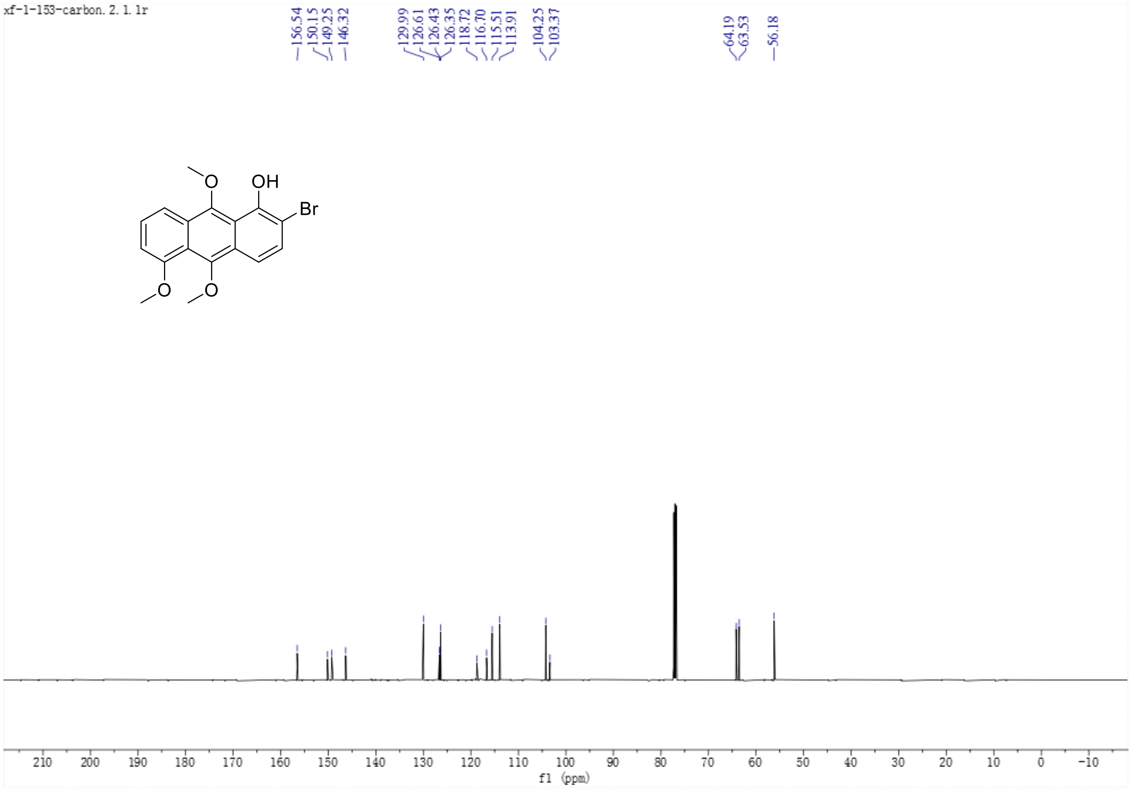

126 MHz, ^{13}C-NMR of substrate **S13** in $CDCl_3$

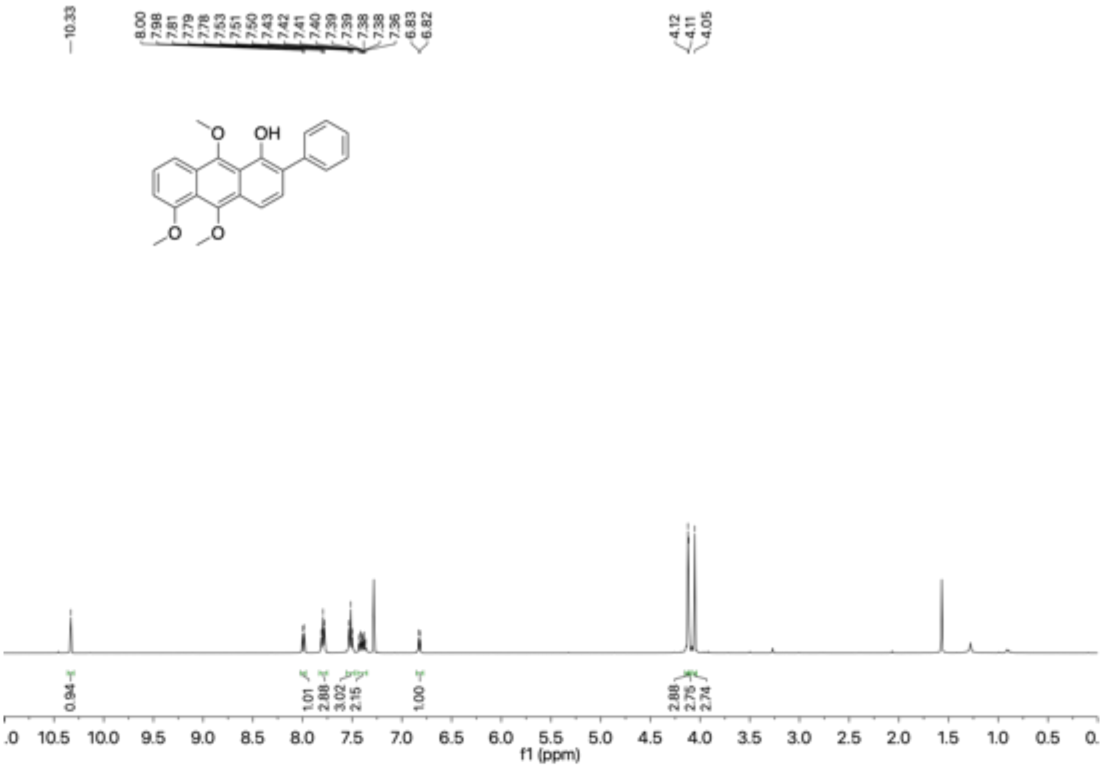

500 MHz, ^{1}H-NMR of substrate **S14a** in $CDCl_3$

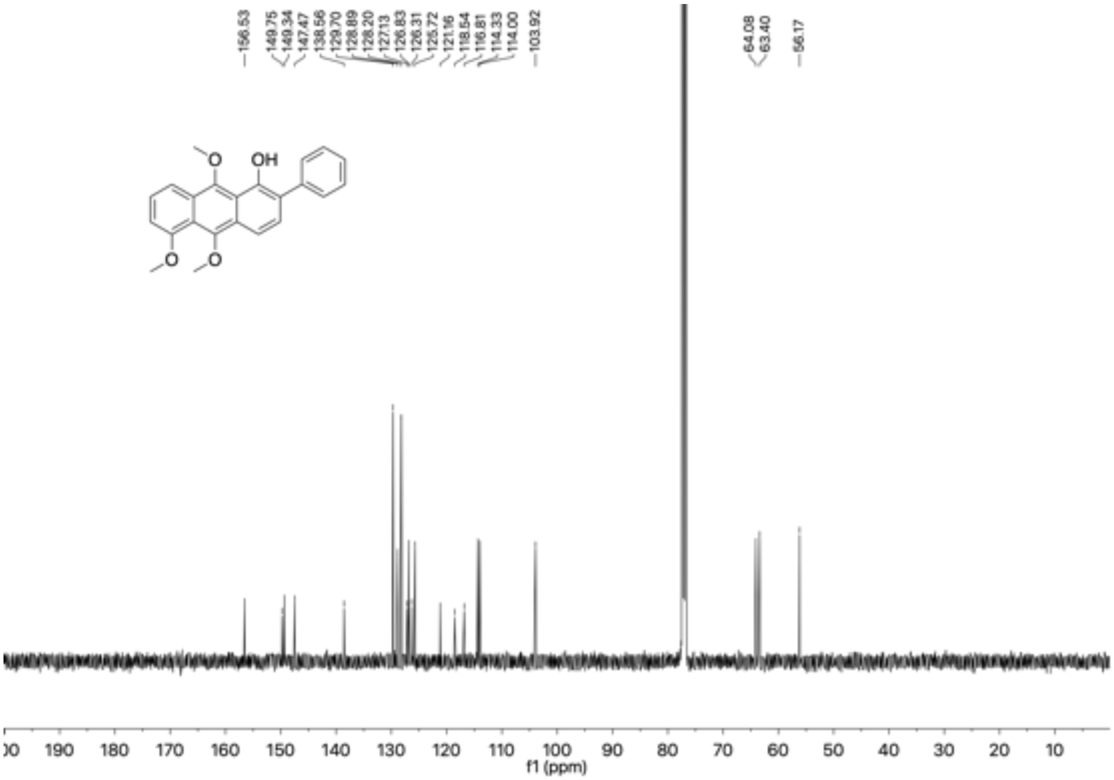

126 MHz, ^{13}C-NMR of substrate **S14a** in $CDCl_3$

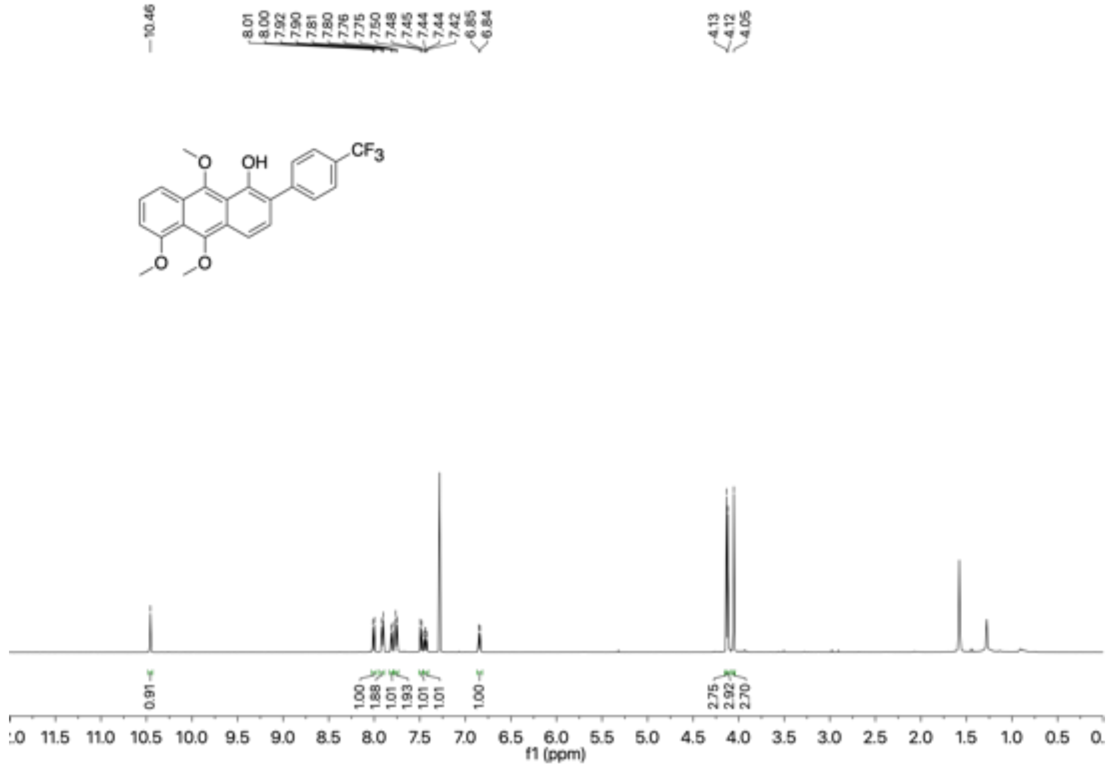

500 MHz, ^{1}H-NMR of substrate **S14b** in $CDCl_3$

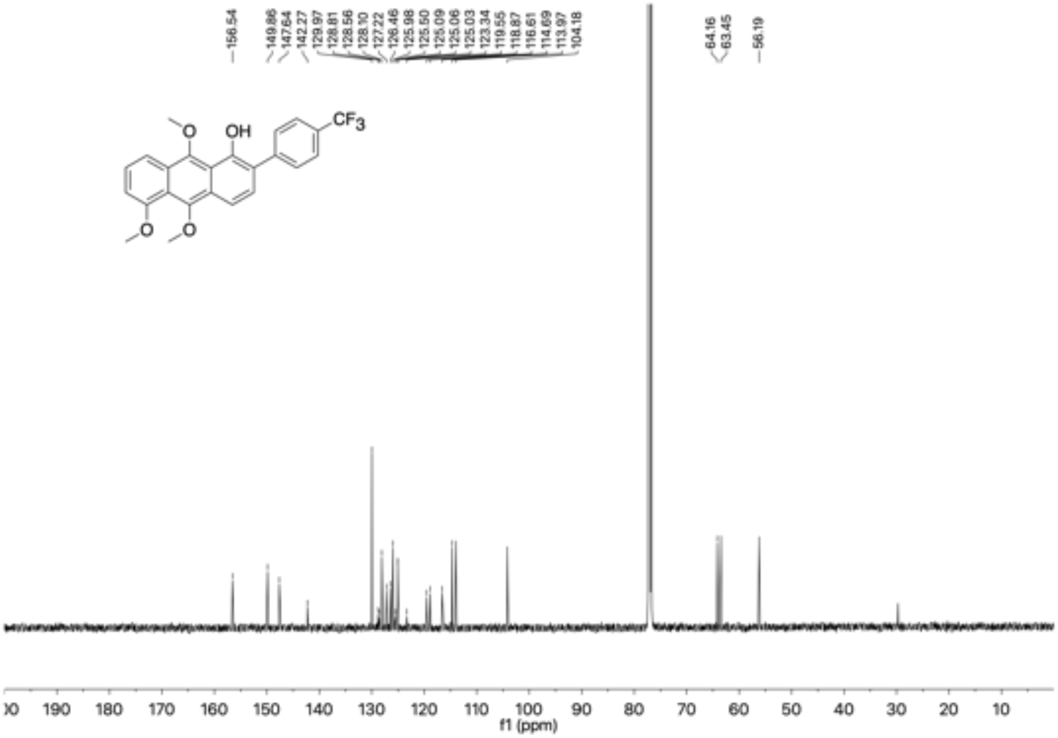

126 MHz, ^{13}C-NMR of substrate **S14b** in $CDCl_3$

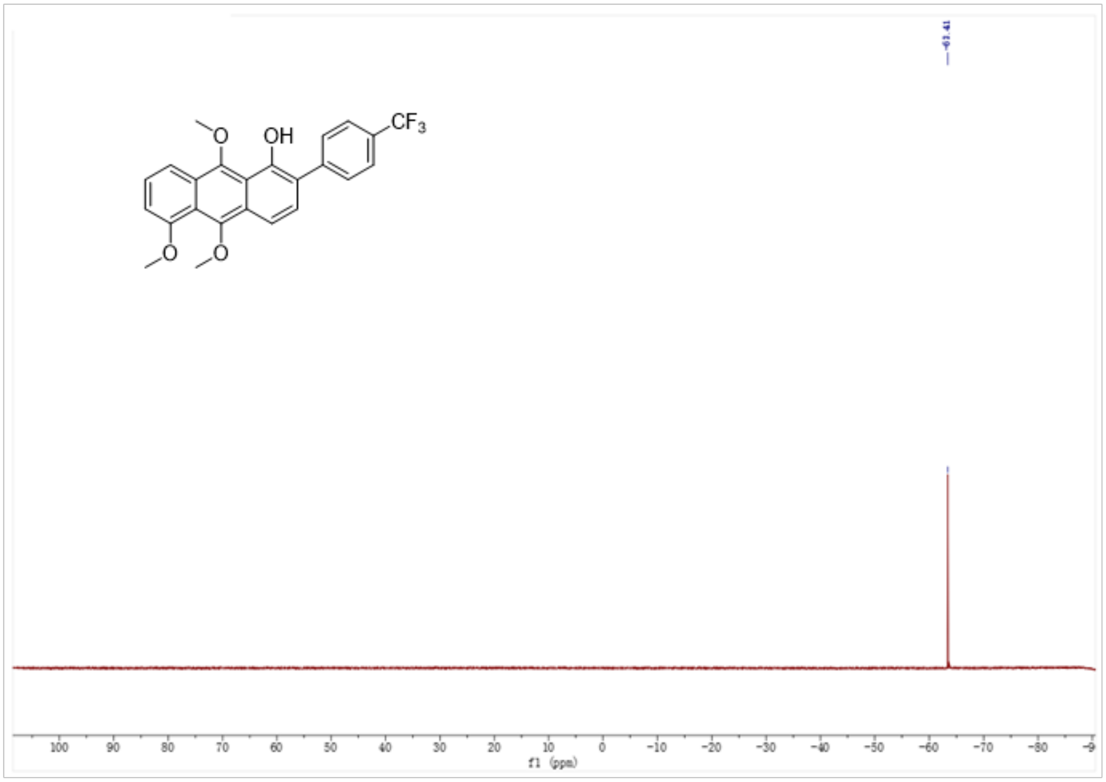

471 MHz, ^{19}F-NMR of substrate **S14b** in $CDCl_3$

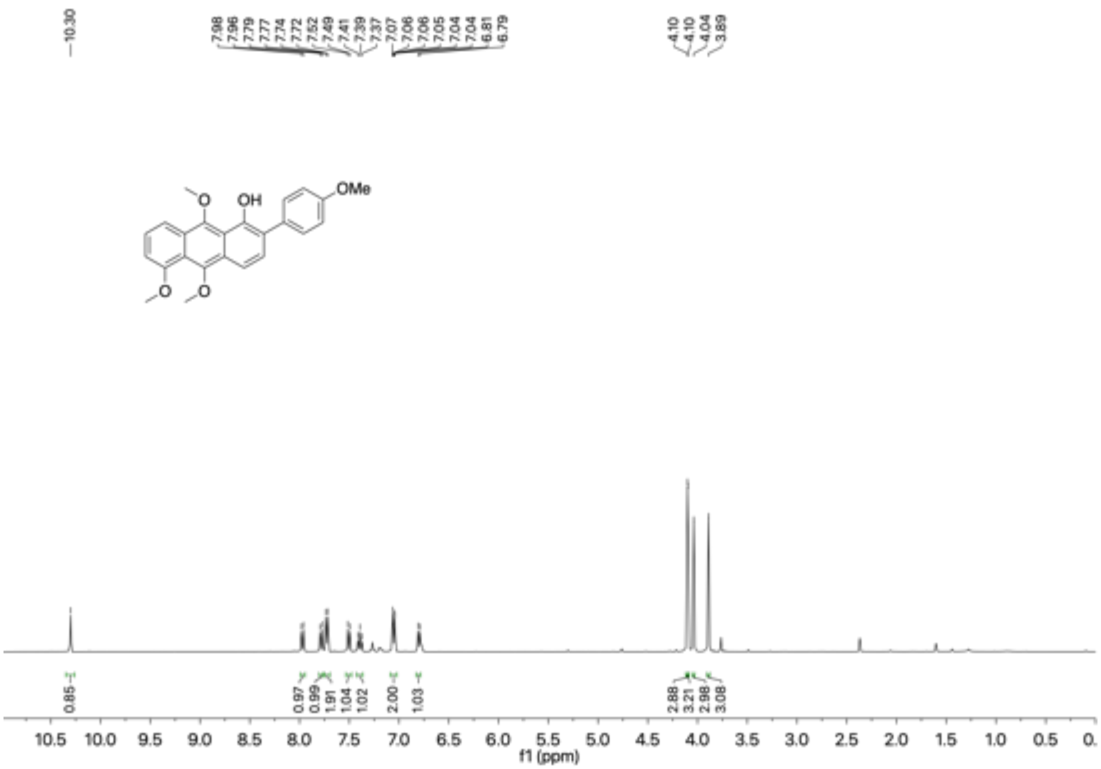

500 MHz, ^{1}H-NMR of substrate **S14c** in $CDCl_3$

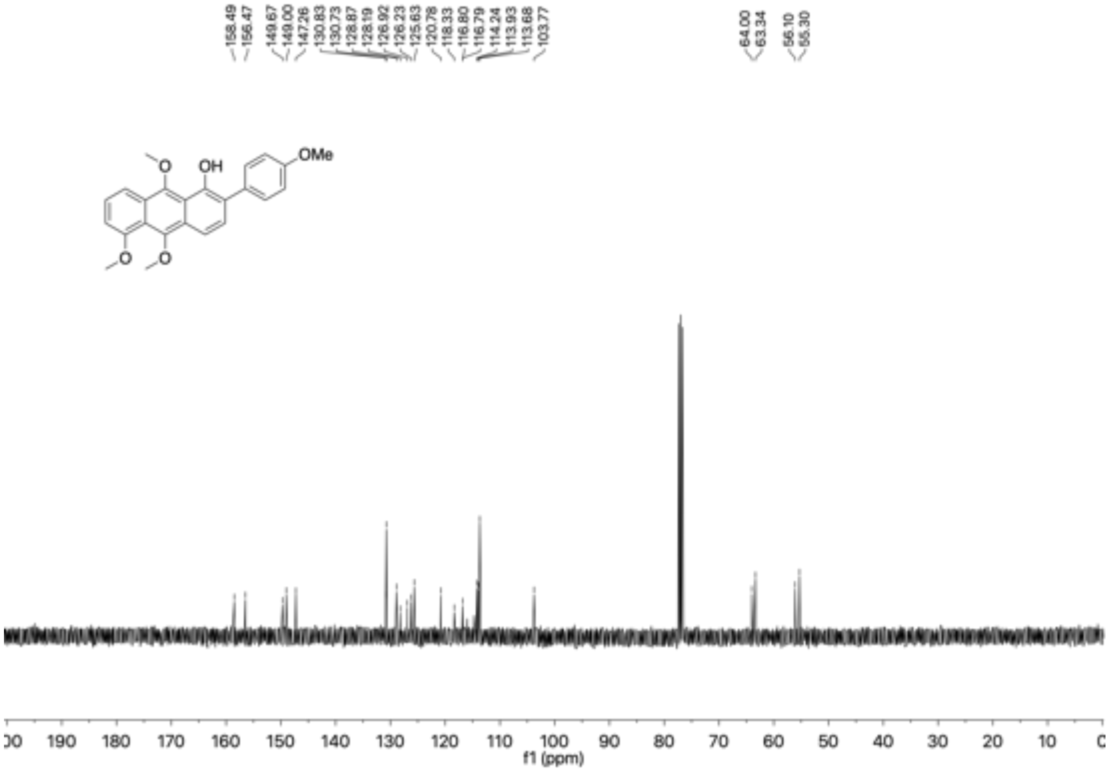

126 MHz, ^{13}C-NMR of substrate **S14c** in $CDCl_3$

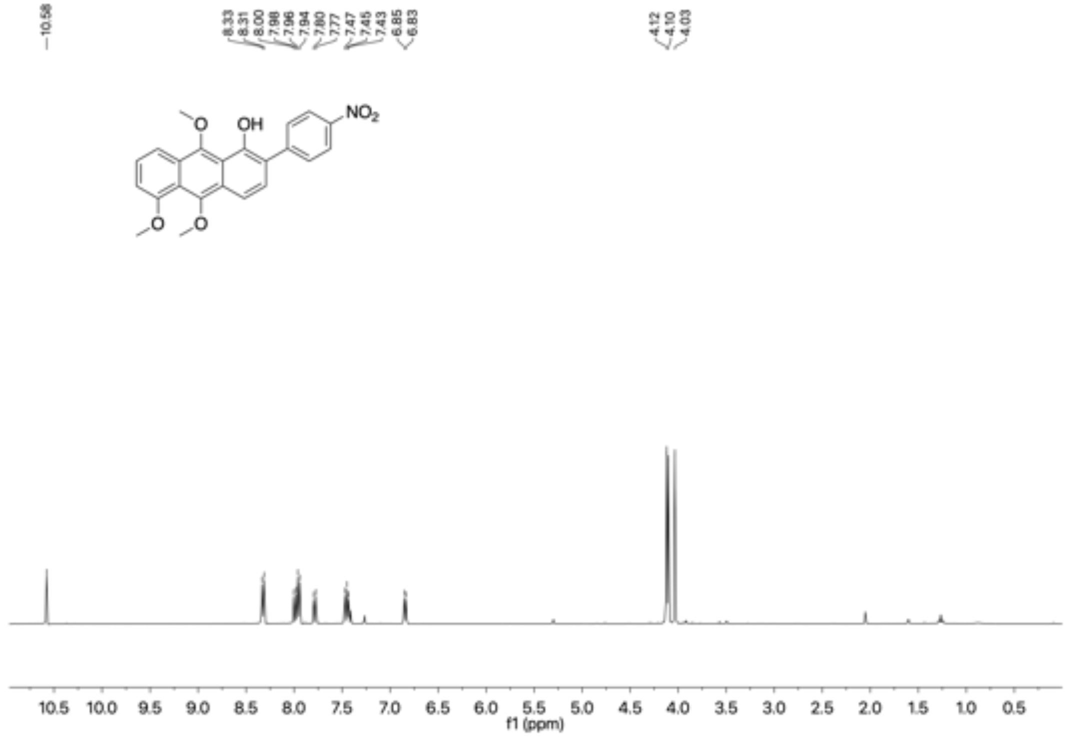

500 MHz, ^{1}H-NMR of substrate **S14d** in $CDCl_3$

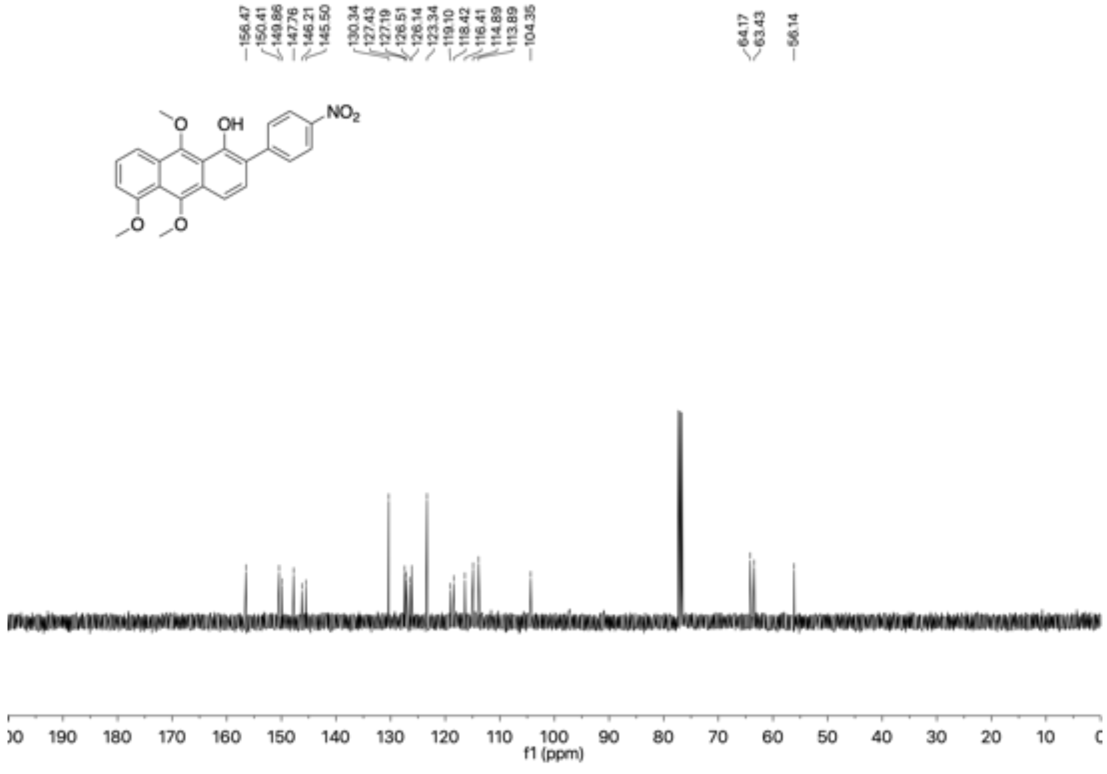

126 MHz, ^{13}C-NMR of substrate **S14d** in $CDCl_3$

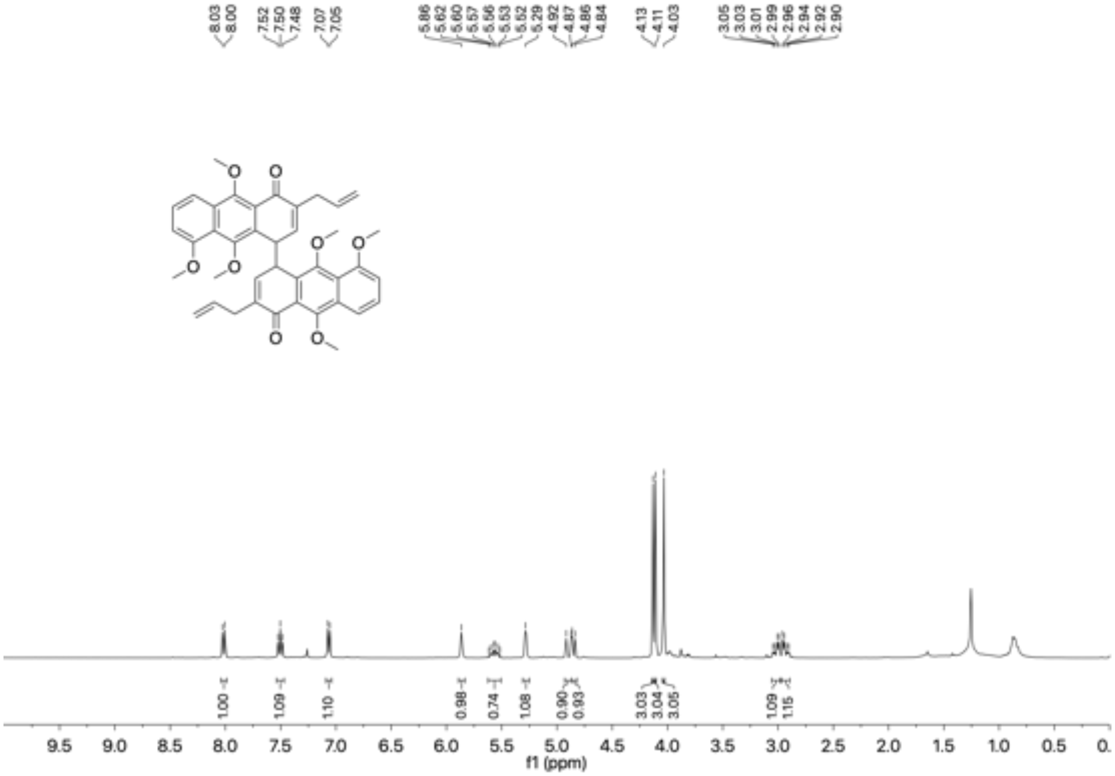

400 MHz, ^{1}H-NMR of substrate **15** in $CDCl_3$

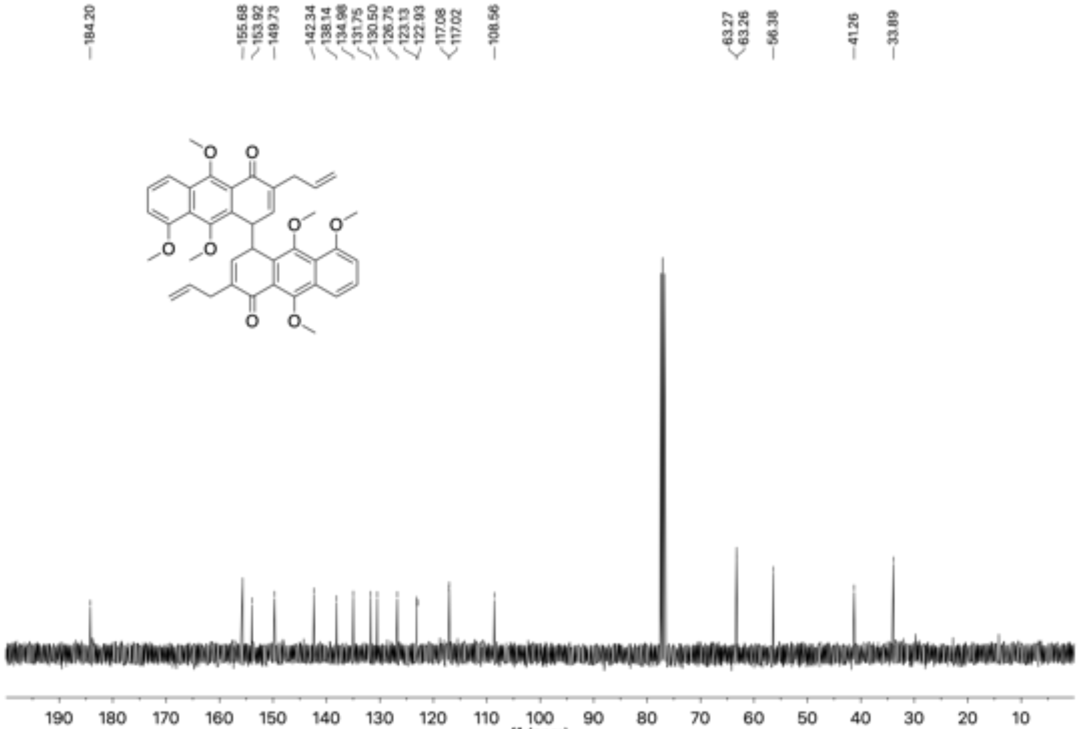

101 MHz, ^{13}C-NMR of substrate **15** in $CDCl_3$

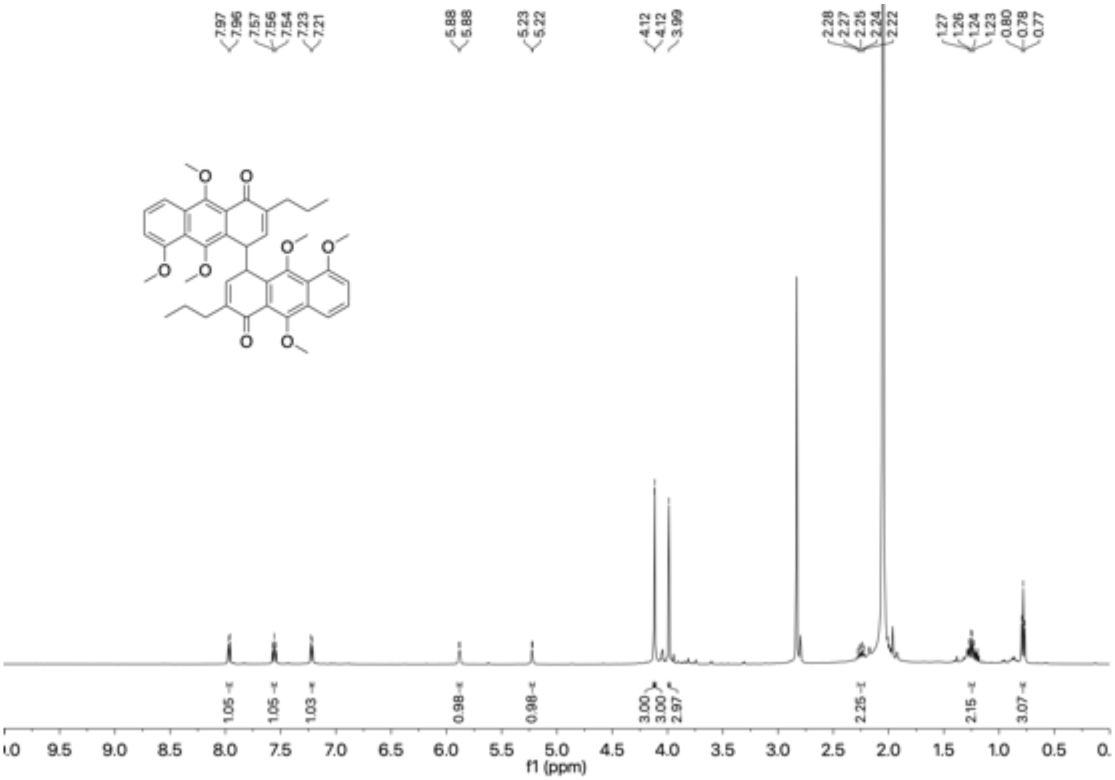

500 MHz, ^{1}H-NMR of substrate **16** in $CDCl_3$

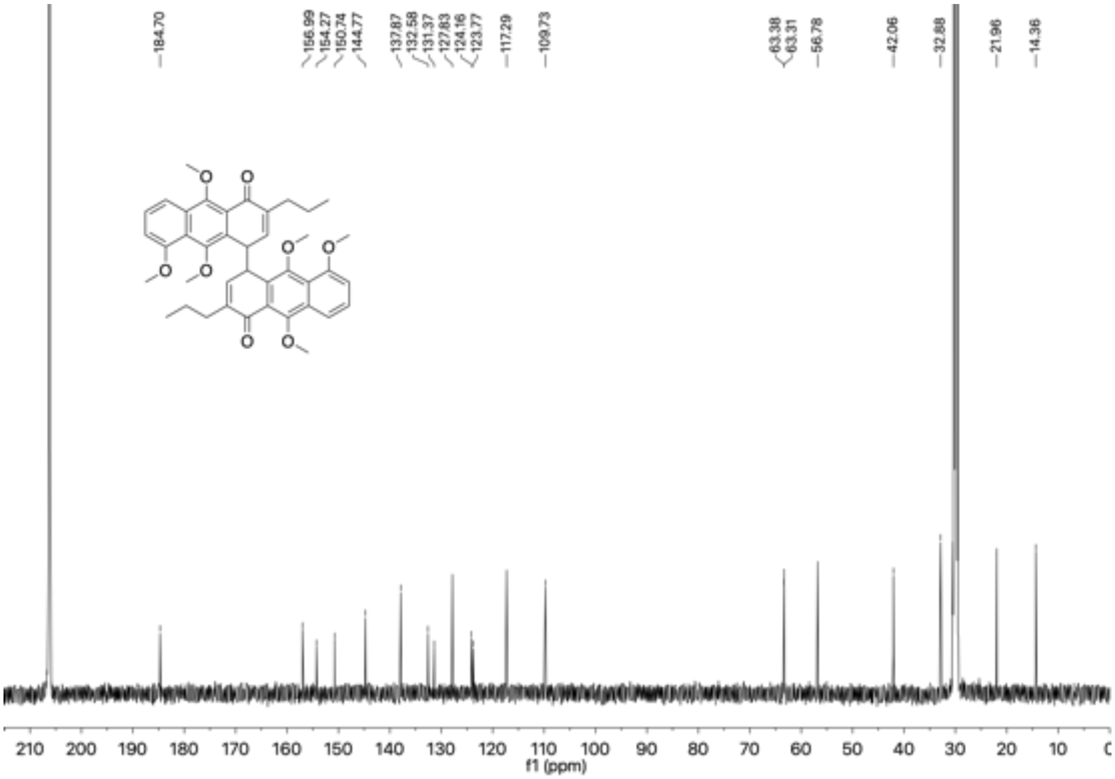

126 MHz, ^{13}C-NMR of substrate **16** in $CDCl_3$

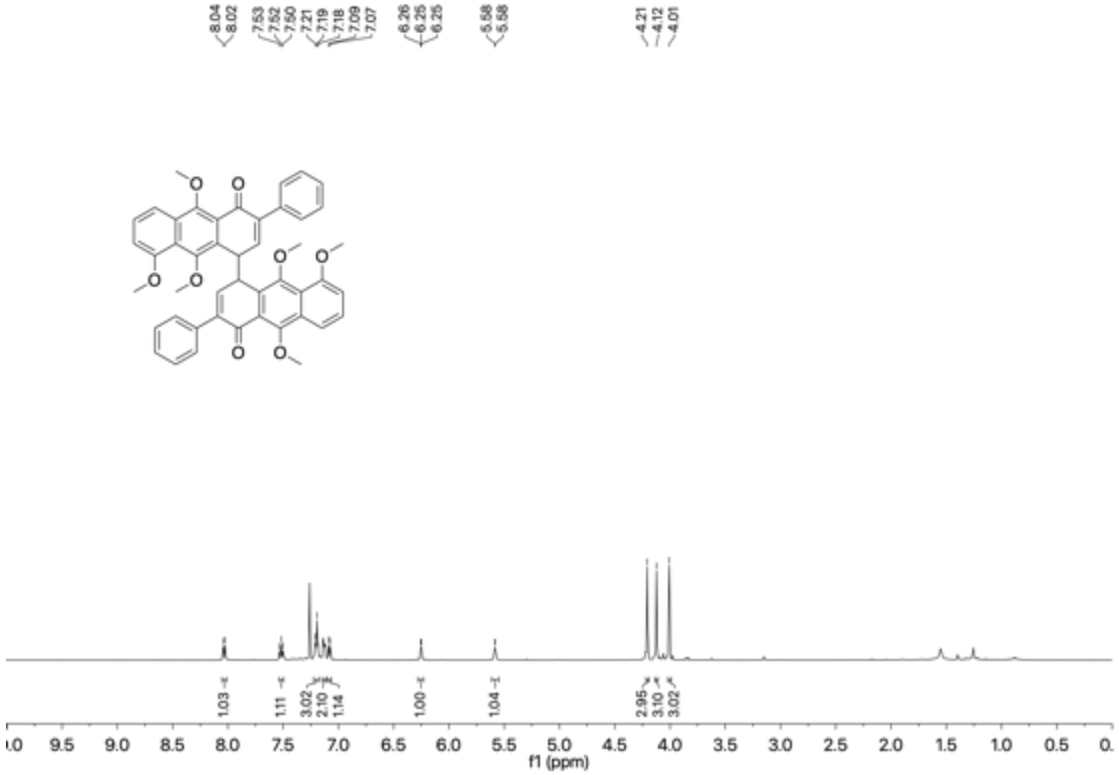

500 MHz, ^{1}H-NMR of substrate **17** in $CDCl_3$

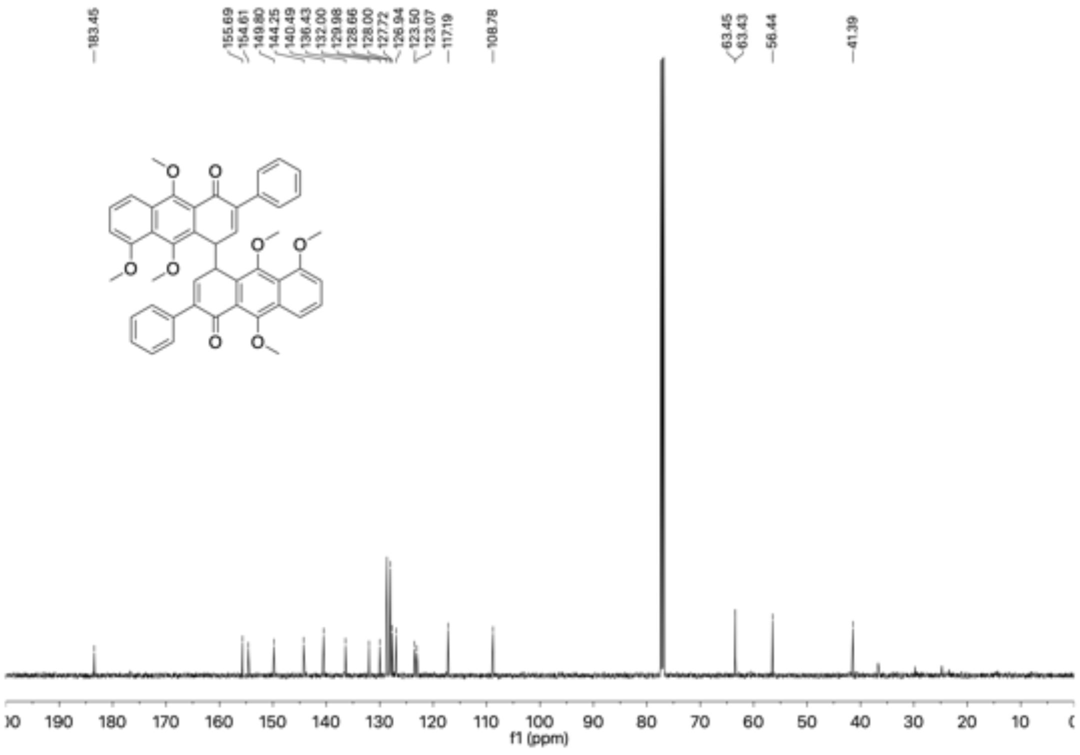

126 MHz, ^{13}C-NMR of substrate **17** in $CDCl_3$

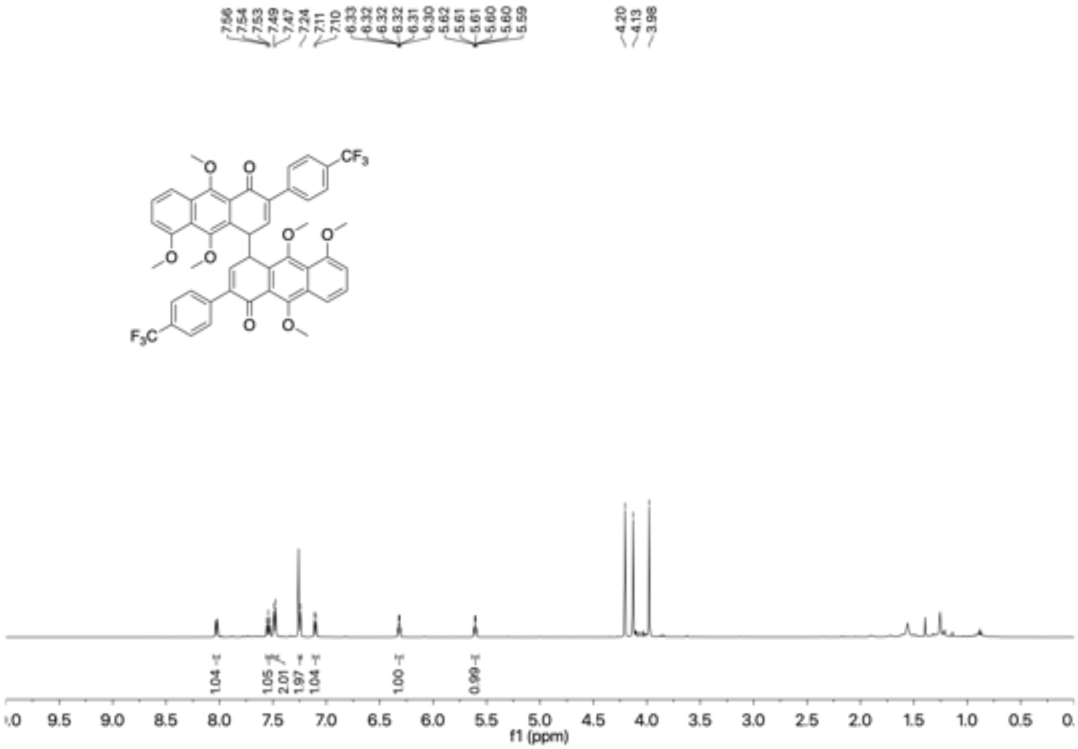

500 MHz, ^{1}H-NMR of substrate **18** in $CDCl_3$

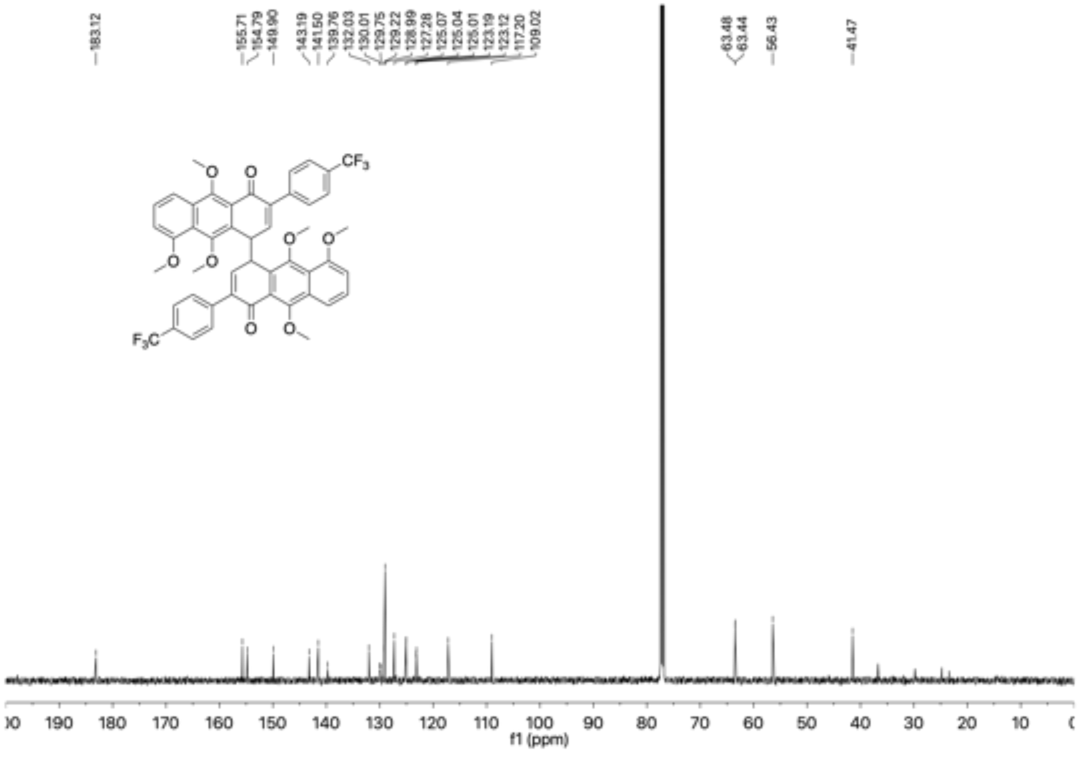

126 MHz, ^{13}C-NMR of substrate **18** in $CDCl_3$

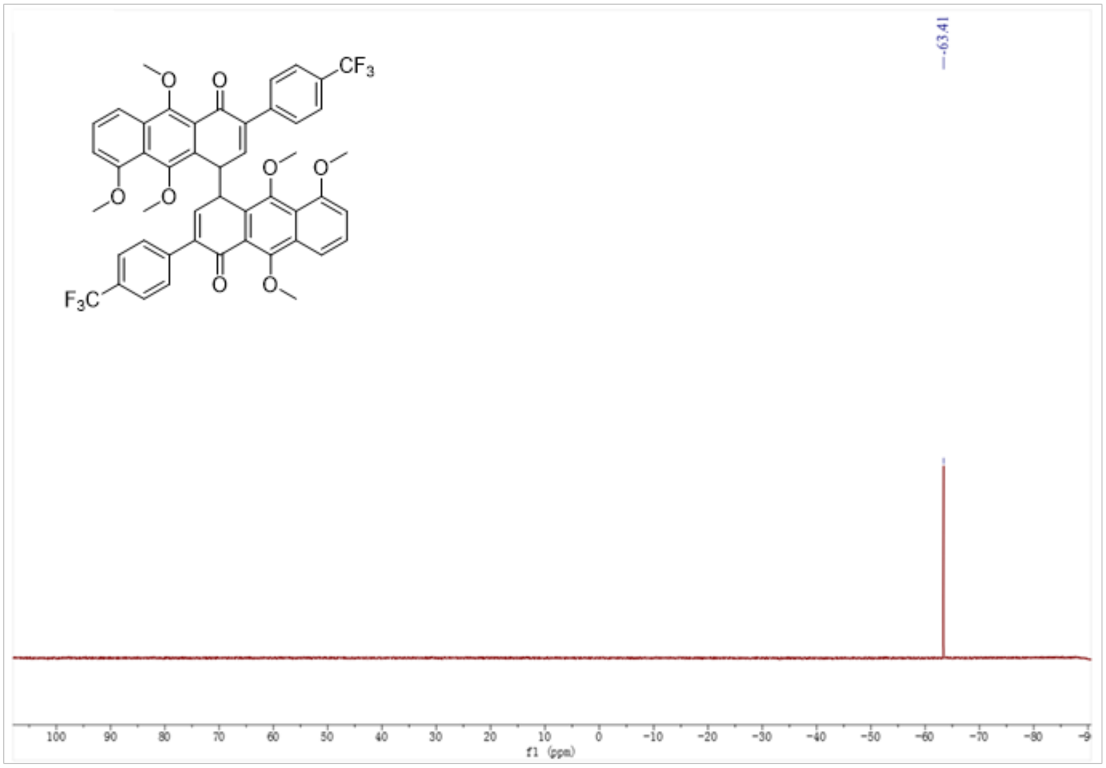

471 MHz, ^{19}F-NMR of substrate 18 in $CDCl_3$

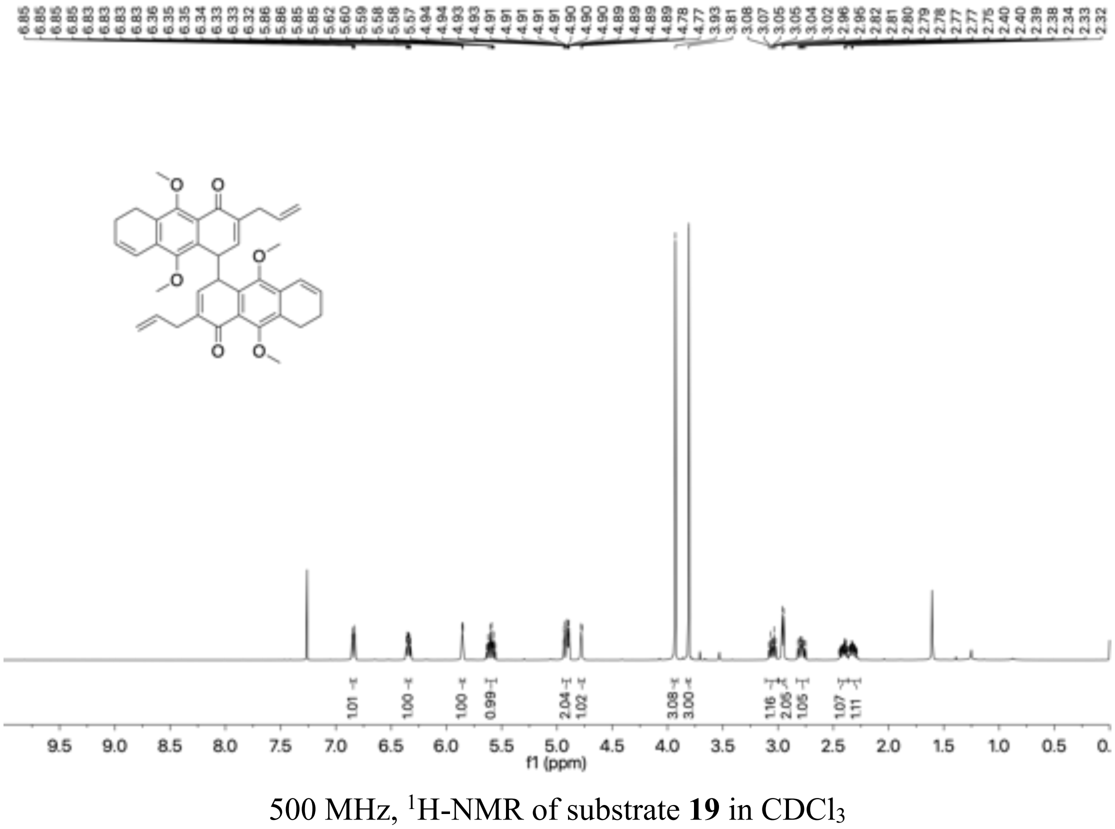

500 MHz, ^{1}H-NMR of substrate **19** in $CDCl_3$

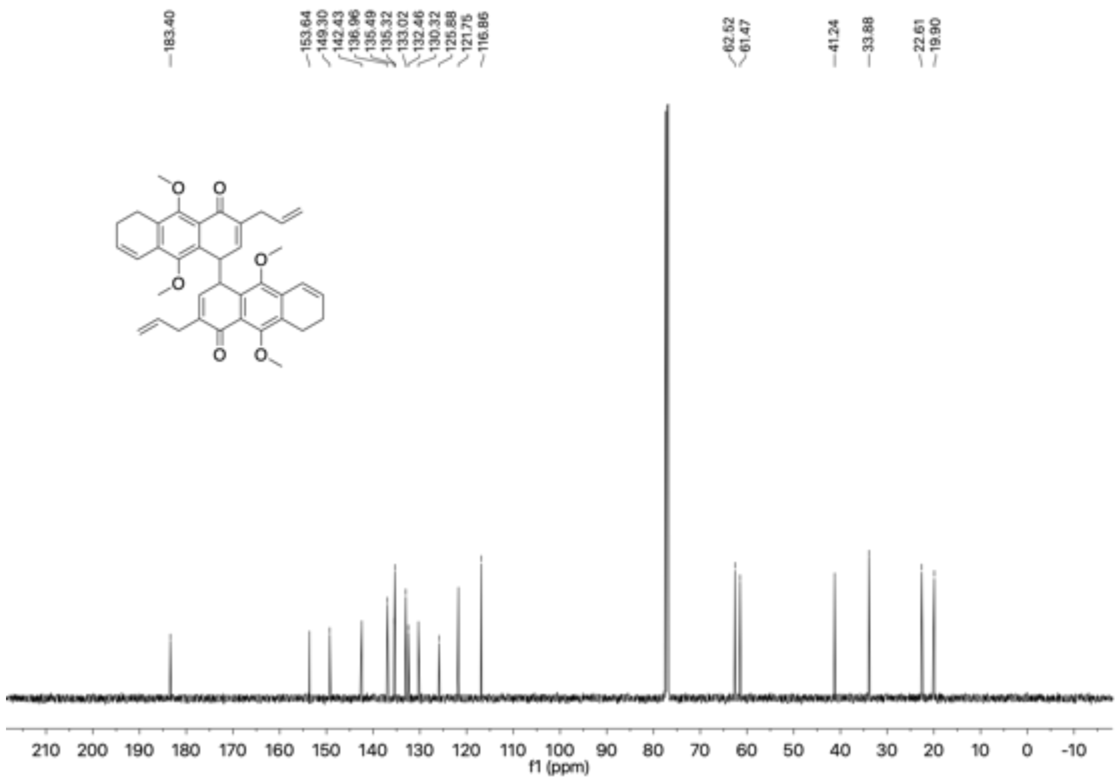

126 MHz, ^{13}C-NMR of substrate **19** in $CDCl_3$

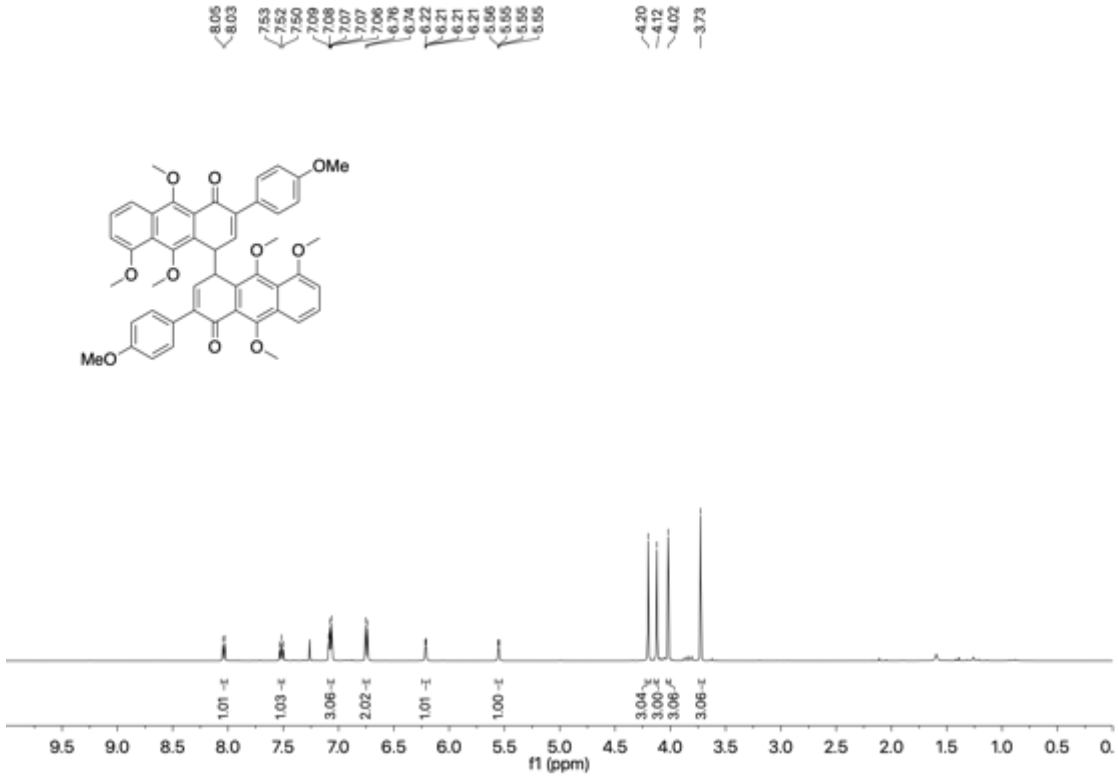

500 MHz, ^{1}H-NMR of substrate **20** in $CDCl_3$

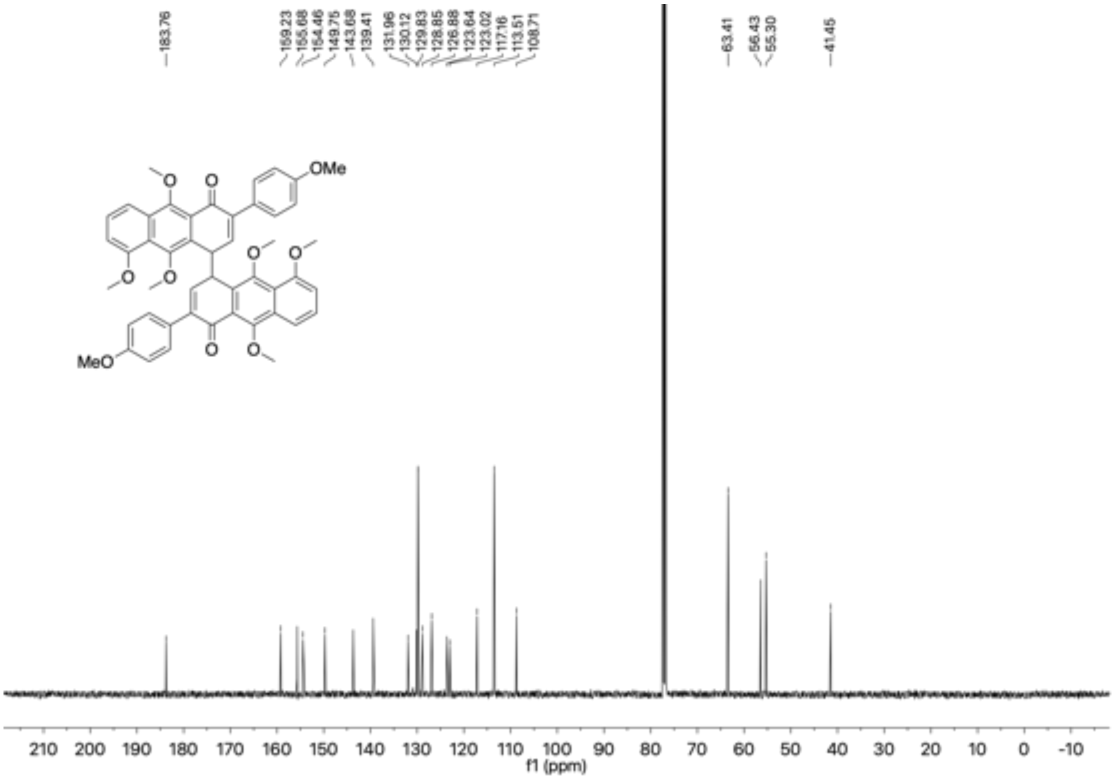

126 MHz, ^{13}C-NMR of substrate **20** in $CDCl_3$

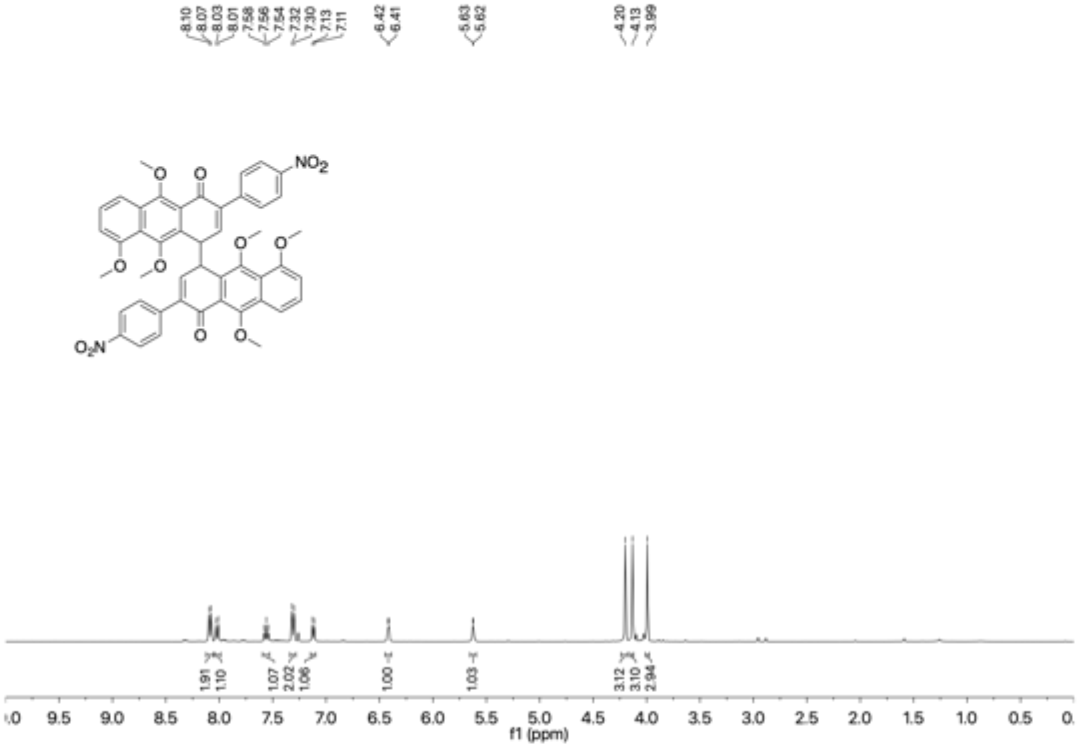

500 MHz, ^{1}H-NMR of substrate **21** in $CDCl_3$

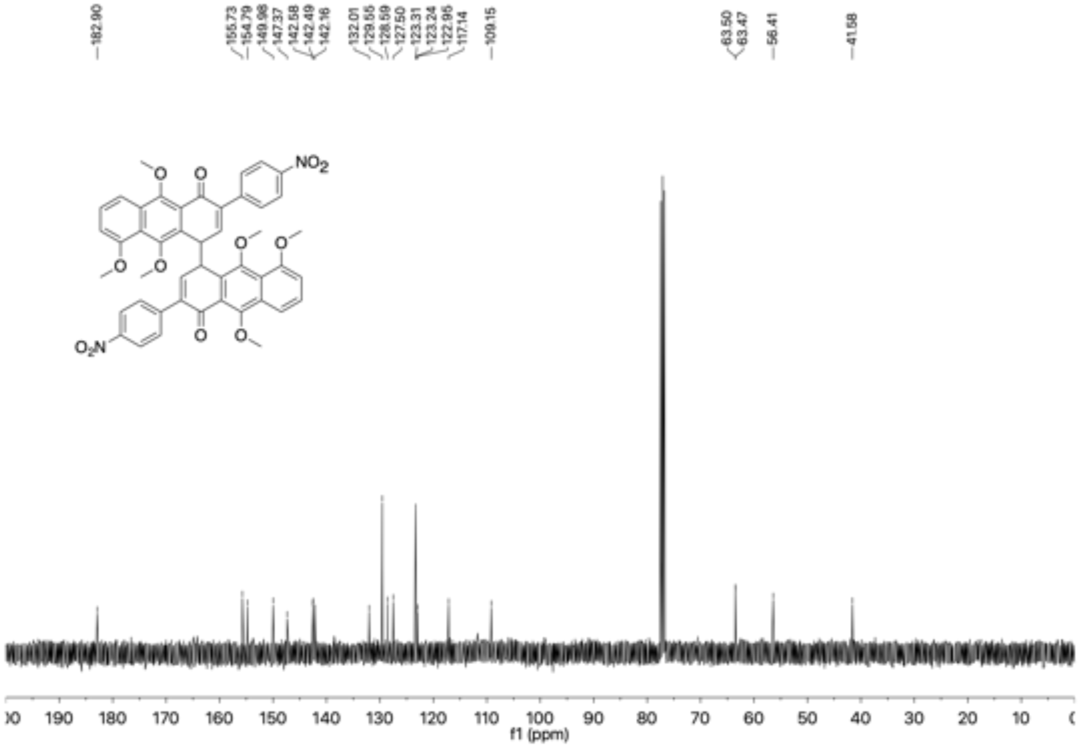

126 MHz, ^{13}C-NMR of substrate **21** in $CDCl_3$
